# Calhm6 Governs Macrophage Polarization Through Chp1‐Camk4‐Creb1 Axis and Ectosomal Delivery in Inflammatory Responses

**DOI:** 10.1002/advs.202502395

**Published:** 2025-09-26

**Authors:** Yanlong Xin, Xiaofan Xiong, Yan Zhang, Siyu Zhang, Shuting Zhang, Yu Yang, Yingxue Liang, Lulu Zang, Xi Chen, Wenjuan Li, Issam Halalmeh, Rui Zhou, Zongfang Li, Haowen Liu, Jing Geng

**Affiliations:** ^1^ Department of General Surgery the Second Affiliated Hospital Xi'an Jiaotong University Xi'an 710004 China; ^2^ National‐Local Joint Engineering Research Center of Biodiagnosis & Biotherapy the Second Affiliated Hospital Xi'an Jiaotong University Xi'an 710004 China; ^3^ International Joint Research Center on Cell Stress and Disease Diagnosis and Therapy the Second Affiliated Hospital Xi'an Jiaotong University Xi'an 710004 China; ^4^ Precision Medicine Institute the Second Affiliated Hospital Xi'an Jiaotong University Xi'an 710004 China; ^5^ Shaanxi Provincial Clinical Medical Research Center for Liver and Spleen Diseases the Second Affiliated Hospital Xi'an Jiaotong University Xi'an 710004 China; ^6^ Shaanxi International Cooperation Base for Inflammation and Immunity the Second Affiliated Hospital Xi'an Jiaotong University Xi'an 710004 China; ^7^ Shaanxi Provincial Academician Workstation the Second Affiliated Hospital Xi'an Jiaotong University Xi'an 710004 China; ^8^ School of Life Sciences and Technology Xi'an Jiaotong University Xi'an 710049 China

**Keywords:** Calhm6, ectosomes, inflammatory response, ion channel, macrophage polarization

## Abstract

Macrophage plasticity, critical for immune response, is often dysregulated in various infectious and inflammatory diseases. While ion channels have been implicated in immune cell modulation, how they influence macrophage polarization remains poorly understood. Here, it is demonstrated that ectosomes carrying the ion channel Calhm6 effectively suppress severe inflammation triggered by LPS. These Calhm6‐bearing ectosomes, secreted by macrophages, facilitate M2‐like polarization, elicit an anti‐inflammatory response, and foster immune tolerance. Conversely, Calhm6 deficiency leads to suppressed Creb1 activity, which in turn augments M1‐like macrophage polarization, enhancing bactericidal activity and the secretion of pro‐inflammatory cytokines. Mechanistically, Chp1 serves as a scaffold protein and undergoes phosphorylation by CaMK4. This phosphorylation enhances the localization of the Calhm6‐Chp1‐CaMK4 complex to the cell membrane, promoting Creb1 activation and M2‐like macrophage polarization calcium‐dependently. Moreover, the M1‐like polarization inducers LPS and IFNγ enhance the binding of Irf1 to the Calhm6 promoter, upregulating its expression and stimulating ectosome formation. Conversely, Stat6, activated by IL‐4, competes with Irf1 for binding to the Calhm6 promoter, thereby suppressing its expression. In summary, our findings unravel the intricate interplay between ion channels, ectosomes, and macrophage polarization, revealing that ectosomal‐Calhm6 can serve as a novel therapeutic agent to modulate inflammatory responses and facilitate tissue repair.

## Graphical abstract

Calhm6 drives M2 macrophage polarization via the Chp1‐Camk4‐Creb1 axis, suppressing inflammation through calcium‐dependent ectosomal delivery. Calhm6 deficiency enhances M1 responses, boosting bactericidal activity but exacerbating tissue damage. LPS/IFNγ upregulate Calhm6 via Irf1, while IL‐4/Stat6 inhibits it, balancing immune outcomes. Ectosomal Calhm6 emerges as a therapeutic candidate for inflammatory diseases and tissue regeneration.

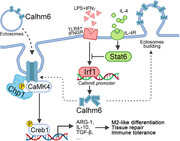

## 1 Introduction

Macrophages play a pivotal role in the immune system's early response to pathogen infections. They can polarize into distinct phenotypes, including M1‐like (classically activated) and M2‐like (alternatively activated) macrophages, each contributing to inflammation and infection defense or promoting tissue repair and immune tolerance.^[^ ^1^ ^]^ Ion channels are crucial in the inflammatory process, not only by mediating cellular electrical signaling but also by regulating the balance of ions across cellular membranes.^[^ ^2^, ^3^, ^4^ ^]^ Despite this, the precise mechanisms through which ion channels influence macrophage polarization and their responses to infection and inflammation remain poorly understood.^[^ ^5^, ^6^, ^7^, ^8^ ^]^

Extracellular vesicles, including exosomes and ectosomes, are essential for intercellular communication, particularly in the context of macrophage responses to infections and inflammation. Recent studies highlight the significant role of exosomes in conditions such as sepsis and autoimmune diseases.^[^ ^9^, ^10^ ^]^ While exosomes and ectosomes are often considered functionally similar, they differ markedly in their molecular contents. Ectosomes are particularly enriched in membrane proteins and phospholipids, including ion channels,^[^ ^11^, ^12^ ^]^ yet the precise role of ectosomes in regulating inflammation and macrophage polarization remains poorly defined.

CALHM6 (FAM26F) belongs to the evolutionarily conserved calcium homeostasis modulator (CALHM) family, a group of large‐pore ion channels characterized by transmembrane domains forming non‐selective pores (>1 nm) that permeate ions and metabolites such as ATP and glucose, placing them within the broader large‐pore channel superfamily.^[^ ^13^, ^14^, ^15^, ^16^ ^]^ These channels lack classical voltage‐sensing domains but are dynamically regulated by extracellular calcium levels and membrane voltage, enabling bidirectional transport critical for intercellular communication and metabolic homeostasis.^[^ ^14^, ^17^ ^]^ Recent structural studies reveal that CALHM channels assemble as hexameric or octameric complexes with a central pore stabilized by lipid interactions, a hallmark shared with other large‐pore channel families (e.g., pannexins, connexins).^[^ ^13^, ^14^ ^]^ Among CALHM members, CALHM6 exhibits unique immune‐specific functions. It is upregulated in the colonic mucosa of ulcerative colitis patients and dynamically regulated during infections—reduced in active hepatitis B virus (HBV) infection but restored post‐recovery.^[^ ^18^, ^19^ ^]^ CALHM6 activates natural killer (NK) cells via interferon gamma (IFNγ) secretion, and shows expression variability across cancers and infectious diseases.^[^ ^20^, ^21^ ^]^ While CALHM1 and CALHM3 are primarily implicated in neuronal ATP release and taste perception,^[^ ^22^ ^]^ CALHM6's immune‐centric roles underscore its specialization within the CALHM family, bridging ion channel activity with immunometabolic regulation.^[^ ^13^, ^17^ ^]^ These findings suggest that Calhm6 plays a critical role in immune responses. However, our understanding of its function remains limited.

In this study, we demonstrate that ectosomes carrying the ion channel Calhm6 effectively mitigate severe inflammation induced by lipopolysaccharide (LPS). Further investigation reveals that the absence of Calhm6 results in the inactivation of cAMP‐responsive element‐binding protein 1 (Creb1), leading to increased M1‐like macrophage polarization, enhanced bactericidal activity, and elevated production of pro‐inflammatory cytokines. Mechanistically, calcineurin B homologous protein 1 (CHP1), as a scaffold protein, is phosphorylated by Calcium/calmodulin‐dependent protein kinase IV (CaMK4), which facilitates the assembly of a Calhm6‐CHP1‐CaMK4 complex at the cell membrane. This complex promotes Creb1 activation and induces M2‐like macrophage polarization in a calcium‐dependent manner. Additionally, classical M1‐like polarization stimuli such as LPS and IFNγ enhance the binding of interferon regulatory factor 1 (IRF1) to the Calhm6 promoter, leading to increased Calhm6 expression and ectosome release. Conversely, interleukin‐4 (IL‐4) signaling through STAT6 competes for binding to the Calhm6 promoter, inhibiting this process. Collectively, our findings elucidate the role of Calhm6 in regulating macrophage immune responses and propose that Calhm6‐enriched ectosomes could serve as potential therapeutic agents for controlling inflammation and promoting tissue repair.

## 2 Results

### 2.1 Ectosomes Carrying Calhm6 Suppress Severe Inflammation in Response to LPS Challenge

**Figure 1:**
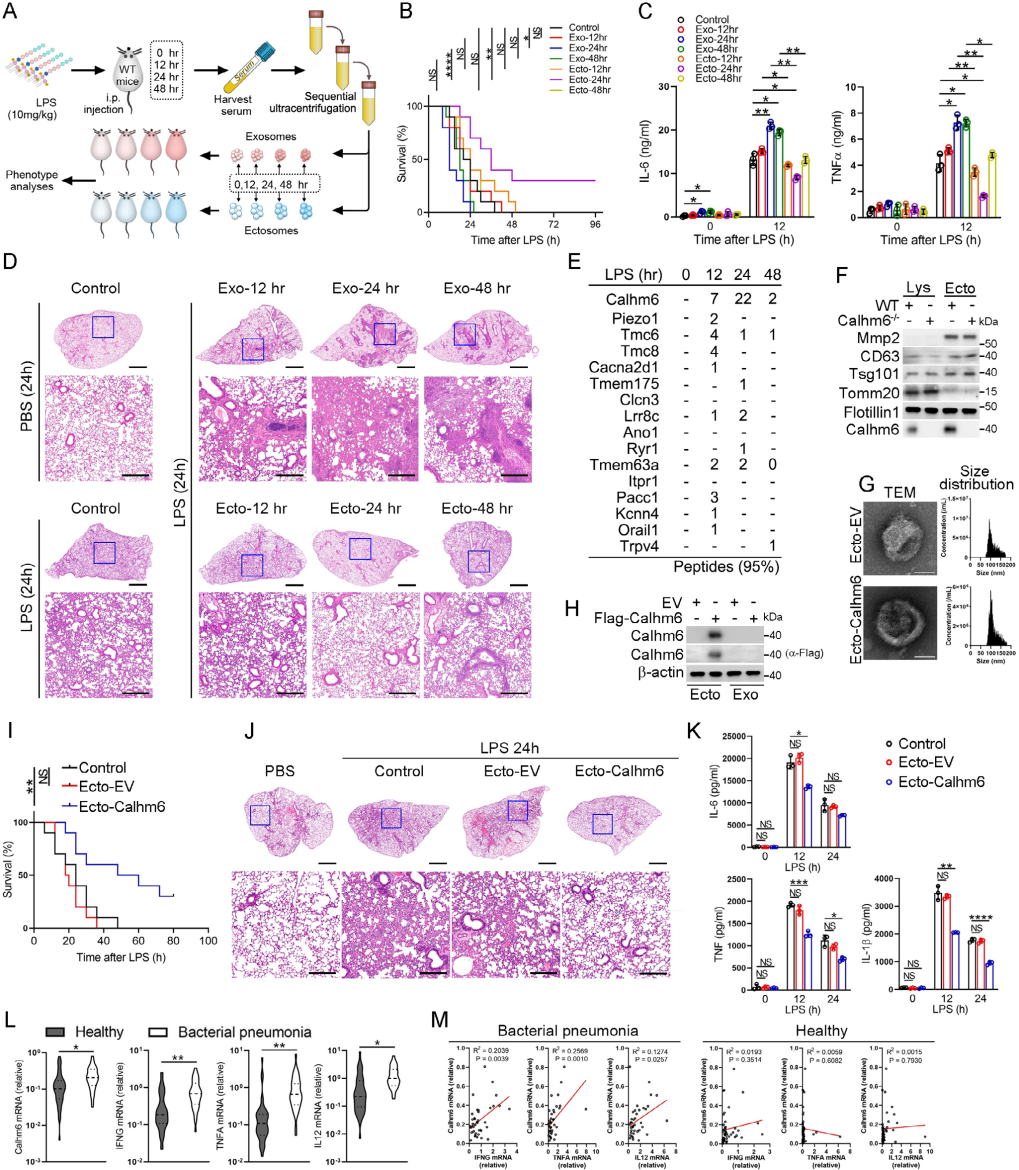
Ectosome carrying calhm6 suppresses severe inflammation by LPS challenging. A) The schematic diagram of collecting exosomes or ectosomes from the serum of wild‐type mice challenged by LPS at the indicated time through sequential ultracentrifugation. B–D) mortality (B), ELISA of serum cytokines (C), and H&E staining of the lungs (D) of wild‐type mice (*n* = 10 per group per experiment) pretreated with PBS (control), exosomes, or ectosomes before 48 h intraperitoneal injection of PBS or LPS (10 mg kg^−1^) for the indicated time. Scale bars (D), 2 mm or 200 µm. E) Mass spectrometry analysis of ectosome components in wild‐type mice serum after LPS treatment at the indicated time. F) Immunoblot analysis of Mmp2, CD63, Tsg101, Tomm20, Flotillin1, and Calhm6 in ectosomes purified from culture supernatant of wild‐type or Calhm6^–/–^ BMDMs; right, immunoblot analysis of total cell lysates (Lys) without centrifugation. G) The morphology of ectosomes was visualized using transmission electron microscopy (TEM). A NanoFCM particle size analyzer (N30E) was used to analyze the particle size of the ectosomes isolated from the EV and OE‐Calhm6 cells. Scale bars, 50 nm. H) Immunoblot analysis of Calhm6 and β‐actin in Raw264.7 cells incubated with ectosomes and exosomes from OE‐Calhm6 and control cells (α‐Flag) (right margin). I–K) mortality (I), H&E staining of inflammatory‐cell infiltration and injury in the lungs(J), ELISA of serum cytokines (K) of wild‐type mice (*n* = 10 per group per experiment) per‐treated with PBS (control), ectosomal‐EV or ectosomal‐Calhm6 before 48 h of intraperitoneal injection of PBS or LPS (10 mg kg^−1^) for indicated time. Scale bars (J), 2 mm or 200 µm. L,M) RT‐qPCR analysis expression of CALHM6, IFNG, TNFA, and IL‐12 mRNA in CD11b+ macrophages isolated from PBMCs of healthy donors (*n* = 47) and patients with bacterial pneumonia (BP; *n* = 39) (L); and correlation results were plotted and analyzed with the linear‐regression *t*‐test (M). The data represent the mean ± S.D. (*n* = 3). NS, not significant (*p* > 0.05); *p < 0.05, **p < 0.01, and ***p < 0.001, ****p < 0.00001 compared with control, Student's t‐test.

To investigate the role of extracellular vesicles in modulating inflammatory responses, we injected lipopolysaccharide (LPS) into wild‐type mice and collected serum samples at 0, 12, 24, and 48 h post‐injection. Exosomes and ectosomes were isolated from the serum using ultracentrifugation and subsequently injected into LPS‐challenged mice to evaluate their effects on inflammation (**Figure** **1**A). Strikingly, intraperitoneal administration of ectosomes isolated at the 24‐h time point significantly improved survival rates in LPS‐treated mice (Figure 1B), decreased serum levels of IL‐6 and TNFα (Figure 1C), and mitigated lung injury (Figure 1D). To explore the underlying mechanism by which ectosomes mitigate inflammation, we performed mass spectrometry analysis on ectosomes isolated from serum samples of wild‐type mice treated with LPS for 0, 12, 24, and 48 h. We found that the ion channel of Calhm6 is significantly induced by LPS and wrapped in ectosomes (Figure 1E; Figure S1A, Supporting Information). Importantly, ectosomes derived from Calhm6 knockout mice failed to suppress pro‐inflammatory cytokine production, resulting in elevated levels of IL‐6 and TNFα (Figure 1F; Figure S1B, Supporting Information). Furthermore, Calhm6 expression in ectosomes from wild‐type mice peaked 24 h after LPS treatment (Figure S1C, Supporting Information).

Expression profiling demonstrated high levels of Calhm6 mRNA in the spleen, bone marrow, and peripheral blood mononuclear cells (PBMCs) (Figure S1D, Supporting Information), consistent with data from The Human Protein Atlas, which highlights CALHM6 expression in the hematological system, particularly in macrophages. We next generated RAW 264.7 cells overexpressing GFP‐flagged Calhm6 (OE‐Calhm6) and control cells (empty vector, EV). OE‐Calhm6 cells secreted ectosomes enriched in Calhm6, which were confirmed to be within the 100–200 nm size range (Figure 1G). These ectosomes were efficiently internalized by RAW 264.7 cells (Figure 1H) and restored Calhm6 expression in Calhm6‐knockout bone marrow‐derived macrophages (BMDMs) (Figure S1E, Supporting Information). To assess ectosome‐mediated transfer of Calhm6 in vivo, GFP‐Calhm6 ectosomes were intravenously injected into mice, and GFP‐positive immune cells were tracked (Figure S1F, Supporting Information). Flow cytometry analysis revealed that macrophages, neutrophils, and dendritic cells, known for their phagocytic capabilities, were the primary cell types internalizing GFP‐positive ectosomes (Figure S1G, Supporting Information).

Administration of ectosomal‐Calhm6 from OE‐Calhm6 cells improved survival in LPS‐challenged wild‐type mice (Figure 1I), reduced lung injury (Figure 1J), and lowered pro‐inflammatory cytokine levels (Figure 1K). To validate the correlation between Calhm6 and acute inflammation in humans, we found that mRNA levels of IFNG, TNFA, IL‐12, and Calhm6 in CD11b^+^ macrophages isolated from the peripheral blood of bacterial pneumonia patients were significantly elevated compared to healthy controls (Figure 1L). Furthermore, Calhm6 expression positively correlated with the expression of IFNG, TNFA, and IL‐12 (Figure 1M). Similarly, in mouse BMDMs treated with *Escherichia coli*, *Staphylococcus aureus*, *Candida albicans*, or LPS, Calhm6 expression was significantly elevated after 24 h (Figure S1H, Supporting Information). Additionally, analysis of other CALHM family members revealed that Calhm1–5 showed minimal response to inflammatory stimuli such as lipoteichoic acid (LTA), LPS, bisacyl‐lipopeptide (FSL‐1), or Pam3CSK4, unlike Calhm6 (Figure S1I, Supporting Information). These findings suggest that Calhm6 responds rapidly to acute inflammation caused by infection and mitigates tissue damage and mortality through ectosome‐mediated delivery.

### 2.2 Ectosomal‐Calhm6 Mitigates Inflammation and Promotes Tissue Repair via M2‐Like Macrophage Polarization

**Figure 2:**
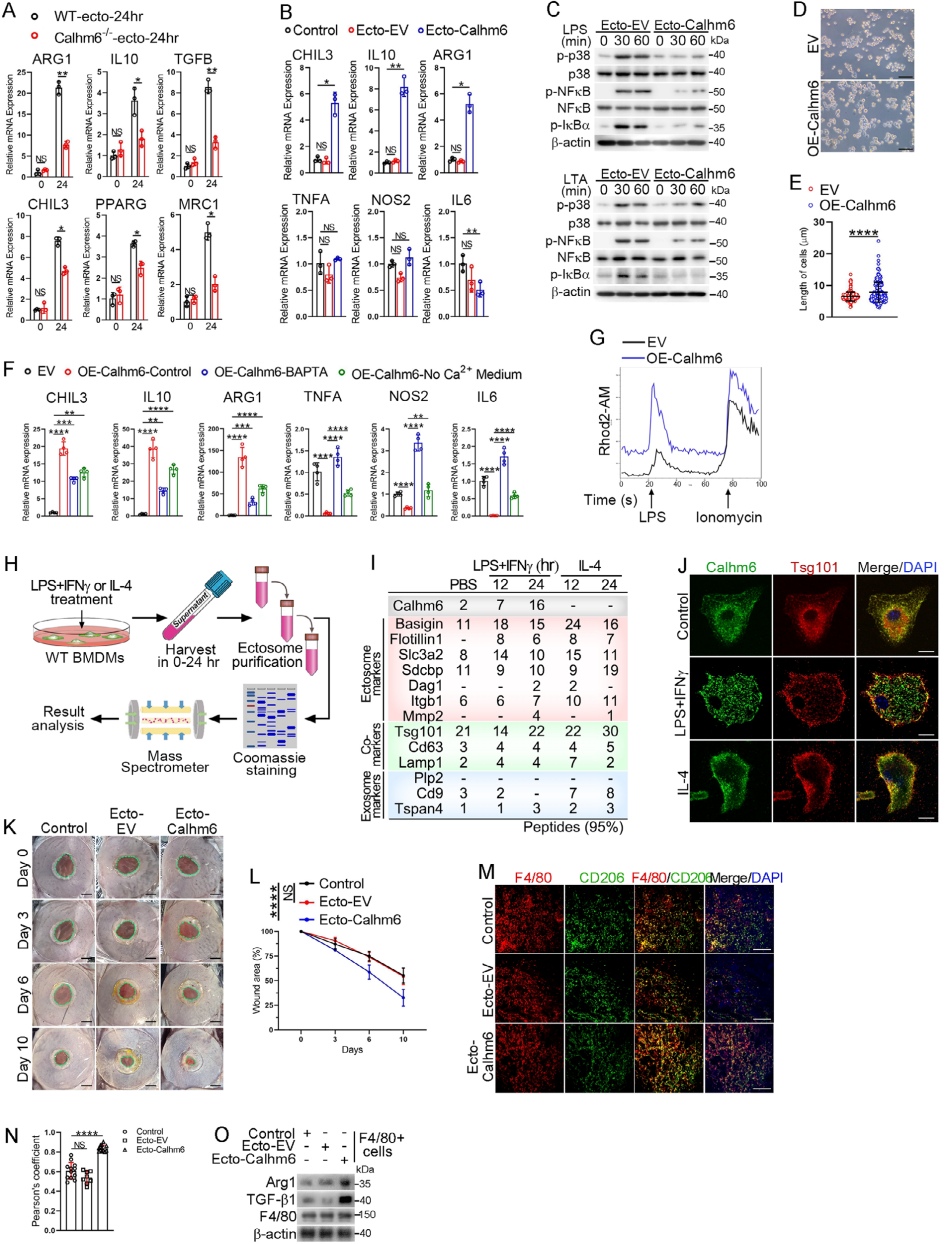
Ectosomal‐calhm6 mitigates inflammation and enhances tissue repair via M2‐like macrophage polarization. A) After 24 h of LPS treatment, ectosomes were collected from the serum of wild‐type or Calhm6 knockout mice. These exosomes were used to treat wild‐type BMDMs, and the expression of M2‐like markers in the BMDMs was analyzed using RT‐PCR. B) RT‐PCR analysis of TNFA, NOS2, IL6, CHIL3, IL10, and ARG1 in wild‐type BMDMs incubated with or without ectosomes for 24 h from EV or OE‐Calhm6 cells. C) Immunoblot analysis of phosphorylated (p‐) p38, NFκB, IκBα, and β‐actin in wild‐type BMDMs stimulated for 0, 30, 60 min with LPS (200 ng mL^−1^) after incubation with ectosomal‐EV or ectosomal‐Calhm6 from EV or OE‐Calhm6 cells for 24 h. D,E) Morphology of EV or OE‐Calhm6 cells (D) and cell length (I) was measured by ImageJ (*n* =304). Scale bars(E), 100 µm. F) RT‐PCR analysis of CHIL3, IL10, ARG1, TNFA, NOS2 and IL6 in EV or OE‐Calhm6 cells treated with or without BAPTA‐AM (20 µm) or Ca^2+^‐free DMEM for 24 h. G) Calcium influx over time in EV or OE‐Calhm6 cells treated with LPS (200 ng mL^−1^) and stained by the Rhod2‐AM (4 µm, 30 min). H,I) Diagram (H) of ectosomes by sequential ultracentrifugation for mass spectrometry from serum of wild‐type BMDMs treatment with LPS (200 ng mL^−1^) and IFNγ (10 ng mL^−1^) combined or IL‐4 (20 ng mL^−1^) for 0, 12 or 24 h; the indicated genes identified were shown in the table (I). J) Fluorescence microscopy of the colocalization (white arrows) of Calhm6 (green) and Tsg101 (red) in wild‐type BMDMs treated with or without LPS, IFNγ combined or IL‐4 for 12 h. Scale bars, 10 µm. K) Representative photographs of the wounds on days 0, 3, 6, and 10 after parawound injection of PBS (control), ectosomal‐EV, ectosomal‐Calhm6 in the mouse excisional wound splinting model. Scale bars, 2 mm. L) Measurement of wound area at indicated points. The percentage of wound closure was calculated as: (area of wound at time/area of original wound×100%. M) Immunofluorescence histochemistry of frozen section of wound area of F4/80 (red), CD206 (green), and counterstained with DAPI (blue) in each group as indicated above at day 10. Scale bars, 200 µm. N) Pearson's correlation coefficient values for colocalization of CD206 and F4/80 in the wound area. The average Pearson's correlation coefficients were calculated from eight randomly selected infected cells in each group (*n* = 12 photos examined). O) Immunoblot analysis of Arg, Tgf‐β1, F4/80, and β‐actin in F4/80^+^ cells from the wound area for each group as indicated above at day 10. The data represent the mean ± S.D. (*n* =3). NS, not significant (*p* > 0.05); *p < 0.05, **p < 0.01, and ***p < 0.001, ****p < 0.00001 compared with control, Student's t‐test.

To investigate the mechanism by which ectosomal‐Calhm6 suppresses acute inflammation, reduces tissue damage, and improves survival, we isolated ectosomes from wild‐type or Calhm6 knockout mice challenged with LPS for 24 h, following the method described in Figure 1A. Incubation of these ectosomes with BMDMs revealed that ectosomes from wild‐type mice significantly increased the expression of M2‐like macrophage markers, such as arginase 1 (ARG1) and IL‐10. In contrast, ectosomes from Calhm6 knockout mice failed to induce such effects (**Figure** **2**A). Similarly, ectosomes derived from OE‐Calhm6 cells strongly upregulated M2‐like markers in macrophages (Figure 2B) while suppressing NF‐κB and MAPK signaling pathways activated by LPS or lipoteichoic acid (LTA) (Figure 2C). These findings suggest that ectosomal‐Calhm6 can suppress inflammatory signals by promoting M2‐like polarization in macrophages. Microscopic analysis showed that OE‐Calhm6 cells exhibited a spindle‐shaped morphology characteristic of M2‐like macrophages (Figure 2D,E). Furthermore, OE‐Calhm6 cells expressed higher levels of M2‐like factors in the presence of calcium (Figure 2F), suggesting that Calhm6 participates in the regulation of macrophage polarization in a calcium‐dependent manner. In line with this, we observed that LPS stimulation induced higher calcium influx in OE‐Calhm6 cells (Figure 2G). We also investigated whether the polarization state of macrophages influences the generation of Calhm6 in ectosomes. Interestingly, our findings revealed that only LPS/IFNγ treatment effectively encapsulated Calhm6 within ectosomes (Figure 2H,I). Additionally, LPS/IFNγ treatment significantly enhanced the colocalization of Calhm6 with tumor susceptibility gene 101 (TSG101), a marker of ectosome biogenesis (Figure 2J; Figure S2A, Supporting Information).

Given the critical role of M2‐like macrophages in immune tolerance and tissue repair, we tested the therapeutic potential of ectosomal‐Calhm6 in a mouse excisional wound splinting model. Ectosomal‐Calhm6 administration significantly improved wound healing (Figure 2K,L) by promoting the infiltration of CD206‐positive macrophages and increasing the expression of ARG1 and transforming growth factor beta 1 (TGF‐β1) in skin wounds (Figure 2M–O). In a dextran sodium sulfate (DSS)‐induced colitis model, wild‐type mice treated with ectosomal‐Calhm6 on alternate days exhibited significant resistance to colitis, as evidenced by improved disease activity index (DAI) scores, reduced colonic epithelial shedding, diminished inflammatory cell infiltration, and less weight loss and rectal bleeding (Figure S2B–D, Supporting Information). Similarly, in the acetaminophen (APAP)‐induced liver injury model, the combination of N‐acetylcysteine (NAC) and ectosomal‐Calhm6 effectively prevented the expansion of centrilobular necrotic lesions in the liver (Figure S2E; Figure S2F, Supporting Information).

In summary, Calhm6 promotes the polarization of macrophages toward an M2‐like phenotype in a calcium‐dependent manner. Ectosomal‐Calhm6, upon internalization by target macrophages, similarly drives M2‐like polarization, thereby alleviating inflammation and promoting tissue repair. These findings highlight the therapeutic potential of ectosomal‐Calhm6 in managing inflammatory diseases and facilitating tissue regeneration.

### 2.3 Loss of Calhm6 Enhances M1‐Like Macrophage Polarization via Creb1 Inactivation

**Figure 3:**
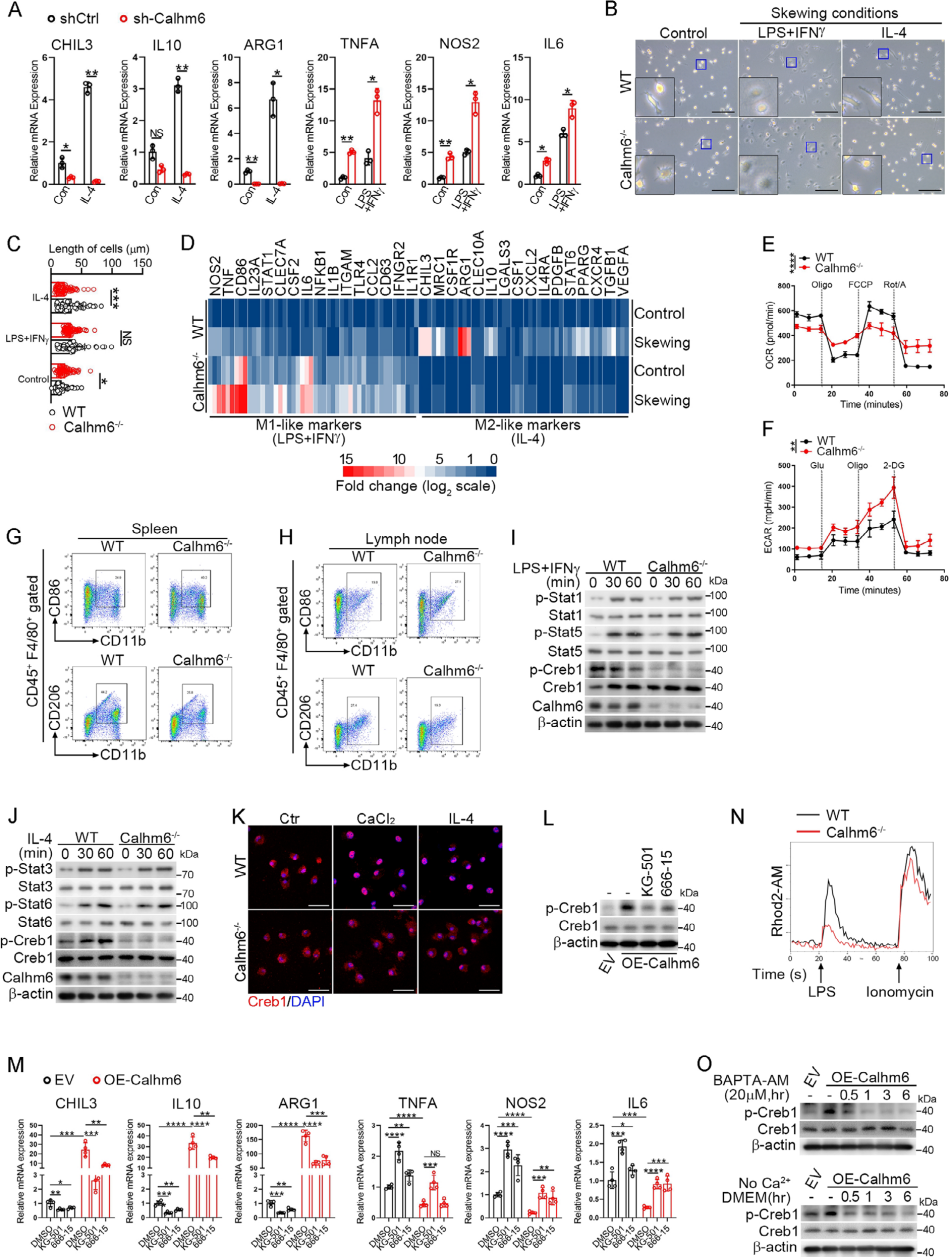
Loss of Calhm6 enhances M1‐like macrophages through Creb1 inactivation. **A**) RT‐qPCR analysis of CHIL3, IL10, ARG1, TNFA, NOS2, and IL6 in shCtrl or shCalhm6 cell line treated with LPS, IFNγ combined or IL‐4 for 24 h. B,C) Morphology of wild‐type or Calhm6 knockout BMDMs B) polarized to M1‐like or M2‐like phenotypes by treatment with LPS and IFNγ combined or IL‐4 for 24 h, and cell length C) was measured by ImageJ (*n* =55). Scale bars(C), 100 µm. D) Transcriptome analysis of M1‐of M2‐like markers expressed by LPS, IFNγ combined or IL‐4 for 24 h in wild‐type or Calhm6 knockout BMDMs. E,F) Seahorse analysis of OCR (E) and ECAR (F) in BMDMs form wild‐type or Calhm6 knockout mice. G,H) Flow cytometric analysis of CD86^+^/CD206^+^ macrophage populations in the spleen (G) and lymph node (H) of wild‐type and Calhm6 knockout mice, as determined with anti‐F4/80, anti‐CD11b, anti‐CD86, and anti‐CD206 antibodies. I,J) Immunoblot analysis of (p‐) Stat1, Stat3, Creb1, Stat5, Stat6, Calhm6, and β‐actin in wild‐type or Calhm6 knockout BMDMs stimulated for 0, 30, 60 min with LPS, IFNγ combined (I) or IL‐4 (J). K) Fluorescence microscopy of wild‐type or Calhm6 knockout BMDMs treated with CaCl_2_ (2 mm) or IL‐4 for 3 h, then washed, fixed, and immunostained with anti‐Creb1 (red) and counterstained with DAPI (blue). Scale bars, 50 µm. L) Immunoblot analysis of (p‐) Creb1 and β‐actin in EV or OE‐Calhm6 cells treated with or without KG‐501 or 666‐15. M) RT‐PCR analysis of CHIL3, IL10, ARG1, TNFA, NOS2, and IL6 in EV or OE‐Calhm6 cells treated with or without KG‐501 or 666‐15. N) Calcium influx over time in wild‐type or Calhm6 knockout BMDMs treated with LPS (200 ng mL^−1^) and stained by the Rhod2‐AM. O)Immunoblot analysis of (p‐) Creb1 and β‐actin in EV or OE‐Calhm6 cells treated with or without BAPTA‐AM (20 µm) or Ca^2+^‐free DMEM for 0.5, 1, 3, 6 h. The data represent the mean ± S.D. (*n *= 3). NS, not significant (*p* > 0.05); *p < 0.05, **p < 0.01, and ***p < 0.001, ****p < 0.00001 compared with control, Student's t‐test.

To elucidate the mechanism by which Calhm6 promotes M2‐like polarization of macrophages, we constructed a Calhm6 knockdown cell line in Raw264.7(sh‐Calhm6). We observed that sh‐Calhm6 cells were resistant to IL‐4‐induced M2‐like polarization but spontaneously exhibited elevated expression of M1‐like macrophage markers. Furthermore, treatment with LPS/IFNγ further amplified the expression of these M1‐like markers (**Figure** **3**A). Microscopic analysis revealed that Calhm6‐deficient macrophages adopted a “fried egg” morphology with extended pseudopodia under LPS/IFNγ stimulation, whereas IL‐4 failed to induce the spindle‐shaped morphology characteristic of M2‐like macrophages (Figure 3B,C). Transcriptome analysis revealed that Calhm6 deficiency increased pro‐inflammatory gene expression and decreased anti‐inflammatory gene expression under both M1‐ and M2‐like skewing conditions (Figure 3D). Metabolic profiling of Calhm6 knockout BMDMs showed elevated extracellular acidification rates (ECAR) and reduced oxygen consumption rates (OCR), indicating a metabolic shift towards M1‐like glycolytic metabolism (Figure 3E,F). To assess whether Calhm6 deficiency drives M1‐like polarization in vivo, flow cytometry analysis demonstrated a higher percentage of CD86^+^ macrophages (M1‐like) among F4/80^+^ CD11b^+^ cells in the spleen and lymph nodes of Calhm6 knockout mice compared to wild‐type controls. Conversely, the proportion of CD206^+^ macrophages (M2‐like) was significantly reduced (Figure 3G,H). Notably, T and B cell development and activation in the thymus, spleen, and lymph nodes remained unaffected in Calhm6 knockout mice (Figure S3A–C, Supporting Information).

To elucidate the mechanism underlying this shift toward M1‐like polarization, we assessed the major signaling pathways involved in macrophage polarization. Notably, Calhm6 knockout BMDMs exhibited a marked decrease in the activation of Creb1 in response to LPS/IFNγ or IL‐4 stimulation (Figure 3I,J). Additionally, Calhm6 deficiency impaired the nuclear localization of Creb1 induced by CaCl2 and IL‐4 (Figure 3K). Pharmacological inhibition of Creb1 using KG‐501 and 666‐15 effectively suppressed Creb1 activation and M2‐like polarization in OE‐Calhm6 cells (Figure 3L,M). Furthermore, calcium influx induced by LPS was significantly reduced in Calhm6 knockout BMDMs (Figure 3N), highlighting the importance of Calhm6 in calcium‐mediated macrophage polarization. In addition, reduced calcium concentrations inhibited Creb1 phosphorylation in OE‐Calhm6 cells (Figure 3O).

Creb1 is a key transcription factor in M2‐like macrophage polarization. Activated Creb1 enhances the transcription of M2‐like markers directly or indirectly.^[^ ^23^, ^24^, ^25^, ^26^ ^]^ Conversely, activated Creb1 exerts an inhibitory effect on the expression of inflammatory cytokines. It does so by interfering with the interaction between RelA and CBP/p300. Consequently, when Creb1 is inactivated, macrophages tend to adopt a pro‐inflammatory phenotype by reducing the transcription of anti‐inflammatory cytokines and increasing the expression of pro‐inflammatory cytokines.^[^ ^27^, ^28^, ^29^ ^]^ Therefore, the activation of Creb1 correlates closely with the phenotypic switch of macrophages between M1‐like and M2‐like states caused by changes in Calhm6 expression. In conclusion, loss of Calhm6 enhances M1‐like macrophage polarization by inactivating Creb1, reinforcing the role of Calhm6 in maintaining macrophage homeostasis and suppressing inflammation.

### 2.4 Calhm6‐Deficient Mice Exhibit Enhanced Bactericidal Activity but Severe Inflammatory Responses

**Figure 4:**
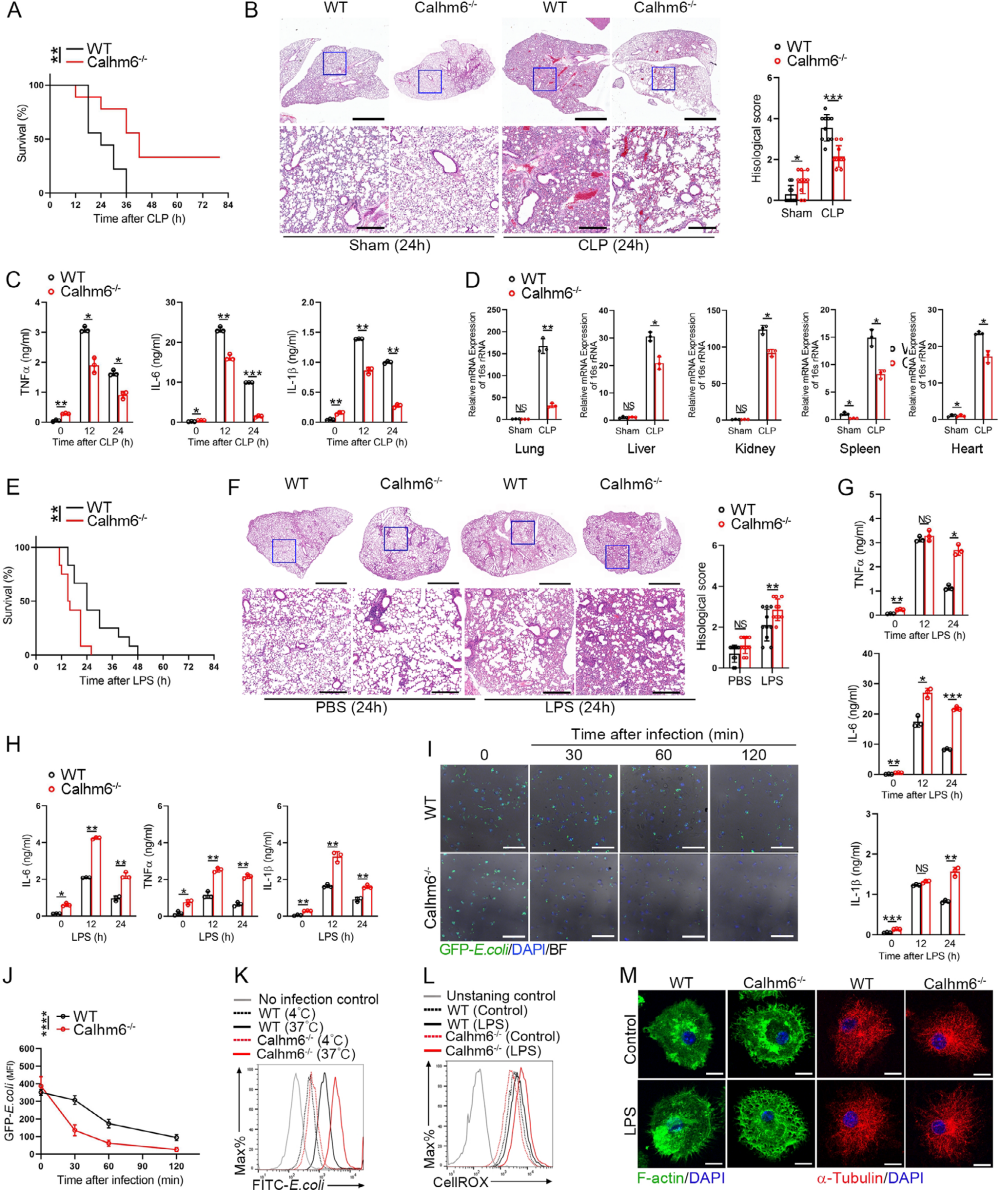
Calhm6‐deficient mice shows high bactericidal activity with severe inflammatory response and tissue damage. A–D) mortality (A), Hematoxylin‐and‐eosin (H&E) staining of inflammatory‐cell infiltration in the lungs (B), enzyme‐linked immunosorbent assay (ELISA) of serum cytokines (C), and bacterial load (as 16s rRNA) in the liver, kidney, lung, spleen and heart (D) of wild‐type or Calhm6 knockout mice (*n* = 9 per group per experiment) 24 h after sham treatment or sublethal CLP. Scale bars (B), 2 mm or 200 µm. Inflammation was determined by semiquantitative scoring. E,F) mortality (E), H&E staining of inflammatory‐cell infiltration and injury in the lungs (F), ELISA of serum cytokines G) of wild‐type or Calhm6 knockout mice (*n* = 12 per group per experiment) after 24 h of intraperitoneal injection of PBS or LPS (10 mg kg^−1^). Scale bars (F), 2 mm or 200 µm. Inflammation was determined by semiquantitative scoring. H) ELISA of IL‐6, TNFα, and IL‐1β in wild‐type and Calhm6 knockout BMDMs treated for 0, 12, or 24 h with LPS (200 ng mL^−1^). I,J) Fluorescence microscopy of wild‐type or Calhm6 knockout BMDMs infected for 0–120 min with GFP–*E. coli* (green) (MOI 20), then washed, fixed, and stained with the DNA‐binding dye DAPI (blue). Scale bars, 100 µm. Quantification of the results in (J), presented as mean fluorescence intensity (MFI). K) Flow cytometry of wild‐type or Calhm6 knockout BMDMs uninfected or infected for 20 min at 37 °C or 4 °C with FITC‐labeled *E. coli* (FITC‐*E. coli*) at an MOI of 100. L) Flow cytometry analyzing cellular ROS production of wild‐type or Calhm6 knockout BMDMs treated with LPS (200 ng mL^−1^) or PBS (control) for 24 h, followed by staining with CellROX (5 µm, 30 min). M) Confocal microscopy of wild‐type or Calhm6 knockout BMDMs treated with LPS (200 ng mL^−1^) or PBS (Ctr) for 30 min, then immunostained with anti‐F‐actin (green) or anti‐α‐Tubulin (red) and counterstained with DAPI (blue). Scale bars, 10 µm. The data represent the mean ± S.D. (*n* =3). NS, not significant (*p* > 0.05); *p < 0.05, **p < 0.01, and ***p < 0.001 compared with control, Student's *t*‐test.

Given the role of M1‐like macrophages in bacterial clearance, we investigated whether the M1‐like macrophages induced by Calhm6 deficiency display enhanced bactericidal activity. As anticipated, Calhm6 knockout mice displayed higher survival rates and significantly reduced pathological lung damage in the cecal ligation and puncture (CLP) model compared to wild‐type mice (**Figure** **4**A,B). Additionally, serum levels of inflammatory factors were significantly lower in Calhm6 knockout mice, and bacterial loads in tissues were reduced (Figure 4C,D). In contrast, intraperitoneal injection of ectosomal‐Calhm6 increased the mortality rate of wild‐type mice and the pathogenic load in various tissues (Figure S4A; Figure S4B, Supporting Information). However, in the LPS challenge model, Calhm6 knockout mice exhibited lower survival rates and more severe lung injury compared to wild‐type mice (Figure 4E,F). Pro‐inflammatory cytokine levels in the serum were markedly elevated (Figure 4G).

These contrasting outcomes between the CLP and LPS models suggest that the enhanced bactericidal activity of M1‐like macrophages in Calhm6 knockout mice suppresses cytokine storms and improves survival in the CLP model. In contrast, under LPS challenge, the excessive inflammatory response in Calhm6 knockout mice exacerbates tissue damage and mortality.^[^ ^30^, ^31^ ^]^ Consistent with this hypothesis, Calhm6 knockout BMDMs expressed significantly higher levels of IL‐6, TNFα, and IL‐1β upon LPS stimulation compared to wild‐type BMDMs (Figure 4H). Furthermore, Calhm6 knockout BMDMs exhibited enhanced bactericidal activity, characterized by increased phagocytosis and reactive oxygen species (ROS) production (Figure 4I–L). Conversely, OE‐Calhm6 cells displayed reduced phagocytic and bactericidal activity (Figure S4C,D, Supporting Information). Cytoskeletal remodeling is critical for macrophage phagocytosis of pathogens.^[^ ^32^ ^]^ Calhm6 knockout BMDMs displayed pronounced filopodia formation with tightly bundled actin and tubulin filaments compared to wild‐type BMDMs (Figure 4M). Additionally, the co‐localization of Rac1 with GFP‐labeled *E. coli* was significantly enhanced in Calhm6 knockout BMDMs (Figure S2E,F, Supporting Information). This finding aligns with previous reports that Rac1 plays a crucial role in macrophage bactericidal activity and cytoskeletal remodeling.^[^ ^7^, ^32^ ^]^

In summary, Calhm6 knockout mice exhibit enhanced bactericidal activity due to increased M1‐like macrophage polarization, leading to improved bacterial clearance and survival in the CLP model. However, this heightened activity also predisposes these mice to excessive inflammation and tissue damage in the LPS challenge model.

### 2.5 Calhm6 Functions as a Calcium‐Permeable Ion Channel in Macrophages

**Figure 5:**
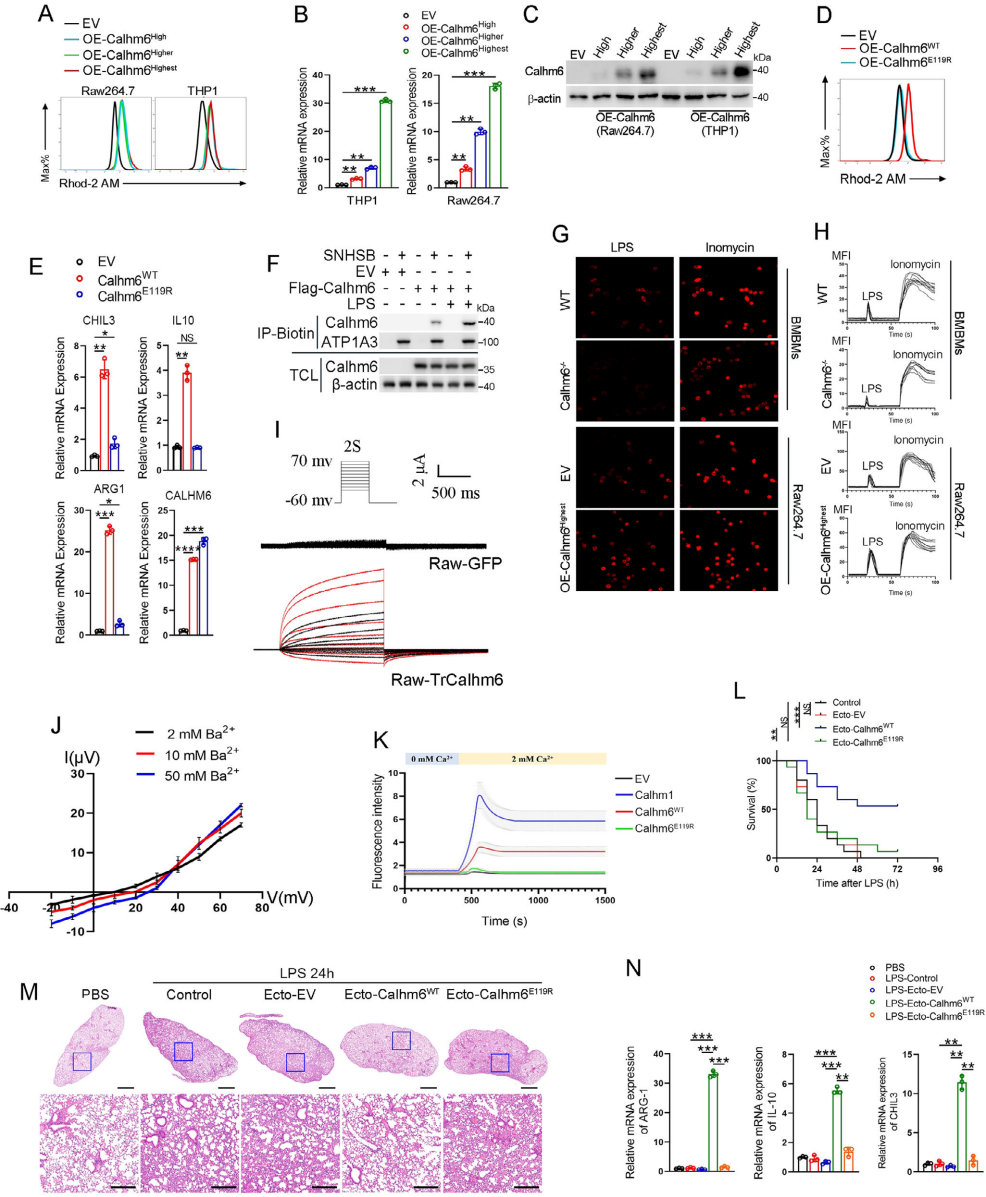
Calhm6 functions as a Ca^2+^ permeable ion channel in macrophages, mediating calcium influx upon LPS stimulation. A) Detection of calcium levels in macrophages (Raw264.7, THP1) with different Calhm6 expression levels (OE‐Calhm6^High^, OE‐Calhm6^Higher^, OE‐Calhm6^Highest^). Rhod2‐AM was used to label intracellular calcium ions. (B) and (C) Show the mRNA levels and protein expression levels corresponding to OE‐Calhm6^High^, OE‐Calhm6^Higher^, and OE‐Calhm6^Highest^, respectively. D) Comparison of calcium levels in EV, OE‐Calhm6^WT^, and OE‐Calhm6^E119R^ of Raw264.7, with Rhod2‐AM staining for calcium. E) mRNA levels of M2 polarization‐related indicators in EV, OE‐Calhm6^WT^, and OE‐Calhm6^E119R^ of Raw264.7. F) Western blot analysis of Calhm6 levels in cell surface proteins labeled with SNHSB (Sulfo‐NHS‐SS‐Biotin). OE‐Calhm6 cells were incubated with excess SNHSB (5 mm) at room temperature for 30 min, then washed three times with PBS containing 100 mm glycine to terminate the reaction. Cells were lysed with 1% Triton X‐100, and the lysate supernatant was incubated with Streptavidin Magnetic Beads at 4 °C for 6 h. Proteins bound to Streptavidin Magnetic Beads represent those localized on the cell surface. ATP1A3 served as a membrane protein marker. G) Confocal laser scanning was used to detect calcium concentration changes in different cells stimulated with LPS. Cells were stained with 4 µm rhod‐2AM. WT denotes wild‐type bone marrow‐derived macrophages (BMDM), Calhm6^−/−^ denotes Calhm6‐knockout BMDM, EV represents control Raw264.7 cells, and OE‐Calhm6 represents Raw264.7 cells overexpressing Calhm6. For each cell type, 100 consecutive images were captured at 2‐second intervals. Each experiment was independently repeated three times. In the LPS column, the fluorescence image shows the cell with the strongest fluorescence after LPS stimulation; in the ionomycin column, the image shows the cell with the strongest fluorescence after ionomycin stimulation. H) displays the time‐series trend of fluorescence values from individual cells (*n* = 6). I) Family of currents evoked in Raw264.7 cells transfected with pCMV‐GFP‐Calhm6(Raw‐TrCalhm6, *n *= 5) and Raw264.7 cells transfected with pCMV‐GFP (Raw‐GFP, *n *= 5) in response to voltage pulses from a holding potential of ‐60 mV in a bath solution (100 mm NaCl, 2 mm KCl, 2 mm CaCl_2_, 10 mm HEPES, pH 7.4). The top panel shows current traces recorded from Raw‐GFP, and the bottom panel shows current traces recorded from Raw‐TrCalhm6, where red represents LPS‐stimulated Raw‐TrCalhm6 (LPS 200 ng mL^−1^) and black represents non‐LPS‐treated Raw‐TrCalhm6. J) Current–voltage (I‐V) relations for Raw‐TrCalhm6 in bath solution containing different Ba^2+^ concentration (2 mm: 100 NaCl, 2 KCl, 2 BaCl_2_, 10 HEPES; 10 mm: 88 NaCl, 2 KCl, 10 BaCl_2_, 10 HEPES; 50 mm: 28 NaCl, 2 KCl, 50 BaCl_2_, 10 HEPES; pH 7.4. *n *= 4–6, SEM error bars). Whole‐cell currents were recorded using the same step voltage protocol as depicted in Figure 5I. K) Cytoplasmic Ca^2^⁺ measurements with Rhod‐2 AM loading and Ca^2^⁺ add‐back assays in Raw264.7 cells overexpressed with Flag‐Calhm1 (Calhm1), Flag‐Calhm6 (Calhm6^WT^), Flag‐Calhm6 ^E119R^ (Calhm6^E119R^), or control vector (EV). Cells were first incubated in Ca^2^⁺‐free buffer (0 mm CaCl_2_) and then challenged with 2 mm extracellular Ca^2^⁺ to monitor the progressive restoration of basal [Ca^2^⁺]i. Traces show the mean relative fluorescence units (RFU) ± SD (shaded areas) of five independent experiments. L) Survival analysis of septic mice induced by intraperitoneal LPS injection (10 mg kg^−1^). Mice were treated with Ecto‐Calhm6^WT^, Ecto‐Calhm6^E119R^, or Ecto‐EV (empty vector control) via intravenous administration 30 min after LPS challenge (*n* = 25 mice per treatment group). M) 12 h after ectosome treatment, hematoxylin and eosin (HE) staining of lung tissues from septic mice(LPS induced, corresponding to L). N) Effect of different ectosomes (labeled in the figure) on M2 polarization of bone marrow‐derived macrophages (BMDM). The data represent the mean ± S.D. (*n* =3). NS, not significant (*p* > 0.05); *p < 0.05, **p < 0.01,***p < 0.001 and ****p < 0.00001compared with control, Student's *t*‐test.

While we have observed that macrophages overexpressing Calhm6 exhibit elevated transient calcium influx upon LPS stimulation and that extracellular calcium concentration affects the activation of Creb1 in these cells, the calcium channel function of Calhm6 remains controversial.^[^ ^17^, ^20^, ^33^ ^]^ We found that macrophages overexpressing Calhm6 (Raw264.7^OE‐Calhm6^ and THP1^OE‐Calhm6^) displayed slightly elevated intracellular calcium levels. However, these levels did not correlate directly with Calhm6 expression (**Figure** **5**A–C). This discrepancy may stem from the limited localization of Calhm6 to the cell surface, with much of the expressed protein sequestered in intracellular compartments, such as the Golgi apparatus, which facilitates the secretion of Calhm6 in ectosomes. Consistently, cells expressing the highest level of Calhm6 secreted ectosomes containing more Calhm6 (Figure S5A, Supporting Information). Additionally, Glu119, a conserved residue analogous to Asp121 in Calhm1(Figure S5B, Supporting Information), is critical for Calhm6's calcium selectivity and permeability. Mutation of Glu119 to Arg (E119R) abolished Calhm6 ion currents in *Xenopus oocytes*.^[^ ^20^ ^]^ In macrophages overexpressing Calhm6E119R, we observed no significant increase in intracellular calcium and a failure to exhibit an M2‐like phenotype, further emphasizing the importance of calcium permeability in Calhm6‐mediated macrophage polarization (Figure 5D,E).

Given the potential for different subcellular localizations of Calhm6 in various cell types, including the Golgi apparatus, endoplasmic reticulum (ER), endosomes, and plasma membrane,^[^ ^20^, ^34^ ^]^ the direction of calcium transport by Calhm6 in macrophages remains unclear. We found that most Calhm6 in macrophages is localized intracellularly, but LPS stimulation promotes its translocation to the cell surface (Figure 5F; Figure S5C, Supporting Information). Glycosylation, especially O‐GlcNAcylation, is critical for membrane protein trafficking to the plasma membrane. LPS significantly enhanced Calhm6 glycosylation (Figure S5D, Supporting Information). Since E3 ubiquitin ligase RNF115 inhibits post‐ER trafficking by ubiquitinating RAB1A and RAB13,^[^ ^35^ ^]^ we tested its role in Calhm6 transport. RNF115 knockdown increased both Calhm6 glycosylation and membrane localization (Figure S5E,F, Supporting Information), indicating the LPS‐RNF115 axis is essential for Calhm6's response to LPS and cell membrane anchoring.

Existing literature indicates that plasma membrane‐localized Calhm6 mediates spontaneous calcium leakage,^[^ ^20^ ^]^ likely due to high permeability from its atypically large pore size.^[^ ^17^ ^]^ In agreement, we detected measurable calcium influx in Calhm6‐overexpressing RAW264.7 cells upon LPS stimulation (Figure 2J), confirmed by calcium imaging (Figure 5 G,H). In macrophages, intracellular calcium derives from: 1) extracellular influx through plasma membrane channels, including TRP channels and store‐operated calcium entry (SOCE); and 2) endoplasmic reticulum (ER) calcium release via inositol trisphosphate receptors (IP_3_R) or ryanodine receptors (RyR). Using specific inhibitors—HC‐067047 (TRP), BTP2 (SOCE), Xestospongin C (IP_3_R), and Dantrolene (RyR)—we found that inhibition of TRP, IP_3_R, or RyR did not alter LPS‐induced responses (Figure S5G, Supporting Information), while BTP2 modestly reduced them (Figure S5H, Supporting Information), implicating a minor SOCE contribution. Strikingly, Calhm6‐overexpressing macrophages maintained robust LPS‐induced influx despite SOCE blockade, highlighting Calhm6 as the dominant channel mediating this response.

To directly assess the channel properties of membrane‐localized Calhm6 in Raw264.7 cells, we transiently transfected Raw264.7 cells to express Calhm6 at a high level (Raw‐TrCalhm6) and recorded whole‐cell currents with or without LPS stimulation. LPS significantly enhanced ion currents in Raw‐TrCalhm6 compared to untreated controls (Figure 5I). The observed reversal potential near +10 mV indicates that Calhm6 functions as a non‐selective cation channel. To specifically evaluate Calhm6's permeability to Ca^2^⁺, we utilized Ba^2^⁺, which permeates similarly to Ca^2^⁺ but inhibits calcium‐dependent inactivation, allowing stable recordings. Stepwise increases in extracellular Ba^2^⁺ concentration induced progressive positive shifts in reversal potential (Figure 5J), consistent with Calhm6 permeability to divalent cations, including Ca^2^⁺.

Calhm channels are typically activated by extracellular calcium removal, inducing a pronounced “add‐back” calcium influx upon re‐addition.^[^ ^15^, ^16^, ^20^ ^]^ To determine if Calhm6 shares this property, we measured calcium signals in RAW264.7 cells overexpressing Calhm1, Calhm6, or Calhm6 ^E119R^ during calcium removal/re‐addition. Calcium re‐addition elicited robust influx in Calhm6 cells, but not with the fig5 E119R mutant (Figure 5K). Notably, despite comparable Calhm6 and Calhm1 protein/mRNA levels (Figure S5I,J, Supporting Information), Calhm6‐mediated influx was significantly lower than Calhm1's.

We next examined whether ectosomal Calhm6 mediates immunomodulation via its channel activity. In an LPS‐induced sepsis model, ectosomes enriched with wild‐type Calhm6 (Ecto‐Calhm6^WT^), but not loss‐of‐function Calhm6^E119R^ (Ecto‐Calhm6^E119R^) or empty‐vector control (Ecto‐EV), improved survival (Figure 5L), reduced lung pathology (Figure 5M), suppressed systemic IL‐6/TNF‐α/IL‐1β levels (Figure S5K, Supporting Information), and decreased organ injury markers including creatinine (CRE), blood urea nitrogen (BUN), alanine transaminase (ALT), and aspartate transaminase (AST) (Figure S5L, Supporting Information). In vitro, only Ecto‐Calhm6^WT^ promoted M2 polarization (Figure 5N), confirming that ectosomal Calhm6 exerts immunosuppressive effects through its calcium channel activity.

Previous studies report undetectable Calhm6 channel activity in N2a cells,^[^ ^20^, ^33^ ^]^ contrasting with robust activity in Calhm1/2.^[^ ^15^, ^16^, ^36^, ^37^ ^]^ Consistent with Danielli et al.,^[^ ^20^ ^]^ we attribute this absence to deficient membrane localization. HiS‐SIM (High Sensitivity Structured Illumination Microscopy) revealed prominent surface localization of Calhm1/2 in N2a cells, while Calhm6 was predominantly cytoplasmic (Figure S5M, Supporting Information). Flow cytometry of non‐permeabilized cells confirmed strong surface signals for Calhm1/2 but not Calhm6; permeabilization detected all three intracellularly (Figure S5N, Supporting Information). SNHSB surface biotinylation followed by streptavidin pulldown and western blotting further demonstrated Calhm1/2 in surface fractions, with Calhm6 absent (Figure S5O, Supporting Information). Quantification confirmed Calhm1/2 exhibits dual localization, whereas Calhm6 is exclusively cytoplasmic (Figure S5P–R, Supporting Information). These findings align with Malik et al.’s report of Calhm6's intracellular retention in ER/Golgi in 293T.^[^ ^34^ ^]^

In summary, this section confirms that Calhm6 functions as a calcium‐permeable ion channel in macrophages. Its calcium permeability, dependent on the conserved Glu119 residue, is crucial for M2‐like polarization. Calhm6 is primarily intracellular but translocates to the cell membrane upon LPS stimulation via glycosylation and RNF115‐mediated transport. It acts as a non‐selective cation channel mediating LPS‐induced calcium influx, with ectosomal Calhm6 exerting immunosuppressive effects through its channel activity. The lack of channel activity in certain cells is attributed to deficient membrane localization.

### 2.6 Calhm6 Promotes Creb1 Activation via the Chp1‐Camk4 Axis, Leading to M2‐Like Polarization of Macrophages

**Figure 6:**
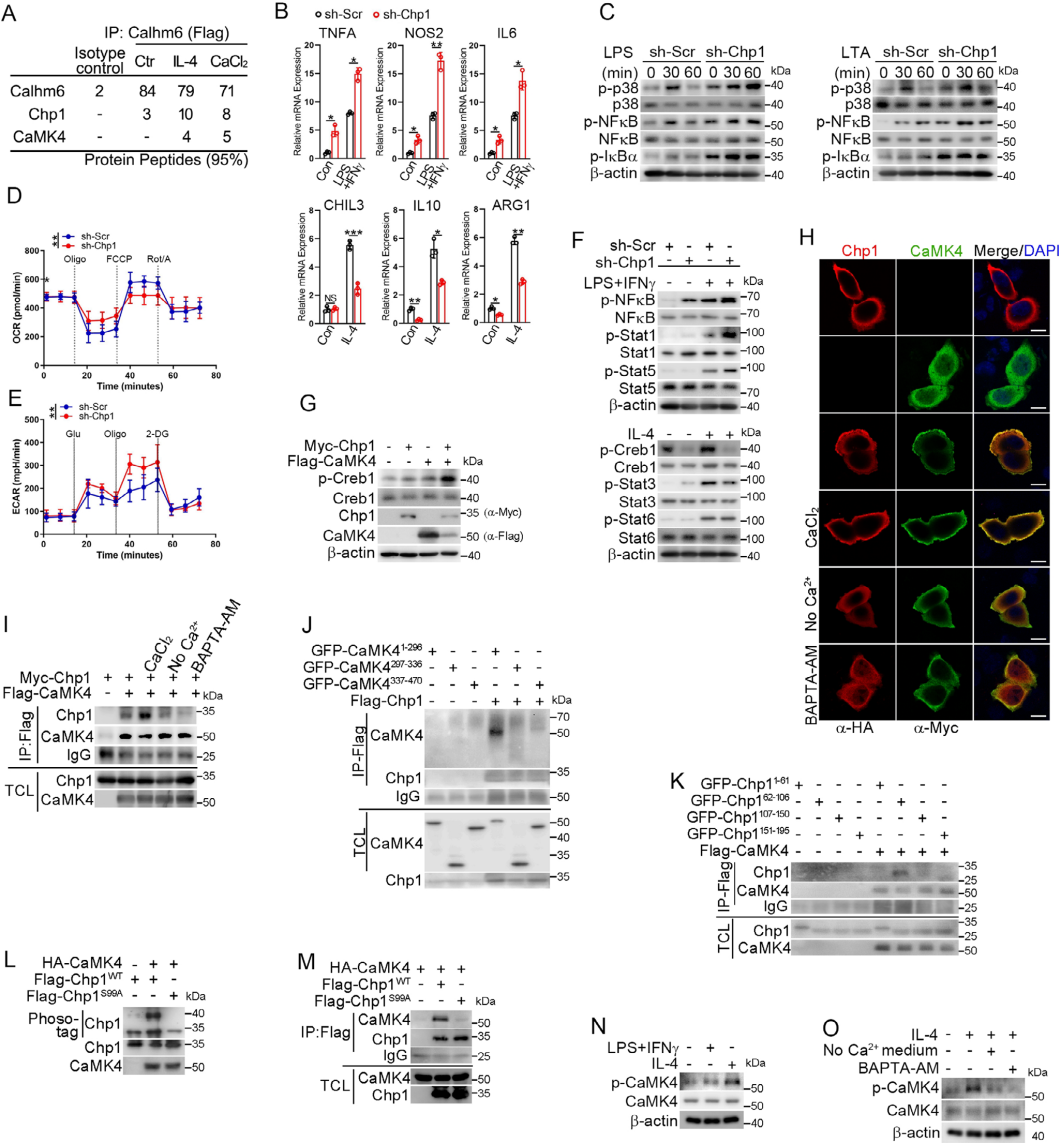
Calhm6 promotes Creb1 activation and M2‐like polarization of macrophages via the Chp1‐CaMK4 axis. A) Identification of Calhm6, Chp1, and CaMK4 by mass spectrometry in a Flag‐tagged Calhm6‐precipitation assay in cell lysates of OE‐Calhm6 cells, untreated or treated with IL‐4 (20 ng mL^−1^) or CaCl_2_ (2 mm). B) RT‐PCR of TNFA, NOS2, IL6, CHIL3, IL10, and ARG1 in Sh‐Scr or Sh‐Chp1 cells treated with LPS, IFNγ combined, or IL‐4 (dose as above). C) Immunoblot analysis of phosphorylated (p‐) p38, NFκB, IκBα, and β‐actin in total lysates of Raw264.7 cells treated with Scramble‐shRNA (Sh‐Scr) or Chp1‐specific shRNA (Sh‐Chp1), left treated for 0, 30, 60 min with LPS (200 ng mL^−1^) or LTA (100 ng mL^−1^). D,E) Seahorse analysis of OCR (D) and ECAR (E) in BMDMs from Sh‐Scr or Sh‐Chp1 cells. F) Immunoblot analysis of (p‐) Stat1, IκBα, Creb1, Stat3, Stat6, and β‐actin in Sh‐Scr or Sh‐Chp1 cells stimulated for 0, 30 min with LPS (200 ng mL^−1^) and IFNγ (10 ng mL^−1^) combined or IL‐4 (20 ng mL^−1^). G) Immunoblot analysis of p‐Creb1 and Chp1, CaMK4, β‐actin in Raw264.7 cells expressing Flag‐tagged CaMK4 and Myc‐tagged Chp1. H) HeLa cells were transfected with plasmids expressing Myc‐tagged CaMK4 and HA‐tagged Chp1. Before fixing the cells, they were treated with CaCl_2_, BAPTA‐AM, or Ca^2+^‐free DMEM for 3 h. Confocal microscopy shows the co‐localization of Chp1 (red) with CaMK4 (green) at the cell periphery. Images shown are representative of approximately 20 cells. Scale bar, 20 µm. I) Immunoassay of 293T cells expressing various combinations (above lanes) of Flag‐tagged CaMK4 and Myc‐tagged Chp1, and after being treated with PBS or the CaCl_2_, BAPTA‐AM, or Ca^2+^‐free DMEM for 3 h, assessed in anti‐Flag immunoprecipitates or total cell lysates, probed with various tag antibodies; below, immunoblot analysis of total cell lysates (TCL) without immunoprecipitation. J) Immunoassays of 293T cells expressing various combinations (above lanes) of Flag‐tagged Chp1 and the fragment of GFP‐tagged CaMK4 as indicated; immunoprecipitation with anti‐Flag and analysis by immunoblot with the tag antibodies; below, immunoblot analysis of total cell lysates (TCL) without immunoprecipitation. K) Immunoassays (as in J) of 293T cells expressing various combinations (above lanes) of Flag‐tagged CaMK4 and the fragment of GFP‐tagged Chp1 as indicated. L) Phos‐tag analysis of 293T cells expressing Flag‐tagged wild‐type Chp1 or Chp1 (S99A) and HA‐tagged CaMK4 (above lanes). M) Immunoassay of lysates of 293T cells expressing various combinations (above lanes) of Flag‐tagged wild‐type Chp1 or Chp1 (S99A) and HA‐tagged CaMK4, immunoprecipitated with anti‐Flag and analyzed by immunoblot with anti‐HA (α‐HA) or anti‐Flag (α‐Flag); below, immunoblot analysis of total cell lysates (TCL) without immunoprecipitation. N) Immunoblot analysis of (p‐) and total CaMK4 and β‐actin in BMDMs treated with LPS, IFNγ combined, or IL‐4 for 30 min. O) Immunoblot analysis of (p‐) and total CaMK4 and β‐actin in BMDMs treated with IL‐4 for 30 min after pretreatment with BAPTA‐AM (20 µm) or Ca^2+^‐free DMEM for 3 h. NS, not significant (*p* > 0.05); *p < 0.05, **p < 0.01, and ***p < 0.001 compared with control, Student's t‐test.

To investigate the mechanism by which Calhm6 activates Creb1, we treated OE‐Calhm6 cells with known inhibitors of Creb1 upstream kinase, including Go6983, Defactinib, KN62, FR180204, MK2206 and H‐89, which are inhibitors of focal adhesion kinase (FAK), protein kinase C (PKC), CaMKs, extracellular signal‐related kinases 1 and 2 (ERK1/2), Protein kinase B (AKT), and Protein kinase A (PKA) respectively. However, none of these inhibitors affected the expression of Creb1 target genes in OE‐Calhm6 cells (Figure S6A, Supporting Information). Next, we immunoprecipitated proteins from lysates of OE‐Calhm6 cells overexpressing Flag‐tagged Calhm6 using anti‐Flag beads and analyzed the immunoprecipitates by mass spectrometry. This revealed that IL‐4, a key inducer of M2‐like macrophage polarization, or CaCl_2_ facilitated the interaction between Calhm6 and two important calcium‐binding proteins, Chp1 and CaMK4 (**Figure** **6**A; Figure S6B, Supporting Information). These interactions suggest that Chp1 and CaMK4 may play critical roles in regulating macrophage polarization towards the M2‐like phenotype through Calhm6.

Similar to the phenotype observed in Calhm6‐knockdown macrophages, Chp1‐knockdown Raw264.7 cells (sh‐Chp1) exhibited a pro‐inflammatory M1‐like phenotype (Figure 6B; Figure S6C, Supporting Information). Additionally, sh‐Chp1 cells displayed elevated phosphorylation of mitogen‐activated protein kinase (p38), nuclear factor‐κB (NF‐κB), and IκBα upon stimulation with LPS or LTA (Figure 6C). Chp1 knockdown also resulted in reduced oxygen consumption rate (OCR) and elevated extracellular acidification rate (ECAR) in Raw264.7 cells (Figure 6D,E). Importantly, Creb1 activation was significantly diminished in sh‐Chp1 cells (Figure 6F), and the expression of Creb1 target genes was also reduced (Figure S6D, Supporting Information). Overexpression of Chp1 in Raw264.7 cells promoted their polarization towards the M2 phenotype (Figure S6E, Supporting Information) and increased Creb1 phosphorylation (to be illustrated in Figure 6G). These findings suggest that Chp1 may serve as a crucial mediator for Calhm6 to promote macrophage polarization towards the M2 phenotype by activating Creb1. However, since Chp1 lacks kinase activity, it theoretically cannot directly participate in the phosphorylation modification of Creb1.

Camk4 is another calcium‐related protein that we have identified to interact with calhm6 upon IL‐4 or CaCl2 induction. Notably, CaMK4 functions as a kinase, leading us to hypothesize that it serves as the kinase responsible for the phosphorylation of Creb1. Indeed, overexpression of CaMK4 promoted Creb1 phosphorylation. Notably, Chp1 enhanced this effect, and Chp1 alone also promoted Creb1 phosphorylation (Figure 6G). The role of Camk4 as an upstream kinase for Creb1 phosphorylation has also been confirmed in other studies.^[^ ^38^, ^39^ ^]^ However, the question remains: why does Chp1 alone promote the phosphorylation of Creb1? We speculate that the activation of Creb1 by Chp1 may be mediated through Camk4. Specifically, overexpressed Chp1 can utilize endogenous Camk4 to achieve the phosphorylation of Creb1.

To investigate the relationship between Chp1 and CaMK4, we observed that Chp1 and CaMK4 co‐localized at the cell membrane under both resting and CaCl_2_‐stimulated conditions. This interaction was suppressed in a calcium‐free medium or upon BAPTA‐AM treatment (Figure 6H), and this finding was confirmed by immunoprecipitation (Figure 6I). Furthermore, the kinase domain of CaMK4 (residues 1–296) and the EF‐hand 2 domain of Chp1 (residues 62–106) were essential for their interaction (Figure 6J,K,F, Supporting Information). Mass spectrometry and site‐directed mutagenesis revealed that CaMK4 phosphorylated Chp1 at Ser99, a site highly conserved across species (Figure 6L, Figure S6G–I, Supporting Information). Moreover, mutation of Chp1 at Ser99 (Chp1^S99A^) significantly disrupted its interaction with CaMK4(Figure 6M). Additionally, IL‐4 promoted the phosphorylation of CaMK4 in the presence of calcium, whereas LPS/IFNγ did not (Figure 6N,O), further supporting the role of CaMK4 in Calhm6‐mediated M2 polarization. These results suggest that there is an interaction between CaMK4 and Chp1 that is modulated by M2 polarization conditions or calcium. Moreover, this interaction participates in the phosphorylation modification of downstream Creb1, thereby regulating the polarization of macrophages towards the M2 phenotype.

### 2.7 Chp1 and CaMK4 Assemble with Calhm6 on the Membrane of Macrophages During M2‐Like Polarization

**Figure 7:**
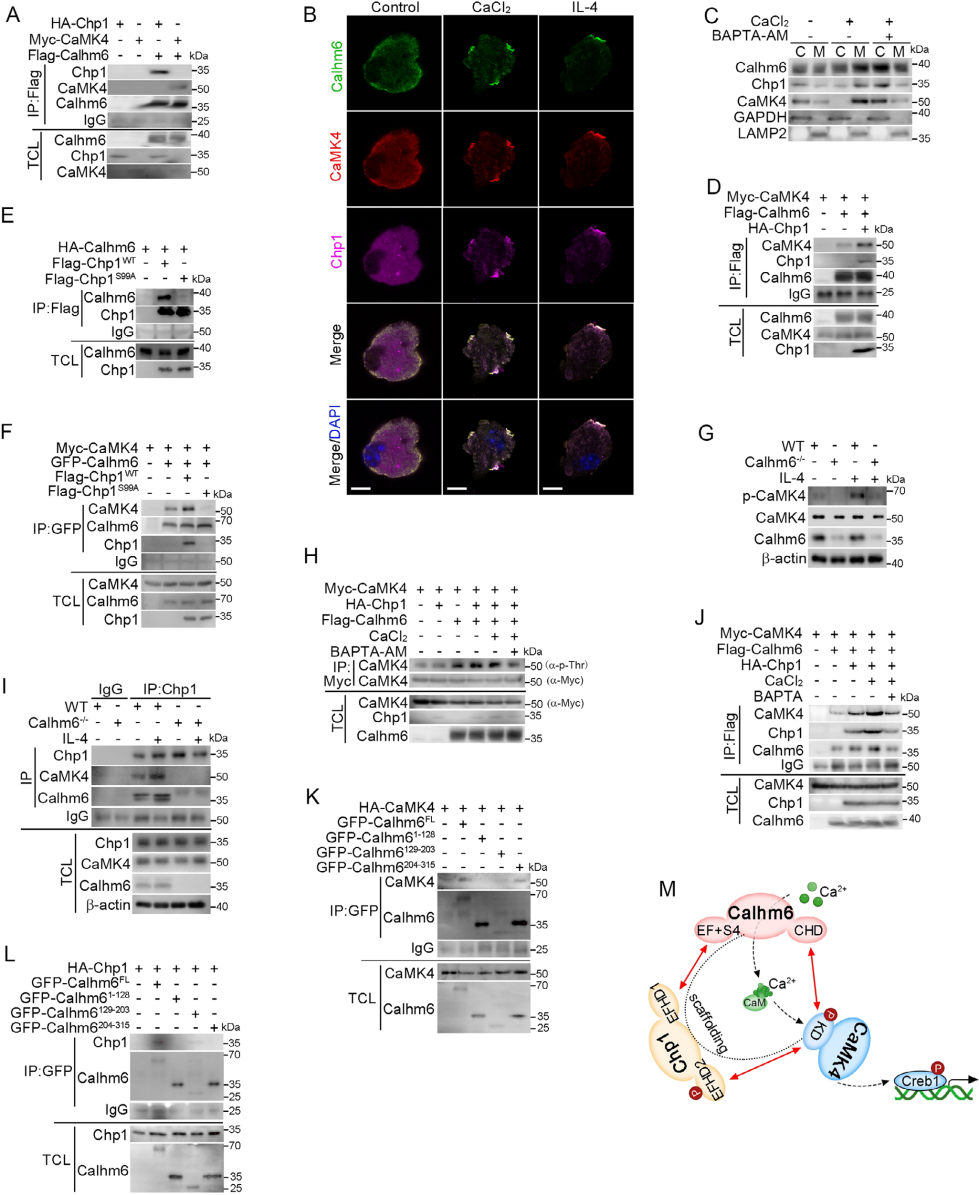
Chp1 and CaMK4 assemble with Calhm6 on the membrane of macrophages during M2‐like polarization. A) Immunoassay of 293T cells expressing various combinations (above lanes) of HA‐tagged Chp1, Myc‐tagged CaMK4, and Flag‐tagged Calhm6, assessed in lysates after immunoprecipitation with anti‐Flag (top group) or in total cell lysates without immunoprecipitation (below), probed with anti‐HA (α‐HA), anti‐Myc (α‐Myc), or anti‐Flag (α‐Flag). B) Fluorescence microscopy of the colocalization of Calhm6 (green), CaMK4 (red), and Chp1 (purple) in wild‐type BMDMs treated with or without CaCl_2_ (2 mm) or IL‐4 (20 ng mL^−1^) for 3 h. Scale bars, 20 µm. C) After 3 h of BAPTA‐AM (20 µm) pretreatment followed by CaCl_2_ stimulation for the same time, wild‐type BMDMs were separated into crude cytoplasm (C) and membrane (M) fractions. D) Immunoassay of 293T cells expressing various combinations of HA‐tagged Chp1, Myc‐tagged CaMK4, and Flag‐tagged Calhm6, assessed in lysates after immunoprecipitation with anti‐Flag or in total cell lysates without immunoprecipitation, probed with anti‐HA, anti‐Myc, or anti‐Flag. E) Immunoassay of lysates of 293T cells expressing various combinations (above lanes) of Flag‐tagged wild‐type Chp1 or Chp1 (S99A) and HA‐tagged Calhm6, immunoprecipitated with anti‐Flag and analyzed by immunoblot with anti‐HA (α‐HA) or anti‐Flag (α‐Flag); below, immunoblot analysis of total cell lysates (TCL) without immunoprecipitation. F) Immunoassay of lysates of 293T cells expressing various combinations (above lanes) of Myc‐tagged CaMK4, HA‐tagged Calhm6, and Flag‐tagged wild‐type Chp1 or Chp1 (S99A) and immunoprecipitated with anti‐HA and analyzed by immunoblot with anti‐HA (α‐HA), anti‐Myc (α‐Myc), or anti‐Flag (α‐Flag); below, immunoblot analysis of total cell lysates (TCL) without immunoprecipitation. G) Immunoblot analysis of (p‐) and total CaMK4, Calhm6, and β‐actin in wild‐type or Calhm6 knockout BMDMs treated with IL‐4 for 30 min. H) Immunoassay of 293T cells expressing various combinations (above lanes) of Myc‐tagged CaMK4, HA‐tagged Chp1, and Flag‐tagged Calhm6 after BAPTA‐AM pretreatment for 3 h followed by CaCl_2_ stimulation for the same time, assessed in anti‐Myc immunoprecipitates or total cell lysates, probed with tag antibodies; below, immunoblot analysis of total cell lysates (TCL) without immunoprecipitation. I) Immunoblot analysis of total lysates (bottom) and immunoprecipitates (top) of wild‐type or Calhm6 knockout BMDMs treated for 0 or 60 min with IL‐4. J) Immunoassay (as in H) of 293T cells expressing various combinations (above lanes) of Myc‐tagged CaMK4, HA‐tagged Chp1, and Flag‐tagged Calhm6 as indicated. K) Immunoassays of 293T cells expressing various combinations (above lanes) of Flag‐tagged CaMK4 and the fragment of GFP‐tagged Calhm6 as indicated; immunoprecipitation with anti‐GFP and analysis by immunoblot with the tag antibodies; below, immunoblot analysis of total cell lysates (TCL) without immunoprecipitation. L) Immunoassays (as in K) of 293T cells expressing various combinations (above lanes) of Flag‐tagged Chp1 and the fragment of GFP‐tagged Calhm6 as indicated. M) A proposed working model for the IL‐4 or calcium influx facilitating assembly of Chp1, CaMK4, and Calhm6, boosting activation of Creb1. The data represent the mean ± S.D. (*n *= 3).

We have clarified that the Chp1‐Camk4 axis serves as the mediator for Calhm6 to activate Creb1, as evidenced by a significant reduction in the expression of Creb1 target genes in Calhm6‐knockout BMDMs (Figure S7A, Supporting Information). However, the precise relationship between Calhm6 and the Chp1‐CaMK4 axis, particularly its role in Calhm6‐mediated M2‐like macrophage polarization, remained unclear. Here, we observed that both Chp1 and CaMK4 directly bind to Calhm6, with Chp1 showing a stronger binding affinity (**Figure** **7**A). Treatment with IL‐4 or CaCl_2_ promoted the assembly of CaMK4, Chp1, and Calhm6 on the macrophage membrane (Figure 7B). Membrane‐cytoplasm separation assays and confocal microscopy confirmed the membrane localization of the Calhm6‐Chp1‐CaMK4 complex (Figure 7C; Figure S7B, Supporting Information), suggesting that conditions inducing M2‐like polarization facilitate the assembly of this complex at the cell surface in a calcium‐dependent manner.

The affinity between CaMK4 and Calhm6 increased upon co‐expression with Chp1 (Figure 7D), indicating that Chp1 functions as a scaffold. Indeed, Chp1^S99A^ significantly reduced the interaction with Calhm6 and destroyed the assembly of Calhm6‐Chp1‐CaMK4 heterotrimer compared to the wild‐type Chp1 (Figure 7E,F). In addition, IL‐4 treatment robustly increased the CaMK4 phosphorylation only in the presence of calhm6 and calcium. (Figure 7G,H). Immunoprecipitation analysis revealed that the interaction between Chp1 and the complex could be disrupted by deletion of Calhm6 or treatment with the calcium chelator BAPTA‐AM (Figure 7I,J), indicating that the calcium concentration changes mediated by Calhm6 are involved in the regulation of macrophage polarization towards the M2‐Like phenotype and affect the assembly of the Calhm6‐Chp1‐Camk4 complex. Additionally, Calhm6 forms a polymer that displays ion channel functions.^[^ ^17^ ^]^ Co‐immunoprecipitation assays showed that co‐expression of Chp1 and CaMK4 enhanced the affinity between Calhm6 monomers (Figure S7C, Supporting Information). We also identified that the EF‐hand 1 domain of Chp1 (residues 1–61) interacted with the EF‐Hand/S4 domain of Calhm6 (residues 129–203), while the kinase domain of CaMK4 bound to the CHD domain of Calhm6 (residues 204–315) (Figure 7K,L; Figure S7D–F, Supporting Information). These data suggest that Creb1‐mediated M2‐like polarization of macrophages relies on Chp1 to maintain the affinity between Calhm6 and CaMK4, leading to the activation of Creb1 via CaMK4 (Figure 7M).

### 2.8 Irf1 and Stat6 Inversely Regulate the Calhm6 Expression During Macrophage Polarization

**Figure 8:**
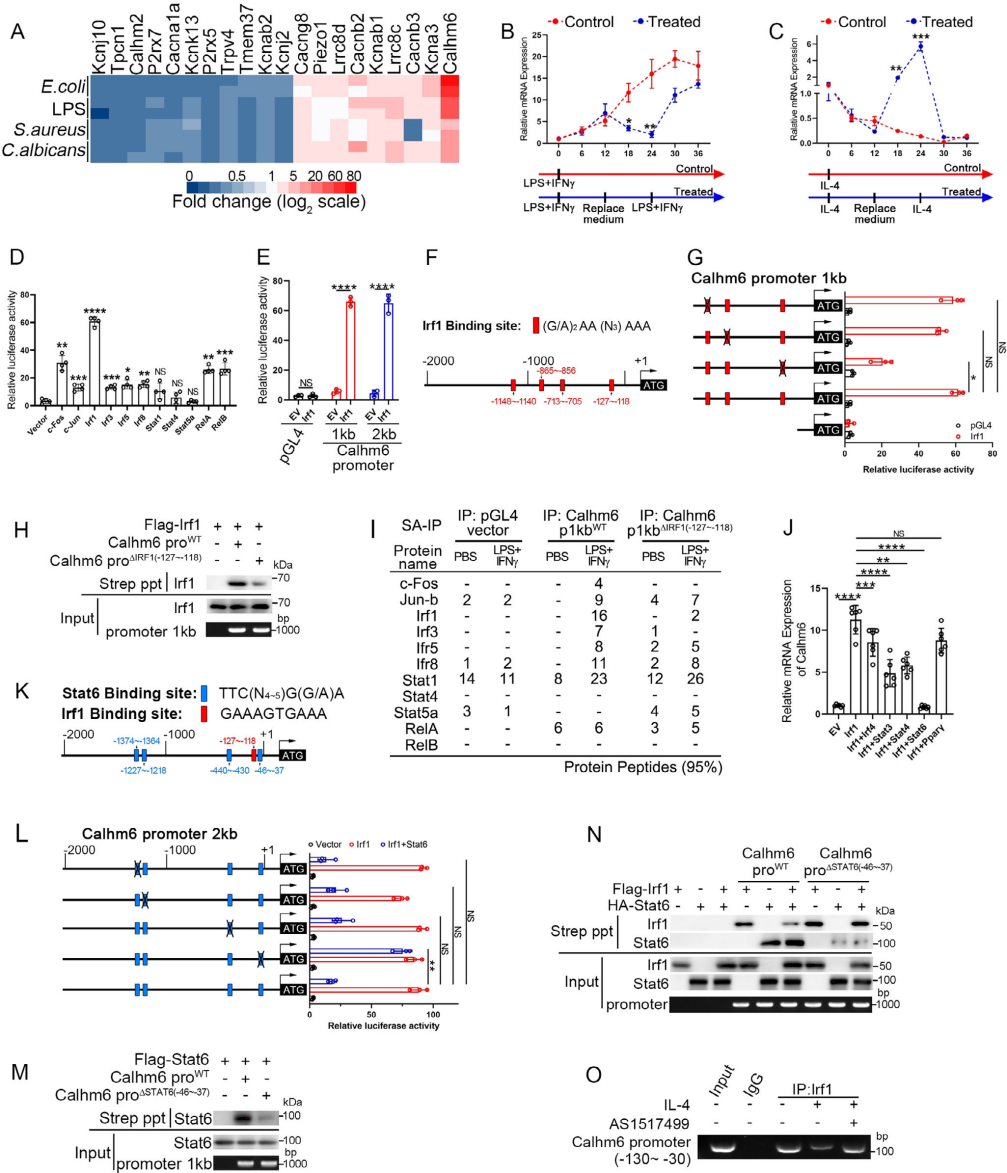
Irf1 enhances Calhm6 transcription in response to LPS/IFNγ activation but is blocked by IL‐4‐Stat6 axis. A) Heatmap of ion channels in DEGs between untreated BMDM and BMDM stimulated with LPS (200 ng mL^−1^), *E. coli*, *S. aureus* (MOI, 50), *C. albicans* (MOI, 30) for 12 h. B) After wild‐type BMDMs were treated with LPS (200 ng mL^−1^) and IFNγ (10 ng mL^−1^) combined for 12 h, the culture medium was replaced or without replacement, and LPS and IFNγ combined were treated again after 12 h until 36 h to harvest cells, and the expression of Calhm6 was analyzed by RT‐PCR. C) Wild‐type BMDMs were treated with IL‐4 (20 ng mL^−1^) for 12 h, then the culture medium was replaced or without replacement, and IL‐4 was treated again after 12 h until 36 h to harvest cells, and the expression of Calhm6 was analyzed by RT‐PCR. D) Luciferase reporter activity of 293T cells transfected with an empty luciferase reporter construct (pGL4‐basic) or a construct containing the 2‐kb Calhm6 promoter and main transcription factor of M1‐like phenotypes; and results are presented relative to those of cells transfected with the empty luciferase construct and empty retroviral vector. E) Luciferase activity (as in c) of 293T cells transfected with an empty luciferase reporter construct (pGL4‐basic) or a construct containing the 1‐kb or 2‐kb Calhm6 promoter (horizontal axis) and Irf1. F) Irf1‐binding sites in the Calhm6 promoter region; numbers above diagram indicate position relative to the transcription start site (ATG; far right), and those above (with vertical lines) demarcate the 1‐kb and 2‐kb promoter regions. G) Luciferase activity (assessed as in e) of 293T cells transfected with empty vector or plasmids expressing Irf1 and with a luciferase reporter containing the 1‐kb Calhm6 promoter (left margin) with or without various deletions (“X”). H) Streptavidin‐precipitation (Strep ppt) assay (top) of 293T cells expressing various combinations (above lanes) of Flag‐tagged Irf1, a biotinylated wild‐type or ΔIrf1 (−127 to −118) 1‐kb Calhm6 promoter, probed with anti‐Flag (α‐Flag); below, input of total chromatin fragments (1% agarose (bottom) indicates Calhm6 promoter DNA). I) Identification of indicated genes by mass spectrometry in streptavidin‐precipitation assays using biotinylated wild‐type or ΔIrf1 (−127 to −118) Calhm6 gene 1‐kb promoter (Calhm6 p1000) DNA fragments in BMDMs of wild‐type mice, followed by treatment with LPS and IFNγ combined for 12 h. J) RT‐PCR analysis of Calhm6 in 293T cells transfected with various combinations of an empty vector, Flag‐tagged Irf1, Irf4, Stat3, Stat4, Stat6, and Pparγ. K) Stat6‐ and Irf1‐binding sites (−127 to −118) in the Calhm6 promoter region (as in e); numbers above diagram indicate position relative to the transcription start site (ATG; far right), and those above (with vertical lines) demarcate the 1‐kb and 2‐kb promoter regions. L) Luciferase activity (as in G) of 293T cells transfected with empty vector or plasmids expressing Irf1 and Stat6 with a luciferase reporter containing the 2‐kb Calhm6 promoter (left margin) with or without various deletions (“X”). M) Streptavidin‐precipitation (Strep ppt) assay (as in H) of 293T cells expressing various combinations (above lanes) of Flag‐tagged Stat6, a biotinylated wild‐type or ΔStat6 (−46 to −37) 1‐kb Calhm6 promoter, probed with anti‐Flag (α‐Flag); below, input of total chromatin fragments (1% agarose (bottom) indicates Calhm6 promoter DNA). N) Streptavidin‐precipitation (Strep ppt) assay (as in H, M) of 293T cells expressing various combinations (above lanes) of Flag‐tagged Irf1 or HA‐tagged Stat6 and a biotinylated wild‐type or ΔStat6 (−46 to −37) 1‐kb Calhm6 promoter, probed with anti‐Flag (α‐Flag) or anti‐HA (α‐HA); below, input of total chromatin fragments (1% agarose (bottom) indicates Calhm6 promoter DNA). O) Chromatin immunoprecipitation of BMDMs under IL‐4 treatment for 12 h with the control antibody IgG or anti‐Irf1 after pretreatment with or without AS1517499 (3 µm) for 6 h followed by PCR with primers covering the Irf1‐ or Stat6‐binding sequences from position ‐130 to ‐30 of the Calhm6 promoter; Input (far left lane), PCR as above without immunoprecipitation. The data represent the mean ± S.D. (*n *= 3). NS, not significant (*p* > 0.05); *p < 0.05, **p < 0.01 and ***p < 0.001, ****p < 0.00001 compared with control, Student's *t*‐test.

While the phenotypic effects of Calhm6 deficiency and its downstream signaling pathways have been explored, the regulatory mechanisms underlying the intense expression of Calhm6 in macrophages in response to pathogens remain unclear. The heatmap analysis of RNA‐seq data, as depicted in Figure S1H (Supporting Information), revealed that Calhm6 exhibits the highest expression among ion channels in macrophages upon stimulation by pathogens such as *E. coli*, *S. aureus, C. albicans*, or LPS (**Figure** **8**A). We observed that Calhm6 mRNA levels increased after LPS/INFγ induction, decreased significantly after replacing the LPS/INFγ‐free medium at 12 h, and gradually rose again after LPS/INFγ rechallenge at 24 h (Figure 8B). In contrast, IL‐4 downregulated Calhm6 expression, which was reversed when the IL‐4‐free medium was applied at 12 h, followed by a decrease after subsequent IL‐4 stimulation at 24 h (Figure 8C).

Further analysis revealed that Irf1 is the primary transcription factor that significantly promotes Calhm6 transcription and expression (Figure 8D; Figure S8A, Supporting Information). Truncation analysis identified Irf1‐binding sites within the 1 kb region of the Calhm6 promoter (–1000 to +1) (Figure 8E). Within this region, three Irf1‐binding motifs ((G/A)AA(N3)AAA, where “(G/A)” denotes guanine or adenine, and “(N3)” represents any three nucleotides) were identified (Figure 8F). **Luciferase reporter** assays of the Calhm6 1‐kb promoter, with or without mutations in the Irf1‐binding sites, showed that the binding site between –127 and –118 bp was critical for Calhm6 transcription (Figure 8G). Streptavidin‐precipitation assays confirmed that Irf1 directly binds to the wild‐type 1‐kb Calhm6 promoter, with a reduced affinity observed for the mutant promoter (Figure 8H). Mass spectrometry analysis of biotin‐streptavidin pull‐down complexes revealed that, upon LPS/IFNγ treatment, transcription factors such as AP‐1 subunit c‐Fos (c‐FOS), Irf1, Irf3, and Stat1 exhibited increased affinity for the wild‐type promoter but reduced binding to the mutant promoter (Figure 8I). These findings suggest that Irf1 is the key transcription factor regulating Calhm6 expression during LPS/IFNγ signaling, and mutation of its binding site weakens its affinity and disrupts synergistic transcription with secondary factors like c‐Fos, Stat1, or Irf3.

Additionally, IL‐4 inhibits Calhm6 expression as mentioned before, and we sought to identify the transcription factors regulating this response by co‐expression with Irf1. Among several candidates, Stat6 emerged as the key inhibitor, significantly reducing Calhm6 promoter activity induced by Irf1 (Figure 8J; Figure S8B, Supporting Information). Four potential Stat6 binding sites were identified within the Calhm6 promoter (Figure 8K). Deletion of the –46 to –37 region notably abrogated Stat6‐mediated inhibition (Figure 8L). Further streptavidin pull‐down and mass spectrometry assays demonstrated that deletion of this region reduced Stat6's affinity for the promoter and weakened its competitive binding to Irf1 (Figure 8 M,N; Figure S8C, Supporting Information). Chromatin immunoprecipitation (ChIP) assays confirmed that Irf1 binds to the Calhm6 promoter in BMDM cells, but this binding is blocked by IL‐4. Interestingly, inhibition of Stat6 with AS1517499 restored Irf1 binding to the promoter (Figure 8O).

In conclusion, M1‐and M2‐like signals synergistically regulate Calhm6 expression via Irf1 and Stat6, maintaining a balance between anti‐infection and immune tolerance signals in vivo and preventing macrophages from overly differentiating to either phenotype.

## 3 Conclusion 

In summary, our findings demonstrate that CALHM6 plays a pivotal role in facilitating M2‐like macrophage polarization and maintaining immune homeostasis. Mechanistically, LPS/IFNγ stimulation triggers macrophages to upregulate CALHM6 transcription via IRF1, resulting in the enhanced secretion of CALHM6 within ectosomes that are internalized by neighboring macrophages. Furthermore, under conditions such as elevated calcium levels or IL4 stimulation, CALHM6, CHP1, and CAMK4 assemble on the cell membrane, initiating a signaling cascade through CHP1‐CAMK4 that promotes Creb1 phosphorylation and drives macrophage polarization toward an M2‐like phenotype. Additionally, IL4 enhances the binding affinity of STAT6 to the CALHM6 promoter, concurrently suppressing IRF1‐mediated transcriptional activation of CALHM6. These insights collectively highlight the intricate regulatory mechanisms governing macrophage plasticity and immune balance (Figure S9, Supporting Information).

## 4 Discussion

In this study, we have identified a novel role for Calhm6 in regulating macrophage polarization. As a calcium‐permeable channel, Calhm6 mediates calcium influx in macrophages, which is essential for the activation of downstream signaling pathways, including the Chp1‐CaMK4‐Creb1 axis. This calcium‐dependent mechanism drives M2‐like macrophage polarization, promoting anti‐inflammatory responses and tissue repair. Conversely, Calhm6 deficiency shifts macrophages toward an M1‐like phenotype, enhancing bactericidal activity but exacerbating inflammatory responses. This dual role underscores the versatility of Calhm6 in immune regulation, acting as a molecular switch that balances pro‐inflammatory and anti‐inflammatory signals depending on the cellular context. Additionally, the ability of Calhm6 to be secreted in ectosomes further highlights its unique functional characteristics. Ectosomal‐Calhm6 serves as a vehicle for intercellular communication, transferring its anti‐inflammatory effects to recipient cells. This mechanism not only amplifies the regulatory impact of Calhm6 but also provides a potential strategy for targeted therapeutic delivery. While we have demonstrated the role of ectosomal‐Calhm6 in immune modulation, the mechanisms underlying its packaging, release, and uptake by recipient cells remain poorly understood. Further studies on ectosome biogenesis and intercellular communication are needed.

Macrophages with high expression of Calhm6 showed relatively higher intracellular calcium concentration which significantly promoted the assembly of Calhm6‐Chp1‐Camk4, indicating that Calhm6 may mediate the flow of calcium along the concentration gradient, which is consistent with our observation that macrophages with high Calhm6 expression spontaneously display an M2‐like phenotype. We observed that only a small portion of Calhm6 in macrophages is localized on the cell surface, with the majority distributed within the cytoplasm, potentially primarily in the Golgi apparatus or endoplasmic reticulum,^[^ ^20^, ^34^ ^]^ which may be closely related to its secretion in the form of ectosomes. The translocation of Calhm6 to the cell surface appears to be a rapid stress response. We found that stimuli such as LPS, IL‐4, and Ca^2+^ can induce the translocation of Calhm6 to the cell surface, and there should be numerous other stressors with similar effects awaiting discovery.

The function of Calhm6 extends beyond the role traditionally attributed to ion channels in cellular signaling. Recent studies have established Calhm6 as an IRF3‐dependent NK‐activating molecule (INAM) expressed on activated macrophages and dendritic cells. This protein facilitates NK cell activation through synaptic communication, especially during Listeria monocytogenes infections, demonstrating its involvement in innate immune responses.^[^ ^20^, ^21^ ^]^ In contrast, our study emphasizes Calhm6's contribution to promoting an anti‐inflammatory M2 macrophage phenotype, which is crucial for resolving inflammation and facilitating tissue repair. Despite the differing immune contexts, both sets of studies underscore the central role of Calhm6 in immune cell communication and activation. Notably, our findings suggest that Calhm6's regulatory effects on macrophages not only help control the inflammatory environment but also contribute to immune homeostasis.

Moreover, the CALHM family has been suggested to be involved in purinergic signaling, and the finding that Calhm6 is involved in the release of ATP at the immunological synapse is consistent with the proposed role of the CALHM family in purinergic signaling.^[^ ^40^ ^]^ ATP release has been linked to autocrine and paracrine signaling, where extracellular ATP acts as a danger signal that can activate purinergic receptors, leading to inflammatory responses. This is particularly relevant in the context of pathogen recognition and the formation of inflammasomes, such as the NLRP3 inflammasome. Given that ATP release can trigger the assembly of the NLRP3 inflammasome, it is plausible that Calhm6 contributes to the regulation of inflammatory responses through its involvement in ATP secretion. The dual role of Calhm6 in promoting inflammation and anti‐inflammatory response emphasizes its versatility and complexity. Interestingly, CaMK4 controls the activation of the NLRP3 inflammasome in Type II alveolar epithelial cells during LPS‐induced ALI, and its inhibition could be a therapeutic approach for ALI.^[^ ^41^, ^42^ ^]^ As CaMK4 plays a crucial role in the Calhm6‐CaMK4‐Chp1 axis, we hypothesize that early infection triggers ATP release via Calhm6, which in turn contributes to inflammasome activation and host defense through CaMK4 regulation.

Despite the promising insights from our study, several critical questions remain to be addressed. First, although we have identified Calhm6's involvement in macrophage polarization, its tissue specificity and functional heterogeneity across different immune cell types remain unexplored. Macrophages from distinct tissues (e.g., lung versus liver) may exhibit varying responses to Calhm6 modulation. Understanding these tissue‐specific effects could provide valuable information for designing more targeted interventions in specific diseases. Second, while we identified the involvement of Irf1 and Stat6 in regulating Calhm6 expression, further research is needed to better understand how these transcription factors function in concert with each other. Irf1's activation of Calhm6 in response to LPS/IFNγ suggests that immune signals might regulate its expression in a highly dynamic manner. On the other hand, Stat6 acts as an inhibitor under IL‐4‐induced conditions, highlighting the complexity of immune regulation by Calhm6 and suggesting that fine‐tuning its expression could hold therapeutic promise. Additionally, exploring small‐molecule modulators of Calhm6 activity could lead to targeted therapies aimed at restoring immune homeostasis in diseases characterized by dysregulated inflammation. Ultimately, understanding the dual roles of Calhm6 in both pro‐inflammatory and anti‐inflammatory responses will be essential for harnessing its full therapeutic potential in diverse clinical settings.

## 5 Experimental Section

### Animals

The wild‐type and knockout mice were provided by Cyagn biotechnology Co., Ltd (Cyagn, CA, USA). All mice were maintained under specific pathogen‐free conditions at the Xi'an Jiaotong University Laboratory. Animal Care was performed in strict accordance with relevant guidelines and regulations required by the Animal Ethics Committee of Xi'an Jiaotong University (XJTUAE2023‐1303).

### Patients

Human blood specimens were obtained from 39 subjects with bacterial pneumonia and 47 healthy subjects at the Second Affiliated Hospital of Xi'an Jiaotong University. The clinical samples used in this study to identify the expression of CALHM6, IFNG, TNFA, and IL‐12 were supervised and granted by the Ethics Committee of the Second Affiliated Hospital of Xi'an Jiaotong University (2023‐052). Human CD11b^+^ macrophages were isolated with EasySep Human CD11b Positive Selection and Depletion Kit (StemCell Technologies). The characteristics of the healthy and bacterial pneumonia groups are presented in Table S1 (Supporting Information).

### Chemicals and Reagents

Construction of GFP‐expressing E. coli (GFP–*E. coli*) as described previously (26414765). The following reagents were used in this study: IFNγ (C746), IL‐4 (CK15), and m‐CSF (CB34) were from Novoprotein. DSS (D45600), NAC (N27390), and APAP (A32680) were from Acmec Biochemical. LPS (L5293) was from Sigma–Aldrich. LTA‐SA (tlrl‐slta) was from Invivogen. Calcium Colorimetric Assay Kit (S1063S), Lipofectamine 8000, and F‐actin tracker (C2201S) were from Beyotime Biotechnology. SYBR Green QPCR mix was from Vazyme. FSL‐1 TFA (HY‐P2036A), FITC (HY‐66019), Glutaraldehyde solution (HY‐Y1017A), FR180204 (HY‐12275), MK2206 (HY‐108232) and H‐89(HY‐15979A) were from MCE. Pam3csk4 (TP1067), BAPTA‐AM (T6245), Go6983 (T6313), Defactinib (T1996), KN62 (T2694), GSK3326595 (T5745), Hinokiflavone (T4S0181), Indisulam (T4321), AS1517499 (T4476), KG‐501 (T7297), 666‐15 (T5318) were from Tsbiochem. CellROX (C10422) and calcium‐free medium (21068028) were from Invitrogen. Biotin‐SS‐NHS‐Sulfo(P0663L) was from Beyotime.

### Cell Culture

The 293T and RAW264.7 cell lines were obtained from the National Collection of Authenticated Cell Cultures (Shanghai, China), tested for mycoplasma contamination, and were found to be negative, then were cultured in DMEM supplemented with 10% FBS and 1× penicillin–streptomycin (Invitrogen). For bone marrow‐derived macrophages (BMDMs), the femur and tibia were collected from mice of each genotype, and bone marrow cells were flushed with complete DMEM containing 50 mg mL^−1^ streptomycin and 10% FBS. Erythrocytes were removed via treatment with red blood cell lysis buffer, and the cell suspensions were filtered through a 40‐µm cell strainer for the removal of any cell clumps. The single‐cell suspensions were then cultured for 1 h at 37 °C, and non‐adherent cells were collected and re‐plated in complete DMEM with 25% medium conditioned by recombinant mouse M‐CSF (carrier‐free, 50 ng mL^−1^). For full differentiation of BMDMs, the cells were cultured for an additional 8 days with replacement of the medium every 2 days. All cells were labeled CD11b^+^ F4/80^+^ when analyzed by flow cytometry. For stable overexpression of Calhm6 cells (OE‐Calhm6), transfect 293T cells with the empty vector (EV) or Calhm6 overexpression plasmid along with packaging plasmids using a transfection reagent. Then, collect the lentivirus‐containing supernatant and transfect RAW264.7 cells in the presence of polybrene. After transfection, select the transfected cells using puromycin. Finally, validate the overexpression of Calhm6 in the resulting cells through qRT‐PCR or Western blot.

### Flow Cytometry Assay

Cells were isolated and purified from the spleen, lymph nodes, and thymus, and then stained with fluorescence‐conjugated antibodies for 15 min. The cells were resuspended in 1% BSA in PBS containing DAPI (Invitrogen, Carlsbad, CA, USA) after washing. Flow cytometry was performed using CytoFLEX LX (Beckman Coulter, CA, USA), and the data were plotted and quantified using FlowJo_v10.8.1 software. The fluorescence‐conjugated antibodies including anti‐CD45(103116), anti‐CD11b (101257), anti‐F4/80 (123108), anti‐CD3 (100204), anti‐CD4 (100438, 100412), anti‐CD8 (45‐0081‐82, 100708), anti‐CD86 (105012), anti‐CD206 (141716), anti‐CD62L (104412), anti‐CD44(103008) and anti‐B220 (103212). All antibodies were from eBioscience or Biolegend (San Diego, CA, USA).

### Measurement of ROS

Cells were treated with LPS (200 ng mL^−1^) for 1 h as needed. Then the culture medium was removed, and CellROX (C10422, Invitrogen) was incubated for 30 min at 37 °C after washing with PBS. Finally, washed the plates, collected cells with PBS containing 1 mm EDTA, and pelleted. Resuspended cells in cold PBS containing 1% FBS and analyzed by flow cytometry immediately.

### Probing Intracellular Calcium

Cells at amount of 3 × 10^6^ were washed with Hank's and stained with 4 µm Rhod2‐AM from Yeasen Biotechnology (40776ES72, Shanghai, China). The Rhod2‐AM working solution was removed after incubation at 37 °C for 30 min. Finally, the cells were digested and collected after PBS washing. The real‐time intracellular calcium was evaluated using CytoFLEX LX.

### Cecum Ligation Puncture (CLP) Model

Mice were anesthetized, and an abdominal incision was made for identification of the cecum. The distal one‐third of the cecum was ligated with a silk suture and was punctured once with a 22‐gauge needle. A small amount of cecal content was extruded through the perforation. The peritoneum and skin were closed with a continuous suture after the cecum was returned to the abdomen. For sham‐treated mice, all the same steps were performed, except for ligation and puncture of the cecum.

### LPS‐Induced Sepsis Model

Eight‐week‐old wild‐type and Calhm6^–/–^ mice were intraperitoneally injected LPS with 10 mg kg^−1^. After injection, survival rates were recorded. Serum and tissue samples were collected as required. Animals were obtained from the Cyagn biotechnology Co., Ltd. Lung tissues and serum used for histology or ELISA were harvested 24 h after LPS challenge.

### In Vivo Wound Healing Assay

Male C57BL/6J mice (8‐weeks‐old) were purchased from Cyagn biotechnology (CA, USA). After acclimatization for 7 days, the mice were anesthetized, and the hair on their dorsal surface was shaved. Two full‐thickness wounds were made to the back of each mouse using a 5‐mm biopsy punch as previously described.^[^ ^43^ ^]^ After creating the wounds, ring‐shaped silicone splints (inner diameter = 6 mm, outer diameter = 15 mm) fabricated from 0.5 mm‐thick silicone sheets (Grace Bio‐Labs, USA) were applied to the skin 1 mm away from the wound perimeter and were affixed with an instant‐bonding cyanoacrylate adhesive (Krazy Glue, USA). A 50 µL of PBS (control), ectosomal‐EV, or ectosomal‐Calhm6 (each *n* = 6) was applied to the wound area every two days for 10 days by parawound injection. The wounds were covered with a transparent film dressing (Tegaderm; 3 M Health Care, USA). The mice were sacrificed 10 days after injury; wound sites were digitally photographed, and wound areas were determined from the images using ImageJ v.1.53e software. Changes in wound area over time are expressed as the area of wound at time/ area of original wound×100%.

### APAP‐Induced Liver Injury (AILI) Model

Male C57BL/6J mice (8‐weeks‐old) were purchased from Cyagn biotechnology (CA, USA). C57BL/6J mice were administered acetaminophen (APAP) (Acmec Biochemical, China) via oral gavage after 12 h of starvation. APAP was used at a sublethal dose of 200 mg kg^−1^ for the liver injury study. The mice received i.p. injections of NAC (250 mg kg^−1^), ectosomal‐EV, ectosomal‐Calhm6, or PBS (vehicle control) (each *n* = 8) at the indicated times post‐APAP administration. For histopathology, the dissected liver tissues were fixed in buffered formalin, embedded in paraffin, and then processed for tissue‐section staining with H&E and TUNEL staining.

### The DSS‐Induced Colitis Model

Male C57BL/6J mice (8‐weeks‐old) were fed formulated drinking water containing 2.5% DSS for 3 days, and then i.p. injected with PBS, ectosomal‐EV, or ectosomal‐Calhm6 every other day while switched to regular drinking water until they were sacrificed. The mice were monitored for daily body weights, and the weights were recorded as a percentage of initial body weight. The disease activity index (DAI) was evaluated using a summed score of weight loss (score 0, 1, 2, 3, and 4), stool consistency (score 0, 1, 2, and 3), and bleeding (score 0, 1, 2, and 3). Mice were sacrificed at day 9, and tissue samples were collected for H&E staining.

### Seahorse Metabolic Analysis

After culturing for 8 days, BMDMs were seeded into XFe96 microplates and cultured overnight. The culture medium was switched to XF DMEM base medium (Cat No. 103575‐100, Agilent) supplemented with glucose (10 mm), pyruvate (1 mm), and glutamine (2 mm). The extracellular acidification rate (ECAR) and oxygen consumption rate (OCR) were measured using Seahorse XFp Glycolysis Stress Test Kit (No.103017‐100; Agilent Technologies) and Seahorse XFp Cell Mito Stress Test Kit (No. 103010‐100; Agilent Technologies), and performed using a Seahorse XF96 Extracellular Flux Analyzer instrument (Agilent Technologies, Santa Clara, CA) according to the manufacturer's instructions. The pretreated poly‐d‐lysine‐coated polystyrene Seahorse plate was incubated at 37 °C without CO_2_ for 45 min in medium with glucose (10 mm) and sodium pyruvate (1 mm). After baseline measurements, glucose (10 mm), oligomycin (1 µm), and 2 deoxy‐d‐glucose (2‐DG, 50 mm) were injected at the indicated time points, and ECAR (mpH min^−1^) was measured in real time. The oligomycin (1 µm), FCCP (1 µm), and rotenone/antimycin (0.5 µm) were used for OCR (pMoles O2 min^−1^) measurement. The data analysis was performed using Seahorse XFp Wave software (Wave 2.3.1).

### Measurement of Bacterial Loads in Tissues

An equal amount of tissue was aseptically obtained from the liver, kidney, lung, spleen, and heart. RT‐PCR analysis of bacterial load (as 16s rRNA) in the variation samples of the indicated mice at 24 h after sham treatment or sublethal CLP. The primer sequences of *16s rRNA* are presented in Table S2 (Supporting Information).

### Phagocytosis and Bacteria‐Killing Assay

For flow cytometry‐based measurement of phagocytosis, a total of 1 × 10^6^ BMDMs in PBS were cooled down to 4 °C for 30 min. Then, the cells were left uninfected or were infected for 20 min at 37 °C or 4 °C with heat‐killed, FITC‐labeled, and fresh mouse serum–opsonized *E. coli* (MOI, 100), after which they were washed extensively with cold PBS twice and fixed with 4% paraformaldehyde for 15 min. The fluorescence of extracellular particles was quenched by replacement of the medium with 0.2% Trypan blue in PBS, pH 5.5, shortly before the actual measurement by flow cytometry. For measurement of phagocytosis and killing via analysis of immunofluorescence, a total of 2 × 10^5^ BMDMs were grown on glass cover slips in six‐well plates. Cells were infected for 1 h with GFP–*E. coli* (MOI, 20), followed by several washes with PBS. Infected cells were further cultured in medium containing 10 µg mL^−1^ gentamicin. At the appropriate time points, the cells were washed in PBS and then fixed for 10 min at room temperature in 4% (vol/vol) paraformaldehyde. Slides were mounted with the mounting medium Vectashield (H1200; Vector Laboratories) containing DAPI and were imaged with a confocal microscope (LEICA SP8). The mean pixel intensity of GFP was measured with ImageJ v.1.53e software. The ratio of the total area of GFP dots to the overall cell number is plotted. For the in vitro bacteria‐killing assay, fresh overnight cultures of *E. coli* were suspended in PBS and opsonized with fresh mouse serum. The BMDMs were incubated for the appropriate time at 37 °C, with intermittent shaking, with *E. coli* (MOI, 20). After each time point, cells were lysed by the addition of distilled H_2_O, and diluted aliquots were spread on LB agar plates. CFU was assessed after incubation of the plates overnight at 37 °C.

### Lung Histology

Lung tissue was fixed in 4% paraformaldehyde (PFA) for 24 h and then dehydrated and embedded in paraffin. The tissue sections were stained with hematoxylin and eosin (H&E) stain. The histopathological score for each section was determined by semiquantitative scoring as described previously. In brief, inflammation was quantified by a treatment‐blind observer and graded on the following scale: 0, none; 1, mild; 2, moderate; 3, marked; and 4, severe. An increment of 0.5 was used when the inflammation was between two levels. The histopathological score of each section was the average of ten selected random fields.

### Transfection and Luciferase Reporter Assay

Cells cultured in 12‐well plates were transfected with the plasmids indicated in the main body of the text using Lipofectamine 8000 (Beyotime Biotechnology, China). At 48 h after transfection, cells were washed with PBS and lysed in Reporter Lysis Buffer (Invitrogen). Luciferase reporter activities were measured in triplicate using the Dual‐Luciferase reporter assay system (#RG027, Beyotime Biotechnology, China), according to the manufacturer's protocol, and quantified using a microtiter plate reader (FLUO star Omega, BMG LABTECH GmbH, Germany). The firefly luciferase to Renilla luciferase ratios were determined and defined as the relative luciferase activity.

### PAGEs and Immunoblotting

Gels for SDS‐PAGE or Phos‐tag SDS‐PAGE were prepared according to the manufacturer's instructions (NARD Institute). Proteins were separated by SDS‐PAGE or Phos‐tag SDS‐PAGE. Separated proteins were transferred onto a PVDF membrane and then were identified by immunoblot analysis with the appropriate primary antibodies at a dilution of 1:1000 (or as otherwise stated below). Antibody to phosphorylated (p‐) NFκB (AP0475), (p‐) IκBα (AP0731), (p‐) Stat1 (AP0054), (p‐) Stat3 (AP0070), (p‐) Stat6 (AP0456), (p‐) Creb1 (AP1421), (p‐) Stat5 (AP0758), anti‐CaMK4 (A5304), anti‐Chp1 (A15791), anti‐Mmp2 (A19080), anti‐CD63 (A5271), anti‐Arg1 (A1847) and anti‐Nos2 (A0312) were from Abclonal. Anti‐β‐Actin (66009‐1‐Ig), anti‐TGF‐beta1 (21898‐1‐AP), anti‐F4/80 (29414‐1‐AP) and antibody to phosphorylated p38 (28796‐1‐AP) were from Proteintech. Anti‐FAM26F (sc‐515780), Anti‐ HA (sc‐7392), anti‐HA (sc‐805), and anti‐Myc (sc‐40) were from Santa Cruz. Antibodies to anti‐Tsg101 (ET1701‐59) and Flotillin1 (ET7107‐82) were from HUABIO. Anti‐Flag (14793) was from Cell Signaling Technology. Anti‐GFP (ab290) and anti‐Tomm20 (ab186735) were from Abcam. Antibody to phosphorylated (p‐) CaMK4 (CPA4587) was from Cohesionbio. anti‐ATP1A3(10868‐1‐AP) and anti‐GAPDH(60004‐1‐Ig) was from Proteintech. Donkey anti‐Mouse IgG Highly Cross‐Adsorbed Secondary Antibody with Alexa Fluor™ 555 (A31570) was from Invitrogen. Horseradish peroxidase‐conjugated antibody to rabbit IgG (7074) or to mouse IgG (7076) (1:3000 dilution for each) was from Jackson ImmunoResearch Laboratories. The protein bands were visualized with a SuperFemto ECL Chemiluminescence Kit according to the manufacturer's instructions (E423‐01, Vazyme).

### Chromatin Immunoprecipitation (ChIP)

BMDMs under IL‐4 treatment for 12 h with the control antibody IgG or anti‐Irf1, after pretreated with or without AS1517499 (3 µm) for 6 h, and were stopped by 0.125 m glycine for 10 min at room temperature. The BMDMs were washed twice with cold PBS containing PMSF and harvested in SDS Lysis buffer from a ChIP Assay Kit (#P2078, Beyotime Biotechnology, China). Briefly, BMDMs were fixed with 1% formaldehyde for 10 min at room temperature (RT) and then were quenched with glycine (125 mm) for 5 min. The fixed cells were washed with PBS containing protease inhibitors and were lysed in lysis buffer (pH 8.1) containing 1% SDS, 10 mm EDTA, 50 mm Tris‐HCl, and protease inhibitors for 10 min on ice before the sonication, centrifugation, and addition of dilution buffer. 1% of input was removed, and the lysates were immunoprecipitated with 10 µL of anti‐Irf1 (#A7692, ABclonal) or 2 µL of Rabbit Control IgG (AC005, ABclonal) for 6 h. Salmon sperm DNA–protein A/G–Sepharose beads were added to the immunoprecipitation samples containing anti‐Irf1 or IgG (control) for incubation overnight. Immunocomplexes were washed sequentially with low‐salt buffer, high‐salt buffer, LiCl buffer, and twice with TE before elution in 200 mL of elution buffer (1% SDS, 0.1 m NaHCO_3_). The elutes were heated at 65 °C for 4 h to reverse the cross‐linking and were treated with RNase A for 30 min at 37 °C, followed by treatment with proteinase K for 1 h at 45 °C, to remove RNA and protein. DNA was recovered using a DNA Purification Kit (#D0033, Beyotime Biotechnology, China) and was eluted in 50 mL of QIAGEN EB buffer (QIAGEN, Hilden, Germany). 1% of input and 10% of the immunoprecipitates were used in PCR analyses using AmpliTaq Gold DNA polymerase (Applied Biosystems, CA, USA) for 30 cycles at 95 °C for 30 s, 58 °C for 30 s, 72 °C for 60 s (after an initial denaturation for 10 min at 95 °C). The primer sequences of −130 to −30 region on the promoter of Calhm6 are presented in Table S1 (Supporting Information).

### Confocal Fluorescence Microscopy

For the frozen section of skin, the sections were air‐dried overnight and fixed in ice‐cold acetone for 10 min, air‐dried briefly, and blocked with 5% normal horse serum for 20 min. Then incubated overnight at 4 °C with the primary antibodies, including anti‐F4/80 (1:100 dilution, Proteintech, 29414‐1‐AP) and anti‐CD206 (1:100 dilution, Proteintech, 60143‐1‐Ig). After three washes with PBS, the sections were incubated for another 1 h with secondary antibodies (1:250, Alexa Fluor 555‐conjugated anti‐Rabbit IgG (A31572) or Alexa Fluor 488‐conjugated anti‐mouse IgG (A21202); all from Invitrogen). Subsequently, the sections were washed three times with PBS and were mounted with Vectashield mounting medium containing DAPI (Vector Labs). All images were collected with a confocal microscope (LEICA SP8). For cultured cells, BMDMs seeded on glass coverslips in six‐well dishes were stimulated with DMSO, GFP–*E. coli*, CaCl_2_, LPS, IFNγ combined, IL‐4, calcium‐free medium or BAPTA‐AM for indecated time as needed. HeLa cells seeded on coverslips in six‐well plates with 30% confluence were transfected with the appropriate constructs and were cultured for another 24 h. The cells were washed three times with PBS and were fixed for 15 min at room temperature with 4% (vol/vol) paraformaldehyde, after which additional immunofluorescence staining was applied. For staining with anti‐FAM26F (1:100 dilution, Santa cruz, sc‐515780), Rac1 (1:100 dilution, Proteintech, 24072‐1‐AP), anti‐Creb1 (1:100 dilution, Proteintech, 12208‐1‐AP), anti‐alpha‐tubulin (1:100 dilution; HUABIO, ER130905), anti‐Tsg101 (1:100 dilution; HUABIO, ET1701‐59), anti‐Chp1 (1:100 dilution, Abclonal, A15791), anti‐CaMK4 (1:100 dilution, Abclonal, A5304), anti‐Myc tag (1:500 dilution, Santa Cruz, sc‐40) or anti‐HA tag (1:500 dilution, Santa Cruz, sc‐805), anti‐Flag tag (1:500 dilution, CST, 14793) fixed cells were rinsed with PBS and then were incubated for 10 min on ice with 0.2% Triton X‐100 and 0.2% bovine serum albumin (BSA) in PBS. Following permeabilization, nonspecific binding in the cells was blocked by incubation for 1 h at room temperature with 0.02% Triton X‐100 and 5% BSA in PBS, and cells were incubated for 1 h with specific primary antibodies (identified above). After three washes with PBS, the cells were incubated for another 1 h with secondary antibodies Alexa Fluor 555‐conjugated anti‐rabbit IgG (A31572), Alexa Fluor 647‐conjugated anti–rabbit IgG (A21246), or Alexa Fluor 488‐conjugated anti‐mouse IgG (A21202) (all from Invitrogen). Subsequently, the cells were washed three times with PBS and were mounted with Vectashield mounting medium containing DAPI (Vector Labs). All images were collected with a confocal microscope (LEICA SP8).

### Preparation of Exosomes and Ectosomes

Exosomes and ectosomes were isolated by differential centrifugation processes. Empty vector (EV) and overexpression of (OE‐) Calhm6 cells constructed based on Raw264.7 cells, or wild‐type and Calhm6 knockout BMDMs (each 5 × 10^6^) were cultured in DMEM medium containing 10% exosome‐depleted FBS for 24 h. Culture medium was first collected and centrifuged (300 ×g, 10 min, 4 °C) to remove cells. The supernatant was then centrifuged (2000 ×g, 20 min, 4 °C) and filtered through 0.8 µm pore size filters (Millipore) to remove cell debris. The collected medium was centrifuged again (16500 ×g, 30 min, 4 °C) to obtain pellets containing the ectosomes. The remaining supernatant was finally employed to isolate exosomes by ultracentrifugation (100000 ×g, 90 min, 4 °C). The obtained exosomes and ectosome pellets were washed once with phosphate‐buffered saline (PBS) and resuspended in PBS for 100 µL. The size and homogeneity of exosomes and ectosomes were examined by particle size analyzer (NanoFCM, N30E), and the images were collected under a transmission electron microscope (H‐7650 Hitachi microscope; Hitachi, Tokyo, Japan). The exosomes or ectosomes proteins were further detected by western blot, Immunofluorescence, and mass spectrometry. Ectosome isolation from plasma was performed as previously described.^[^ ^44^ ^]^ Briefly, EDTA‐anticoagulated blood was centrifuged at 1200 × g for 15 min at room temperature to separate plasma from remaining blood cells. The plasma samples were then centrifuged at 1500 ×g for 15 min at room temperature to pellet larger cell debris and remove remaining platelets. Plasma ectosomes were finally isolated by centrifuging at 14000 × g for 35 min at 4 °C and washed once with PBS.

### Cytokine Measurement

The concentration of inflammation‐related indicators in the serum was detected using ELISA assay kits (Invitrogen), including interleukin 1beta (IL‐1β) (Cat # 88‐7013‐77, Invitrogen), IL‐6 (Cat # 88‐7064‐77), tumor necrosis factor alpha (TNFα) (Cat # 88‐7324‐77, Invitrogen), and interleukin‐10 (IL‐10) (Cat # 88‐7105‐77, Invitrogen). The antibody was coated on the bottom of the culture plate before standards and samples were added to the wells. Then, the target antibody and HRP‐conjugated secondary antibody were incubated according to the instructions. Added the termination solution after washing and measured at 450 nm with a microtiter plate reader (FLUO star Omega, BMG LABTECH GmbH, Germany).

### shRNA and Lentiviral Infection

Lentivirus was produced by co‐transfection of 293T cells with shRNA in the vector pll3.7, PMDLg/RRE, pRSV‐Rev, and pVSV‐G plasmids using Lipofectamine 2000 (Invitrogen). The shRNA sequences of ‐ Chp1 are presented in Table S2 (Supporting Information). The most efficient shRNA was Chp1‐specific shRNA‐1. Viral supernatants were harvested at 48–72 h after transfection, then were passed through a 0.45‐µm filter, diluted 2:3 with fresh medium containing 8 µg mL^−1^ polybrene, and used to infect the target cells at 80% confluence. Protein expression was visualized by immunoblot, RT‐PCR, and Seahorse analysis.

### Quantitative Real‐Time PCR

Total RNA was extracted and quantified, and cDNA was synthesized from 1 µg total RNA using 5X All‐In‐One RT Mastermix (Applied Biological Materials, Cat. No. G492) in an amount of 2 µg total RNA. The qRT‐PCR was performed using FTC‐3000P Real‐Time Quantitative System (Funglyn Biotech, Canada) for mRNA analysis. The primer sequences are presented in Table S2 (Supporting Information).

### RNA‐seq Analysis

The *Escherichia coli* (*E. coli*), *Staphylococcus aureus* (*S. aureus*), and *Candida albicans* (*C. albicans*) were obtained from the China General Microbiological Culture Collection Center (CGMCC) (Beijing, China). Bacteria were cultured overnight in the indicated medium and washed twice with PBS. Followed by treatment with LPS (10 mg kg^−1^) or infected with *E. coli* (MOI, 50), *S. aureus* (MOI, 50), *C. albicans* (MOI, 30) for wild‐type BMDMs at 12 h. The sample RNA was extracted and prepared using RNAsample Total RNA kit method, and the quality of RNA was evaluated. A RNA second‐generation DNA library was established, and transcriptome sequencing and bioinformatics analysis were performed by Oebiotech (Shanghai, China).

### Mass Spectrometry

After staining of gels with Coomassie blue, excised gel segments were subjected to in‐gel trypsin digestion and dried. Peptides were dissolved in 10 µL 0.1% formic acid and were auto‐sampled directly onto a 100 µm × 10 cm fused silica emitter made in‐house and packed with reversed‐phase ReproSil‐PurC18‐AQ resin (3 µm and 120 Å; Ammerbuch). Samples were then eluted for 60 min with linear gradients of 5–32% acetonitrile in 0.1% formic acid at a flow rate of 300 nL min^−1^. Mass spectra data were acquired with a TripleTOF 5600+ mass spectrometer (AB Sciex) equipped with a nano‐electrospray ion source. Data were collected in an IDA mode. The wiff files were searched with the ProteinPilot (Version 4.5) against a database from the Uniprot protein sequence database.

### Colocalization Analysis

For colocalization analysis, images were taken with a ×63 or ×100 oil objective, and the analysis was done with ImageJ software. The autothreshold function was used to process and analyze the colocalized pixels. Pearson's correlation coefficient (Rr) was used to measure the degree of colocalization, with 8–12 images/group.

### Quantification and Statistical Analysis

All data are representative of at least three independent experiments. All statistical analyses were performed using Prism8 (GraphPad). The liver TUNEL labeling count, relative mRNA levels, bacteria number, length of cell, MFI, Relative Luciferase activity, and ELISA were tabulated graphically with error bars corresponding to means ± SD and compared using two‐tailed Student’ s *t*‐test. The data are presented as the mean ± SD as indicated in the legends. The two‐way ANOVA analysis was used for comparing serum calcium, Seahorse, DIA, body weight, and wound area. Survival data were analyzed by the Kaplan–Meier statistical method. The relative expression levels of CALHM6, IFNG, TNFA, and IL‐12 in CD11b^+^ macrophages isolated from the blood of patients were plotted, and the linear regression t‐test was applied. A *p*‐value < 0.05 was considered statistically significant.

### Raw264.7 Cell Electrophysiology

Whole‐cell patch‐clamp recordings were performed on transfected Raw264.7 cells. The experimental group consisted of cells transfected with pCMV‐GFP‐Calhm6(constructed in‐house, a bicistronic vector allowing for separate expression of Calhm6 and GFP), which allows for the identification of successfully transfected cells via independent GFP expression, while cells transfected with pCMV‐GFP served as a control. Cells were identified based on GFP fluorescence using an Olympus IX73 microscope. Recording electrodes were positioned onto the cell membrane using a micromanipulator. A giga‐ohm seal was established by applying gentle negative pressure, followed by membrane rupture to achieve the whole‐cell configuration. A patch‐clamp amplifier was used to compensate for fast and slow capacitances and series resistance (compensated to 70–80%). Currents were elicited using a step‐pulse protocol from a holding potential of −60 mV, with test potentials ranging from −20 mV to +70 mV in 10 mV increments, each applied for 2 s. All recordings were carried out at 25 °C under continuous oxygenation. The external bath solution contained 100 mm NaCl, 2 mm KCl, 2 mm CaCl_2_, and 10 mm HEPES (pH 7.4), though minor adjustments were made in certain experiments.

### Visualization and Localization of Calhm1/2/6 via Immunofluorescence and HiS‐SIM

N2a cells in the logarithmic growth phase were seeded into confocal dishes and allowed to adhere until reaching 60–70% confluence. The cells were fixed with 4% paraformaldehyde for 15 min, washed with PBS, and permeabilized with 0.1% Triton X‐100 for 10 min to enhance membrane permeability. Subsequently, blocking was performed with 2.5% BSA at room temperature for 1 h. A mouse anti‐HA primary antibody was applied and incubated overnight at 4 °C. After thorough washing with PBS, an Alexa Fluor 568‐labeled goat anti‐mouse IgG secondary antibody (Invitrogen, #A31570) was incubated at room temperature for 1 h protected from light. Finally, the nuclei were counterstained with DAPI, and the samples were mounted with an anti‐fade mounting medium. The prepared specimens were imaged using a HiS‐SIM super‐resolution microscope. The subcellular localization of the target protein (cytoplasm or cell membrane) was determined by analyzing the spatial relationship between the red fluorescent signal (target protein) and the cell outline, as well as the blue fluorescent signal (nuclei).

## Conflict of Interest

The authors declare no conflict of interest.

## Author Contributions

J.G. and Y.X. conceptualized the study. Y.X., X.X., Y.Z., S(i)Z., and W.L. developed the methodology. Y.X., Y.Z., S(h).Z., Y.Y., X.C., W.L., and I.H. performed the investigation. J.G., Y.X., S(i).Z., Y.L., L.Z., and H.L. visualized the study. J.G., R.Z., and Z.L. acquired funds. Project administration: J.G., Z.L., and H.L. performed project administration. J.G. performed supervision. J.G., Y.X., and X.X. wrote the original draft.

## Supporting information



Supporting Information

Supporting Information

Supporting Information

Supporting Information

Supporting Information

Supporting Information

Supporting Information

Supporting Information

Supporting Information

Supporting Information

Supporting Information

## Data Availability

The data that support the findings of this study are openly available in GEO Accession at https://www.gsat.us/search/node/GSE249905;https://www.gsat.us/search/node/GSE249904, reference number 249905.
