# Correction to “Calhm6 Governs Macrophage Polarization Through Chp1‐Camk4‐Creb1 Axis and Ectosomal Delivery in Inflammatory Responses”

**DOI:** 10.1002/advs.76458

**Published:** 2026-07-20

Y. Xin, X. Xiong, Y. Zhang, et al. “Calhm6 Governs Macrophage Polarization Through Chp1‐Camk4‐Creb1 Axis and Ectosomal Delivery in Inflammatory Responses.” *Advanced Science* 13, no. 1 (2026): e02395. https://doi.org/10.1002/advs.202502395.

**Correction 1**

In Figure 1, the Flotillin1 band in Panel F is correct. Only the β‐actin band in Panel H was pasted incorrectly during figure assembly. The corrected Figure 1 is provided below.


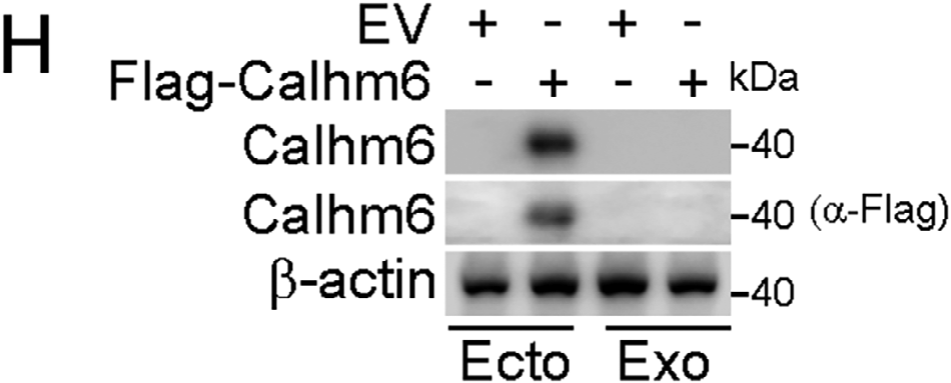


Corrected Figure 1H

**Correction 2**

After verifying the original files, we confirm that Figure 6M is fully correct and requires no changes. The error is limited to Figure 7E, where the IP‐Chp1 and TCL‐Chp1 bands were mistakenly replaced by the Chp1 bands from Figure 6M. We have updated Figure 7E using authentic raw data. The corrected Figure 7E is provided below.


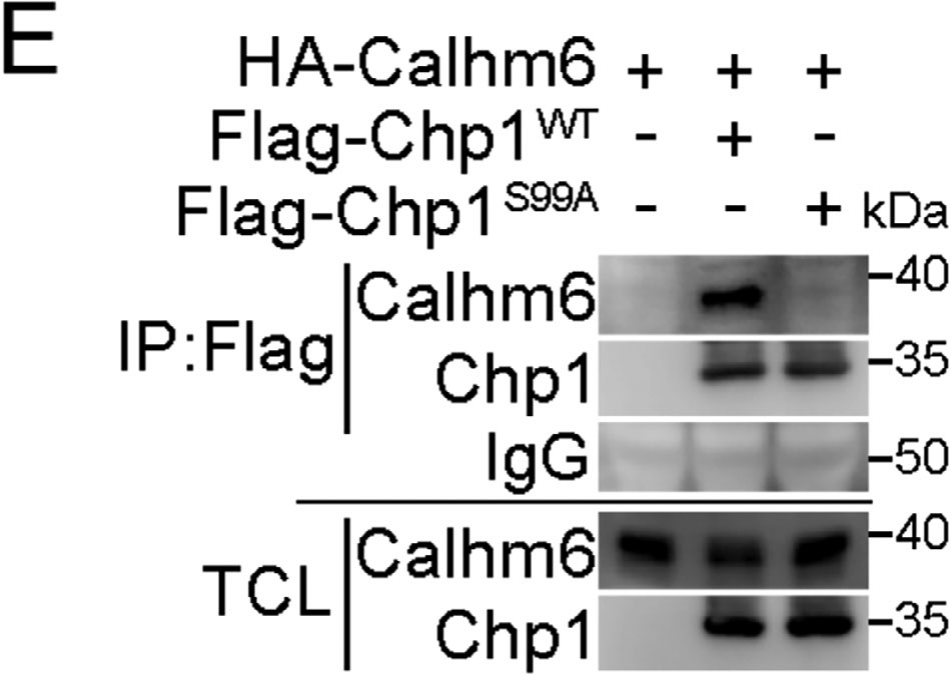


Corrected Figure 7E

All errors were caused by inadvertent copy‐paste mistakes during figure preparation. These revisions do not affect the original experimental data, results, or core conclusions of this study.

We apologize for this error.

