# Supplementary material for: Calhm6 Governs Macrophage Polarization Through Chp1‐Camk4‐Creb1 Axis and Ectosomal Delivery in Inflammatory Responses
Source: Adv Sci (Weinh). 2025 Sep 26;13(1):e02395. doi: 10.1002/advs.202502395 (PMC12766987; doi:10.1002/advs.202502395)
Supplement: Supplementary file 1 — Supporting Information [file ADVS-13-e02395-s010.docx]

Supplementary Information for

**Calhm6 governs macrophage polarization through Chp1-Camk4-Creb1 axis and ectosomal delivery in inflammatory responses**

Yanlong Xin, Xiaofan Xiong, Yan Zhang, Siyu Zhang, Shuting Zhang, Yu Yang, Yingxue Liang, Lulu Zang, Xi Chen, Wenjuan Li, Issam Halalmeh, Rui Zhou, Zongfang Li, Haowen Liu, Jing Geng

Corresponding author: Jing Geng

Email: jgeng18@xjtu.edu.cn

**Inventory of Supplementary Information**

1. Figures S1 to S9
2. Tables S1 and S2

**Fig. S1.** Ectosome carrying calhm6 suppresses severe inflammation by LPS challenging

(A) The ectosomes analysis by sequential ultracentrifugation for mass spectrometry from wild-type mice serum after LPS treatment as indicated time. (B) ELISA of serum cytokines of wild-type mice (n = 6 per group per experiment) per-treated with PBS (control) or ectosomes from wild-type or Calhm6 knockout mice before 48 hours intraperitoneal injection of PBS or LPS (20 mg/kg). (C) Immunoblot analysis of Mmp2, Flotillin1 and Calhm6 in ectosomes perfricated from the serum of wild-type or Calhm6 knockout mice treated by LPS for indicated time. (D) The Calhm6 expression in main tissue was determined by qRT-PCR after 24 hours by LPS challenging. (E) Fluorescence microscopy of the localization of Calhm6 (green) and Flag-tagged Calhm6 (red) in wild-type or Calhm6 knockout BMDMs incubated with ectosomes for 12 hours from EV or OE-Calhm6 cells which does not or stably express Flag-Calhm6. Scale bars, 10 μm. (F) The diagram of collecting ectosomes from stable cell lines expressing GFP-Calhm6 by ultra-centrifugation and injecting them intravenously to C57BL/6J mice. (G) Flow cytometric analysis of GFP+ cells in the spleen of C57BL/6J mice, as determined with anti-CD45, anti-CD11b, anti-Gr1, anti-CD11c, anti-CD4, anti-CD8, anti-CD19 and anti-CD56 antibodies. (H) Volcano plot of DEGs between untreated and *E. coli* (MOI, 50), *S.aureus* (MOI, 50), LPS (1 μg/ml) *C.albicans*, (MOI, 30) stimulated BMDMs. (I) The expression of Calhm1~6 was measured by RT-PCR after with or without LPS (200 ng/ml), LTA (100 ng/ml), FSL-1 (100 ng/ml) and Pam3csk4 (100 ng/ml) treatment. The data represent the mean ± s.d. (n=3). *p < 0.05, **p < 0.01 and ***p < 0.001 compared with control, Student’s t-test.

**Fig. S2.** Ectosomal-calhm6 mitigates inflammation and enhances tissue repair via M2-like macrophage polarization

(A) Immunoblot analysis of Calhm6, Nos2, Arg1 and GAPDH in Supernatant ectosomes or cell lysates were collected from wild-type or Calhm6 knockout BMDMs treated with LPS, IFNγ combined or IL-4 for 24 hours. (B-D) The wild-type mice were exposed to 2.5% DSS in the drinking water for 3 days before i.p. injections of PBS, ectosomal-EV or ectosomal-Calhm6 for every other day until sacrificed; mice were sacrificed at day 9 and tissue samples were collected for H&E staining (B), the disease activity index of colitis (C) and the body weight (D) was assessed daily. (E-F) Immunofluorescence histochemistry of TUNEL staining (E) of liver sections at post-APAP 24 hours from wild-type mice treated with or without NAC (250 mg/kg) or ectosomal-EV or ectosomal-Calhm6 as indicated at 2.5 hours after a sublethal dose of APAP (200 mg/kg) administration. Quantification of the results in (F), presented as relative fluorescence intensity. Scale bars, 200 μm. The data represent the mean ± S.D. (n=3). NS, not significant (p > 0.05); ****p < 0.0001 compared with control, Student’s t-test.

**Fig. S3.** Loss of Calhm6 enhances M1-like macrophages through Creb1 inactivation

(A-D) Flow cytometric analysis of CD3^+^ T cell development (A), B220^+^ B cell (B), the naïve T cell (CD62L^high^CD44^low^) and the effector T cell (CD62L^low^CD44^high^) (C) populations in the spleen or lymph node of wild-type and Calhm6 knockout mice, with the indicated antibodies.

**Fig. S4.** Calhm6-deficient mice shows high bactericidal activity with severe inflammatory response and tissue damage

(A-B) mortality (A) and bacterial load (as 16s rRNA) in the liver, kidney, lung, spleen and heart (B) of wild-type mice (n = 10 per group per experiment) pretreated with PBS (control), ectosomal-EV or ectosomal-Calhm6 before sham treatment or sublethal CLP; tissue samples were collected for 24 hours. (C) Flow cytometry of EV or OE-Calhm6 cells uninfected or infected for 20 min at 37 °C or 4 °C with FITC-labeled *E. coli* (FITC-*E. coli*) at an MOI of 100. (D) Flow cytometry analyzing cellular ROS production of EV or OE-Calhm6 cells treated with LPS (200 ng/ml) or PBS (control) for 24 hours, followed by staining with CellROX (5µM, 30min). (E) Confocal microscopy of the localization of Rac1 in wild-type or Calhm6 knockout BMDMs, imaged after infected for 30 min with GFP–*E. coli* (green) (MOI 20), then immunostained with anti-Rac1 (red) and counterstained with DAPI (blue); Scale bars, 50 μm. (F) Pearson’ s correlation coefﬁcient values for localization of *E. coli* and Rac1 in BMDMs. The average Pearson’ s correlation coefﬁcients were calculated from 12 randomly selected infected cells in each group (n =8 cells examined). The data represent the mean ± S.D. (n=3). NS, not significant (p > 0.05); *p < 0.05 and **p < 0.01 compared with control, Student’s t-test.

**Fig. S5** Calhm6 functions as a Ca^2+^ permeable ion channel in macrophages, mediating calcium influx upon LPS stimulation

(A) Content of Calhm6 in Ectosomes secreted by Raw264.7 cells with OE-Calhm6^High^, OE-Calhm6^Higher^ and OE-Calhm6^Highest^ cells. (B) Comparison of the positions of D121 (magenta) and E119 (red) within the structures of Calhm1 (green) and Calhm6 (cycan), respectively. The Calhm1 monomer is derived from the cryo-EM structure of its octamer with PDB ID 8GMP, and the murine Calhm6 monomer structure is obtained from the AlphaFold database with ID AF-Q8C9E8-F1. (C) Confocal microscopy of Raw264.7 cells treated with LPS (200 ng/ml) or PBS (Control) for 30 min, then immunostained with anti-Calhm6 (green) and counterstained with DAPI (blue). Scale bars, 10 μm. (D) Immunoassay of OE-Calhm6 Raw264.7 cells and after treated with PBS or LPS for 3 hours, assessed in anti-Flag immunoprecipitates or total cell lysates, probed with tag antibodies; below, immunoblot analysis of total cell lysates (TCL) without immunoprecipitation. (E) Immunoblot analysis of total lysates (bottom) and immunoprecipitates (top) of siCtrl or siRNF115 in OE-Calhm6 Raw264.7 cells treated for 3 hours, assessed in anti-Flag immunoprecipitates or total cell lysates, probed with various antibodies; below, immunoblot analysis of total cell lysates (TCL) without immunoprecipitation. (F) Confocal microscopy of siCtrl or siRNF115 in OE-Calhm6 Raw264.7 cells treated with LPS (200 ng/ml) or PBS (Control) for 30 min, then immunostained with anti-Calhm6 (green) and counterstained with DAPI (blue). Scale bars, 10 μm. The data represent the mean ± S.D. (n=3). (G) Olympus 980 microscope was used to record changes in intracellular calcium concentration in different cells treated with various calcium channel inhibitors under LPS stimulation (200 ng/mL). OE-Calhm6 refers to Raw264.7 cells stably overexpressing Calhm6, while EV denotes control Raw264.7 cells (empty vector). The inhibitors included the TRP channel inhibitor HC-067047 (50 nM), the SOCE inhibitor BTP2 (2μM), the IP₃R inhibitor Xestospongin C (5μM), and the RyR inhibitor Dantrolene (30μM). Intracellular calcium was stained with 5 μM rhod-2 AM, and 100 consecutive images were acquired at 2s second intervals. A 0.5 μM ionomycin treatment served as a normalization reference. Each experiment was independently repeated four times. The left panel shows representative fluorescent images captured after LPS and ionomycin stimulation, while the right panel displays the trend of fluorescence values in time-series scans for individual cells (n=6). Fluorescence values were calculated using ImageJ by analyzing six cells per cell type. A dynamic video generated from the continuous imaging is provided separately (Video 1-6). (H) Fluorescence values of cells under LPS stimulation were normalized across different conditions. The ratio of the calcium fluorescence peak induced by LPS to that induced by ionomycin was used as a standardized index for LPS-induced intracellular calcium concentration changes. (I) (J) Expression levels of Calhm1, Calhm6, and Calhm6E119R at the mRNA and protein levels. (K) (L)Twelve hours after ectosome treatment for LPS induced septic mice, serum concentrations of proinflammatory cytokines and serum levels of organ damage biomarkers (CRE, BUN, ALT and AST). (M) Localization of Calhm1/2/6 in N2a cells visualized by High Sensitivity Structured Illumination Microscope (HiS-SIM). Calhm1, Calhm2, and Calhm6 harbor an HA tag at their N-terminus. Target proteins tagged with HA antibody were stained using Donkey anti-Mouse Alexa Fluor™ 555 (553/568). Red fluorescence indicates the cellular localization of the proteins. (N) N2a cells expressing Calhm1, Calhm2, or Calhm6 were fixed (4% PFA, 10 min at RT), followed by either non-permeabilization(left) or permeabilization (right) treatment. After antibody labeling and staining of the three proteins, detection was performed by flow cytometry. All three proteins carry an HA tag, and the HA-tagged target proteins were stained using Donkey anti-Mouse Alexa Fluor™ 555 (553/568). (O-R) Western blot analysis of Calhm1, Calhm2, and Calhm6 levels in cell surface and cytoplasm. N2a cells transfected with pCMV-HA-Calhm1/Calhm2/Calhm6 were incubated with excess SNHSB ( Sulfo-NHS-SS-Biotin, 5 mM) at room temperature for 30 minutes, then washed 3 times with PBS containing 100 mM glycine to terminate the reaction. Cells were lysed with 1% Triton X-100, and the lysate supernatant was incubated with Streptavidin Magnetic Beads at 4°C for 6 hours. Proteins bound to Streptavidin Magnetic Beads represent those localized on the cell surface, while unbound proteins correspond to cytosolic components. ATP1A3 served as a membrane protein marker. (P-R) Analysis of Calhm1, Calhm2, and Calhm6 levels in the cytosol and on the cell surface, respectively. TCL denotes total cell lysis, Mem denotes total membrane proteins (cell surface), and Cyto denotes total cytosolic proteins. The data represent the mean ± S.D. (n=3). NS, not significant (p > 0.05); *p < 0.05 and **p < 0.01 compared with control, Student’s t-test.

**Fig. S6.** Calhm6 promotes Creb1 activation and M2-like polarization of macrophages via the Chp1-CaMK4 axis

(A) Heatmap of Creb1 target genes expressed by EV or OE-Calhm6 treated with Go6983 (5 μM), Defactinib (1 μM), KN62 (10 μM), FR180204 (10 μM), MK2206 (3 μM) and H-89 (20 μM) for 12 hours. (B) Identiﬁcation of Calhm6, Chp1, and CaMK4 by mass spectrometry in a Flag-tagged Calhm6-precipitation assay in cell lysates of OE-Calhm6 cells untreated or treated with IL-4 (20 ng/ml) or CaCl_2_ (2 mM) (Coomassie blue staining). (C) The knock-down efficiency of Chp1 was monitored by RT-PCR. (D) RT-qPCR analysis of the mRNA levels of the indicated target genes of Creb1 in sh-Scr or sh-Chp1 cells. (E) M2-mark is highly expressed in Raw264.7 cells with overexpressed Chp1 (OE-Chp1). (F)Interaction between Chp1 and CaMK4, showing the domains involved (red arrows); numbers above and below diagrams indicate amino acid range of each domain. (G-H) Mass spectrometry analysis of phosphorylation sites of Chp1 (Flag-tagged) co-transfected with CaMK4 from 293T cells and immunoprecipitation with anti-Flag antibody, showing the schematic diagrams (F) and candidate peptide fragments (H) of Chp1. (I) Sequence conservation analysis of serine 99 identified in Chp1 in thirteen species. The data represent the mean ± S.D. (n=3). NS, not significant (p > 0.05); *p < 0.05 and **p < 0.01 compared with control, Student’s t-test.

**Fig. S7.** Chp1 and CaMK4 assemble with Calhm6 on membrane of macrophages during M2-like polarization

(A) RT-qPCR analysis of the mRNA levels of the indicated target genes of Creb1 in wild-type or Calhm6 knockout BMDMs. (B) HeLa cells were transfected with plasmids expressing Myc-tagged CaMK4, HA-tagged Chp1 and Flag-tagged Calhm6. Confocal microscopy shows the co-localization of Chp1 (red), CaMK4 (green) and Calhm6 (purple) at the cell periphery or cytosol. Images shown are representative of approximately 20 cells. Scale bar, 20 µm. (C) Immunoassays of 293T cells expressing various combinations (above lanes) of Flag-tagged Calhm6 and the fragment of GFP-tagged CaMK4 as indicated; immunoprecipitation with anti-GFP and analysis by immunoblot with the indicated antibodies; below, immunoblot analysis of total cell lysates (TCL) without immunoprecipitation. (D) Immunoassays (as in C) of 293T cells expressing various combinations (above lanes) of Flag-tagged Calhm6 and the fragment of GFP-tagged Chp1 as indicated. (E) Schematics of the secondary structure or functional domains of the Chp1, Calhm6 and CaMK4. The data represent the mean ± S.D. (n=3). NS, not significant (p > 0.05); *p < 0.05 and **p < 0.01 compared with control, Student’s t-test.

**Fig. S8.** Irf1 enhances Calhm6 transcription in response to LPS/IFNγ activation but is blocked by IL-4-Stat6 axis

(A) RT-PCR analysis of Calhm6 in 293T cells transfected with an empty vector (EV) or Flag-tagged Irf1. (B) Luciferase reporter activity of 293T cells transfected with a construct containing the 2-kb Calhm6 promoter, Flag-tagged Irf1, Irf4, Stat3, Stat4, Stat6 and Pparγ. (C) Identification of indicated genes by mass spectrometry in streptavidin-precipitation assays using biotinylated wild-type or ΔStat6 (-46~-37) Calhm6 gene 1-kb promoter (Taz p1000) DNA fragments in BMDMs of wild-type mice under IL-4 treatment for 12 hours followed by treated with or without LPS and IFNγ combined for 12 hours. The data represent the mean ± s.d. (n=3). NS, not significant (P > 0.05); *P < 0.05, **P < 0.01 compared with control, Student’s t-test.

**Fig. S9.** Hypothetical mechanism diagram of Calhm6 regulating macrophage polarization

LPS/IFNγ stimulation triggers macrophages to upregulate CALHM6 transcription via IRF1, resulting in the enhanced secretion of CALHM6 within ectosomes that are internalized by neighboring macrophages. Under conditions such as elevated calcium, CALHM6, CHP1, and CAMK4 assemble on the cell membrane, initiating a signaling cascade through CHP1-CAMK4 that promotes Creb1 phosphorylation and drives macrophage polarization toward an M2-like phenotype. Additionally, IL4 enhances the binding affinity of STAT6 to the CALHM6 promoter, concurrently suppressing IRF1-mediated transcriptional activation of CALHM6.

**Supplementary Table 1.** **Characteristics of the Healthy and Bacterial pneumonia groups.**

| **Characteristics** | | **Healthy**  **(n=47)** | **Bacterial pneumonia (n=39)** |
| --- | --- | --- | --- |
| Sex | Female | 21 (44.68%) | 12 (30.76%) |
|  | Male | 26 (55.31%) | 27 (69.23%) |
| Age at study, median (IQR), years | | 46 (27~65) | 42 (1~87) |

**Supplementary Table 2. The primer list used in this study for plasmids construction, QPCR analysis, Chip assay, site mutations or shRNA knocking down.**

Species: h., human; m., mouse

| **Gene name** | **Forward primer sequence (5’-3’)** | **Reverse primer sequence (5’-3’)** | **Vector** |
| --- | --- | --- | --- |
| m.*CALHM6* | CGTCGACGGAGATCTATGGAAAAGTTCAAGGCAGT | CGCAAGCTTCTCGAGTCATAGTTCGTGAGTGTTAG | pCMV |
| m.*CAMK4* | CGTCGACGGAGATCTATGCTCAAAGTCACGGTGCCCTC | CGCAAGCTTCTCGAGTTAGTACTCTGGCTGAATCGC | pCMV |
| m.*CHP1* | CGTCGACGGAGATCTATGGGGTCTCGGGCCTCCACG | CGCAAGCTTCTCGAGTTAGTGAAGAAATCGGATGCTC | pCMV |
| m.*CHP1* | TGAGAGCCTTGGCTCATTTCCGACCCAT | ATGAGCCAAGGCTCTCATGAATCCTCGGAAGT | pCMV |
| m.*IRF1* | CGTCGACGGAGATCTATGCCAATCACTCGAATGCGGA | CGCAAGCTTCTCGAGCTATGGTGCACAAGGAATGGC | pCMV |
| m.*STAT6* | CGTCGACGGAGATCTATGTCTCTGTGGGGCCTAATTTC | CGCAAGCTTCTCGAGTCACCAGCTGGGGTTGGTCCTTA | pCMV |
| m.*CREB1* | CGTCGACGGAGATCTATGACCATGGAATCTGGAGCAGAC | CGCAAGCTTCTCGAGTTAATCTGATTTGTGGCAGTAAAG | pCMV |
| m.*CALHM6* | CGTCGACGGAGATCTATGGAAAAGTTCAAGGCAGT | CGCAAGCTTCTCGAGTCATAGTTCGTGAGTGTTAG | pLV |
| m.*CALHM6*  (ISO) | AATTCGGATCCATGACCGCGGCATTCGCGCCC | GCCGCGGTCATGGATCCGAATTCTCTAGACTT | pLV |
| m.*C-FOS* | CGTCGACGGAGATCTATGATGTTCTCGGGTTTCAACG | CGCAAGCTTCTCGAGTCACAGGGCCAGCAGCGTGGGT | pCMV |
| m.*C-JUN* | CGTCGACGGAGATCTATGACTGCAAAGATGGAAACGA | CGCAAGCTTCTCGAGTCAAAACGTTTGCAACTGCTGC | pCMV |
| m.*IRF3* | CGTCGACGGAGATCTATGGAAACCCCGAAACCGCGG | CGCAAGCTTCTCGAGTCAGATATTTCCAGTGGCCTG | pCMV |
| m.*IRF8* | CGTCGACGGAGATCTATGTGTGACCGGAACGGCGGGC | CGCAAGCTTCTCGAGTTAGACGGTGATCTGTTGATT | pCMV |
| m.*STAT1* | CGTCGACGGAGATCTATGTCACAGTGGTTCGAGCTT | CGCAAGCTTCTCGAGTTATACTGTGCTCATCATACT | pCMV |
| m.*STST5A* | CGTCGACGGAGATCTATGGCGGGCTGGATCCAGGCCC | CGCAAGCTTCTCGAGTCATGAGAGGGAGCCTCTGG | pCMV |
| m.*RELA* | CGTCGACGGAGATCTATGGACGATCTGTTTCCCCTCA | CGCAAGCTTCTCGAGTTAGGAGCTGATCTGACTCAAA | pCMV |
| m.*RELB* | CGTCGACGGAGATCTATGCCGAGTCGCCGCGCTGCCA | CGCAAGCTTCTCGAGCTACGTGGCTTCAGGCCCTGGA | pCMV |
| m.*IRF4* | CGTCGACGGAGATCTATGAACTTGGAGACGGGCA | CGCAAGCTTCTCGAGTCACTCTTGGATGGAAGAATGACG | pCMV |
| m.*STAT3* | CGTCGACGGAGATCTATGGCTCAGTGGAACCAGCTG | CGCAAGCTTCTCGAGTCACATGGGGGAGGTAGCACA | pCMV |
| m.*STAT4* | CGTCGACGGAGATCTATGTCTCAGTGGAATCAAGTCC | CGCAAGCTTCTCGAGTCATTCAGCAGAATATGGGGAA | pCMV |
| m.*PPARG* | CGTCGACGGAGATCTATGGGTGAAACTCTGGGAGATT | CGCAAGCTTCTCGAGCTAATACAAGTCCTTGTAGAT | pCMV |
| m.*CALHM6*  (1-128) | CGTCGACGGAGATCTATGGAAAAGTTCAAGGCAGT | CGCAAGCTTCTCGAGGCTGACAGCACATTGGTAGA | pCMV |
| m.*CALHM6*  (129-203) | CGTCGACGGAGATCTGGGAGCGCGCGCTTGGCGCC | CGCAAGCTTCTCGAGCAGATAACTAACCGGAGAGAA | pCMV |
| m.*CALHM6*  (204-315) | CGTCGACGGAGATCTCAGTTAAAATTCTGGGAAATC | CGCAAGCTTCTCGAGTCATAGTTCGTGAGTGTTAG | pCMV |
| m.*CHP1*  (1-61) | CGTCGACGGAGATCTATGGGGTCTCGGGCCTCCACG | CGCAAGCTTCTCGAGTGGGTTGATGGCAAGTTCT | pCMV |
| m.*CHP1*  (62-106) | CGTCGACGGAGATCTCTGGGGGACCGGATCATCAA | CGCAAGCTTCTCGAGTTCCGGGCCATTCACATC | pCMV |
| m.*CHP1*  (107-150) | CGTCGACGGAGATCTCCCCTCAACAGCCGGAGC | CGCAAGCTTCTCGAGCTCATCCGAGATATTCACTC | pCMV |
| m.*CHP1*  (151-195) | CGTCGACGGAGATCTCAGCTGGGCAGTATTGCGGA | CGCAAGCTTCTCGAGTTAGTGAAGAAATCGGATGCTC | pCMV |
| m.*CAMK4*  (1-296) | CGTCGACGGAGATCTATGCTCAAAGTCACGGTGCCCTC | CGCAAGCTTCTCGAGGTGTTGGAGGGCTTGAAATG | pCMV |
| m.*CAMK4*  (297-336) | CGTCGACGGAGATCTCCATGGGTCACAGGTAAAGCG | CGCAAGCTTCTCGAGTCCCAGCCGAGAAGAGGCC | pCMV |
| m.*CAMK4*  (337-470) | CGTCGACGGAGATCTAGTGCCAGCAGTAGCCACACC | CGCAAGCTTCTCGAGTTAGTACTCTGGCTGAATCGC | pCMV |

| **qPCR primers** | **Forward primer sequence (5’-3’)** | **Reverse primer sequence (5’-3’)** |
| --- | --- | --- |
| m. *GAPDH* | AGGTCGGTGTGAACGGATTTG | TGTAGACCATGTAGTTGAGGTCA |
| h. *GAPDH* | CAATGACCCCTTCATTGACC | GACAAGCTTCCCGTTCTCAG |
| m. *CALHM1* | GCCACACTAGGCAATGGCA | GCAGCCAGTTCCCATCATAGA |
| m. *CALHM2* | TCTTCAAGAGCAAGGATGTGATG | TCAGTCCATACAGGTAGTTCCG |
| m. *CALHM3* | GCTGCACTTCTTCGCAAACA | CGTTTCCACTATCCCGGTCC |
| m. *CALHM4* | TCAGAAGTGAACCGTGCATCA | AGGCAGGAACCACAAGAAAAG |
| m. *CALHM5* | TTATGAATGTGCTATGAGCGGG | GATAGCCTCACTTCTTCACTCTC |
| m. *CALHM6* | AGTCTCAGGTGTTCGGTTGGA | GGCTTCGAGCACTCAAAGAAAC |
| m. *HMGB1* | GGCGAGCATCCTGGCTTATC | GGCTGCTTGTCATCTGCTG |
| m. *IL6* | TAGTCCTTCCTACCCCAATTTCC | TTGGTCCTTAGCCACTCCTTC |
| m. *IL1B* | GCAACTGTTCCTGAACTCAACT | ATCTTTTGGGGTCCGTCAACT |
| m. *TNFA* | CCCTCACACTCAGATCATCTTCT | GCTACGACGTGGGCTACAG |
| m. *CHIL3* | CGTTCAGTGCCATGGTCTCT | CTTCCTCGAGACCCAGGGTA |
| m. *COX2* | TTCAACACACTCTATCACTGGC | AGAAGCGTTTGCGGTACTCAT |
| m. *Fizz1* | CCAATCCAGCTAACTATCCCTCC | ACCCAGTAGCAGTCATCCCA |
| m. *IL12* | CTGTGCCTTGGTAGCATCTATG | GCAGAGTCTCGCCATTATGATTC |
| m. *IRF1* | GGCCGATACAAAGCAGGAGAA | GGAGTTCATGGCACAACGGA |
| m. *IRF3* | GAGAGCCGAACGAGGTTCAG | CTTCCAGGTTGACACGTCCG |
| m. *CREB* | AGCCGGGTACTACCATTCTAC | GCAGCTTGAACAACAACTTGG |
| m. *NOS2* | GTTCTCAGCCCAACAATACAAGA | GTGGACGGGTCGATGTCAC |
| m. *ARG1* | CTCCAAGCCAAAGTCCTTAGAG | AGGAGCTGTCATTAGGGACATC |
| m. *PPARC* | TCGCTGATGCACTGCCTATG | GAGAGGTCCACAGAGCTGATT |
| m. *IL10* | GCTCTTACTGACTGGCATGAG | CGCAGCTCTAGGAGCATGTG |
| m. *TGFB1* | CTCCCGTGGCTTCTAGTGC | GCCTTAGTTTGGACAGGATCTG |
| m. *MRC1* | CTCTGTTCAGCTATTGGACGC | CGGAATTTCTGGGATTCAGCTTC |
| m. *BDNF* | TCATACTTCGGTTGCATGAAGG | AGACCTCTCGAACCTGCCC |
| m. *NURR1* | GTGTTCAGGCGCAGTATGG | TGGCAGTAATTTCAGTGTTGGT |
| m. *Puma* | GGCTTCTCCGCGAATGACAA | GTTTGGACGCAGCATCTGGA |
| m. *Noxa* | ACGCGAAGACTGGGACTCT | AGCCCCTGTTAAAGTACATCCTA |
| m. *EDN1* | GCACCGGAGCTGAGAATGG | GTGGCAGAAGTAGACACACTC |
| m. *GADD45B* | CAACGCGGTTCAGAAGATGC | GGTCCACATTCATCAGTTTGGC |
| m. *NR4A1* | TTGAGTTCGGCAAGCCTACC | GTGTACCCGTCCATGAAGGTG |
| m. *PTGS2* | TTCAACACACTCTATCACTGGC | AGAAGCGTTTGCGGTACTCAT |
| m. *CSRNP1* | TCCAGAGTTTCACTCCCCC | GGCACCGTGGGAAATAGTAGA |
| m. *DUSP1* | GTTGTTGGATTGTCGCTCCTT | TTGGGCACGATATGCTCCAG |
| m. *C-Fos* | TTGAGCGATCATCCCGGTC | GCGTGAGTCCATACTGGCAAG |
| *16s rRNA* | GTGGTGCATGGTTGTCGTCA | ACGTCGTCCCCACCTTCCTC |
| h. *IFNG* | TCGGTAACTGACTTGAATGTCCA | TCGCTTCCCTGTTTTAGCTGC |
| h*. TNFA* | CCTCTCTCTAATCAGCCCTCTG | GAGGACCTGGGAGTAGATGAG |
| h. *IL12* | CCTTGCACTTCTGAAGAGATTGA | ACAGGGCCATCATAAAAGAGGT |
| h. *CALHM6* | TGTCACCCGATGCCTATCTC | TGGCCCTTCGGATTGAAAGTA |

| **shRNA** | **Forward sequence (5’-3’)** | **Reverse sequence (5’-3’)** | **Vector** |
| --- | --- | --- | --- |
| m. Control shRNA | CCGGCCGTCATGATCTCGCACTTCAACTCGAGGTTATCCTCAATGGGTCGGAATTTTTG | AATTCAAAAATTGAAGTGCGAGATCATGACGGCCTCGAGGTTATCCTCAATGGGTCGGAA | pLKO |
| m. sh-Chp1-1 | CCGGTTCCGACCCATTGAGGATAACCTCGAGGTTATCCTCAATGGGTCGGAATTTTTG | AATTCAAAAATTCCGACCCATTGAGGATAACCTCGAGGTTATCCTCAATGGGTCGGAA | pLKO |
| m. sh-Chp1-2 | CCGGCAACCCACTGGGGGACCGGACCTCGAGGTTATCCTCAATGGGTCGGAATTTTTG | AATTCAAAAATCCGGTCCCCCAGTGGGTTGCCTCGAGGTTATCCTCAATGGGTCGGAA | pLKO |
| m. sh-Chp1-3 | CCGGTTTTACAGAATTCGTTAAGGCCTCGAGGTTATCCTCAATGGGTCGGAATTTTTG | AATTCAAAAACCTTAACGAATTCTGTAAAACCTCGAGGTTATCCTCAATGGGTCGGAA | pLKO |
| m. sh-Calhm6-1 | CCGGGCTTCTCTCCGGTTAGTTATCCTCGAGGATAACTAACCGGAGAGAAGCTTTTTG | AATTCAAAAAGCTTCTCTCCGGTTAGTTATCCTCGAGGATAACTAACCGGAGAGAAGC | pLKO |
| m. sh- Calhm6-2 | CCGGGCAACACTCCAAGCAGTAAAGCTCGAGCTTTACTGCTTGGAGTGTTGCTTTTTG | AATTCAAAAAGCAACACTCCAAGCAGTAAAGCTCGAGCTTTACTGCTTGGAGTGTTGC | pLKO |
| m. sh- Calhm6-3 | CCGGGGTGACCCTACTGACGGCTCTCGAGAGCCGTCAGTAGGGTCACCTTTTTG | AATTCAAAAAGGTGACCCTACTGACGGCTCTCGAGAGCCGTCAGTAGGGTCACC | pLKO |

| **Others** | **Forward primer sequence (5’-3’)** | **Reverse primer sequence (5’-3’)** | **Vector** |
| --- | --- | --- | --- |
| m.*CHP1*  (S99A) | ACGAAAAGGCCAAAGATGTGAATGGCCCGGA | ATCTTTGGCCTTTTCGTTATCCTCAATGG | pCMV |
| m.*CALHM6* promoter  p2-kb | CTCGCTAGCCTCGAGGAGTACAAAATAGGATCTCAGCACT | CTCGAGGCTAGCGAGCTCAGGTACCGGC | PGL4.10 |
| m.*CALHM6* (p)1-kb | GAGCTCGCTAGCCTCGAGTCCAGATATAGATCAGGCTAG | CTCGAGGCTAGCGAGCTCAGGTACCGGC | PGL4.10 |
| m.*CALHM6* promoter  (-46~-37) deletion | TCACTCAACCGCAGAGCCTGTGGGGTGTCCAG | CAGGCTCTGCGGTTGAGTGAACCGTGTACTGT | PGL4.10 |
| m.*CALHM6* promoter  (-440~-430) deletion | GCCTATAAACGGCTGCAATTGCTAGTTTTAC | AATTGCAGCCGTTTATAGGCTGTGACTTCTG | PGL4.10 |
| m.*CALHM6* promoter  (-1227~-1218) deletion | TCTAGCTTTCTCCACGTTATGAAATAGTAGA | AACGTGGAGAAAGCTAGAAAGAGCACGAGCT | PGL4.10 |
| m.*CALHM6* promoter  (-1374~-1364) deletion | GTTGTACAAAAGGAGAGTAAGTTGTAACC | TCTCCTTTTGTACAACAGTTAACTTTAT | PGL4.10 |
| m.*CALHM6* promoter  (-127~-118) deletion | GATTGAAAAAAAAGACCCGGAAGGGTTTCCT | TCTTTTTTTTCAATCACACCAGTTCTGGTA | PGL4.10 |
| m.*CALHM6* promoter  (-713~-705) deletion | GGGTTGGAGGAAACAGAACCGGAGTTTTCTTGAAAGC | GTTCTGTTTCCTCCAACCCAAAGTGACCTCTTACAGTG | PGL4.10 |
| m.*CALHM6* promoter  (-865~-856) deletion | ATGAAAGTGTTCCTGTTTCCTGTAAATGTTGTGGGTT | AACAGGAACACTTTCATAAGCTAAACCACAGTAAAACTG | PGL4.10 |
| m.*CALHM6* promoter  (-1148~-1140) deletion | AACTACCAGGAGGGCAGGAGTCATGTTAGATGTAGCTT | AACATGACTCCTGTCCCTCCTGGTAGTTCTTCTGAT | PGL4.10 |
| ChIP assay primers  for m.*CALHM6* promoter  (-130~ -30) | AAAGAAAGTGAAACCCGGAA | GCTCTGCTTCTCAGGAAGGT |  |
