# Supplementary figures and images for: Calhm6 Governs Macrophage Polarization Through Chp1‐Camk4‐Creb1 Axis and Ectosomal Delivery in Inflammatory Responses
Source: Adv Sci (Weinh). 2025 Sep 26;13(1):e02395. doi: 10.1002/advs.202502395 (PMC12766987; doi:10.1002/advs.202502395)

supplementary Figure-1

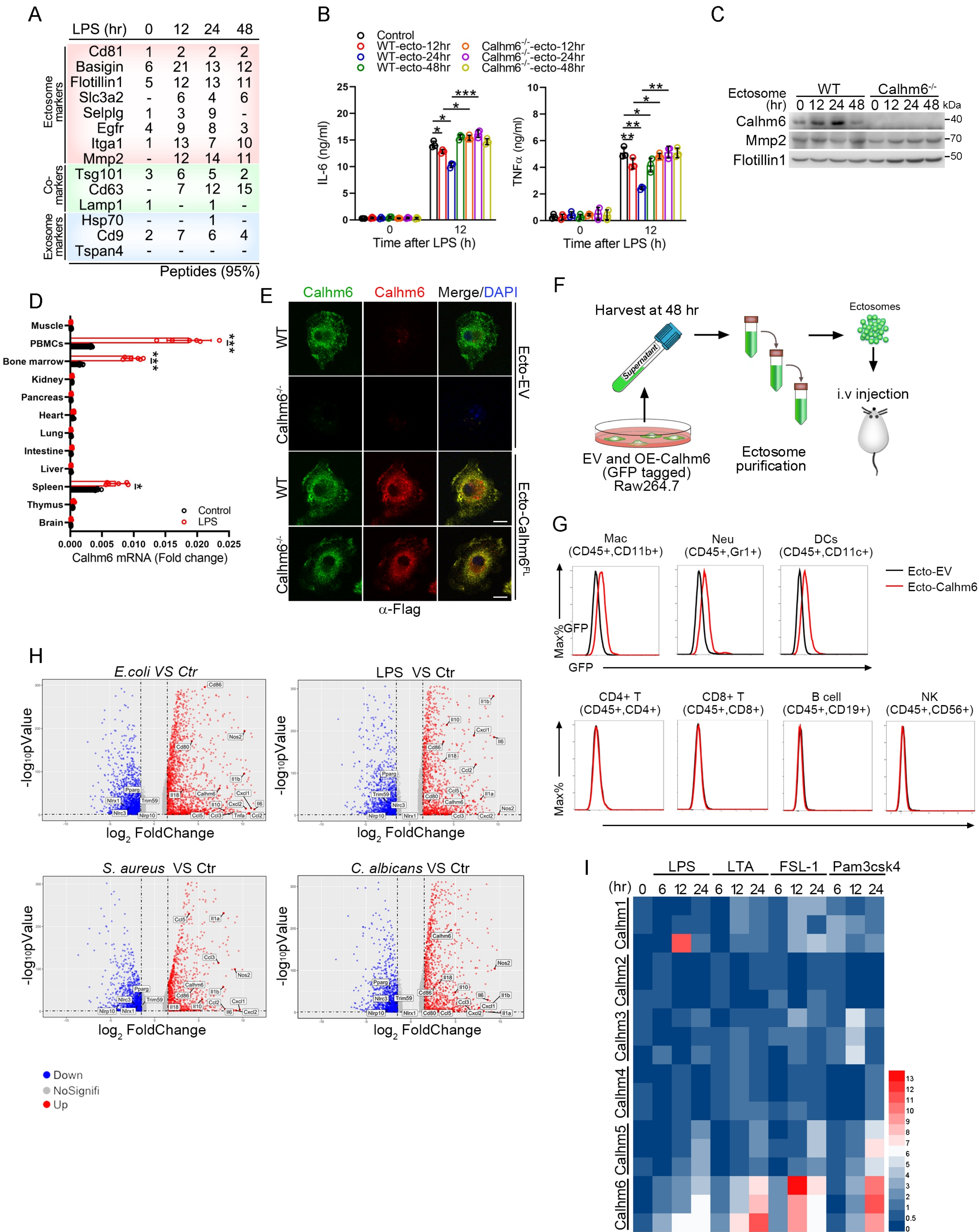

Supplement: Supplementary file 2 — Supporting Information [file ADVS-13-e02395-s003.pdf]

supplementary Figure-2

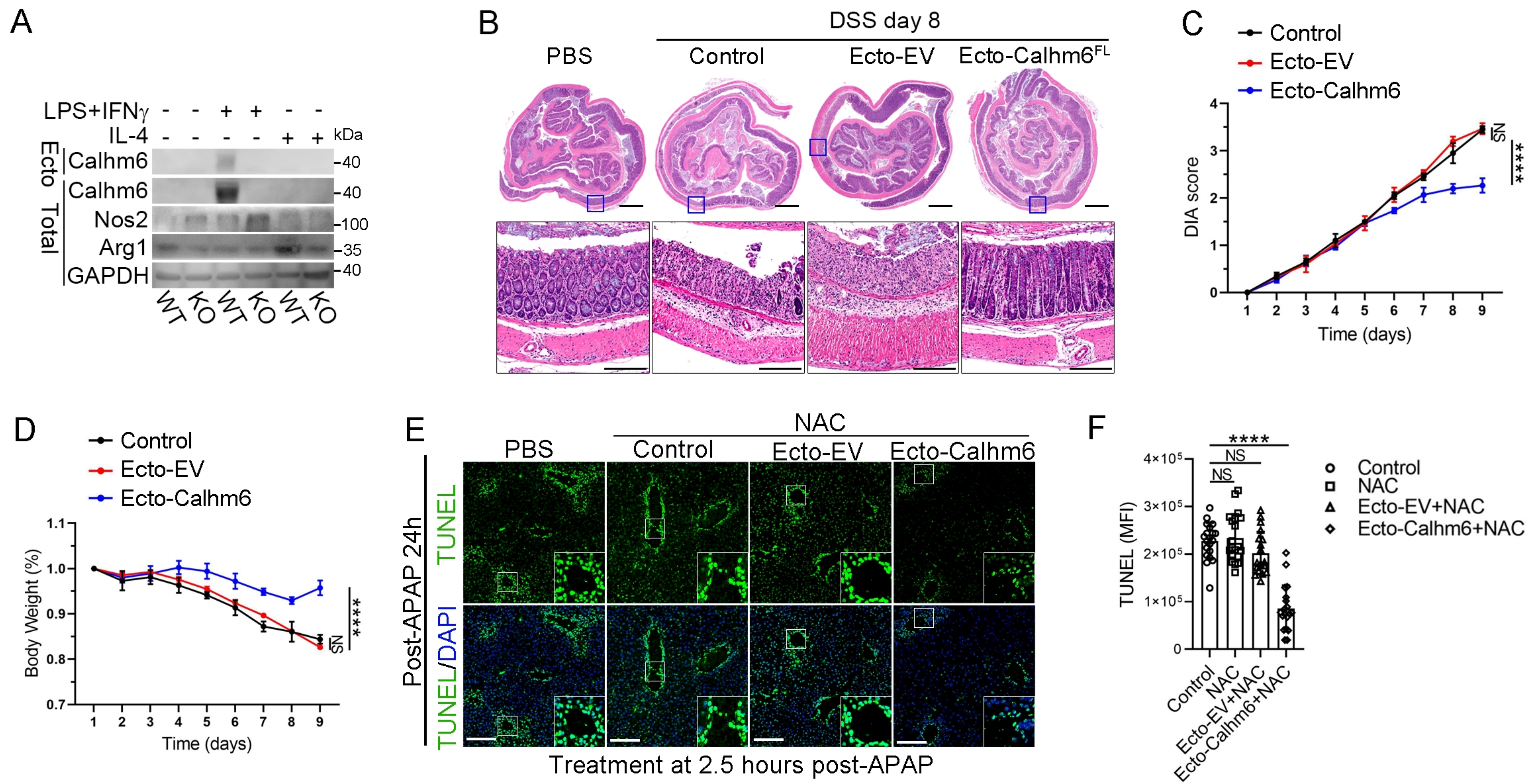

Supplement: Supplementary file 3 — Supporting Information [file ADVS-13-e02395-s001.pdf]

supplementary Figure-3

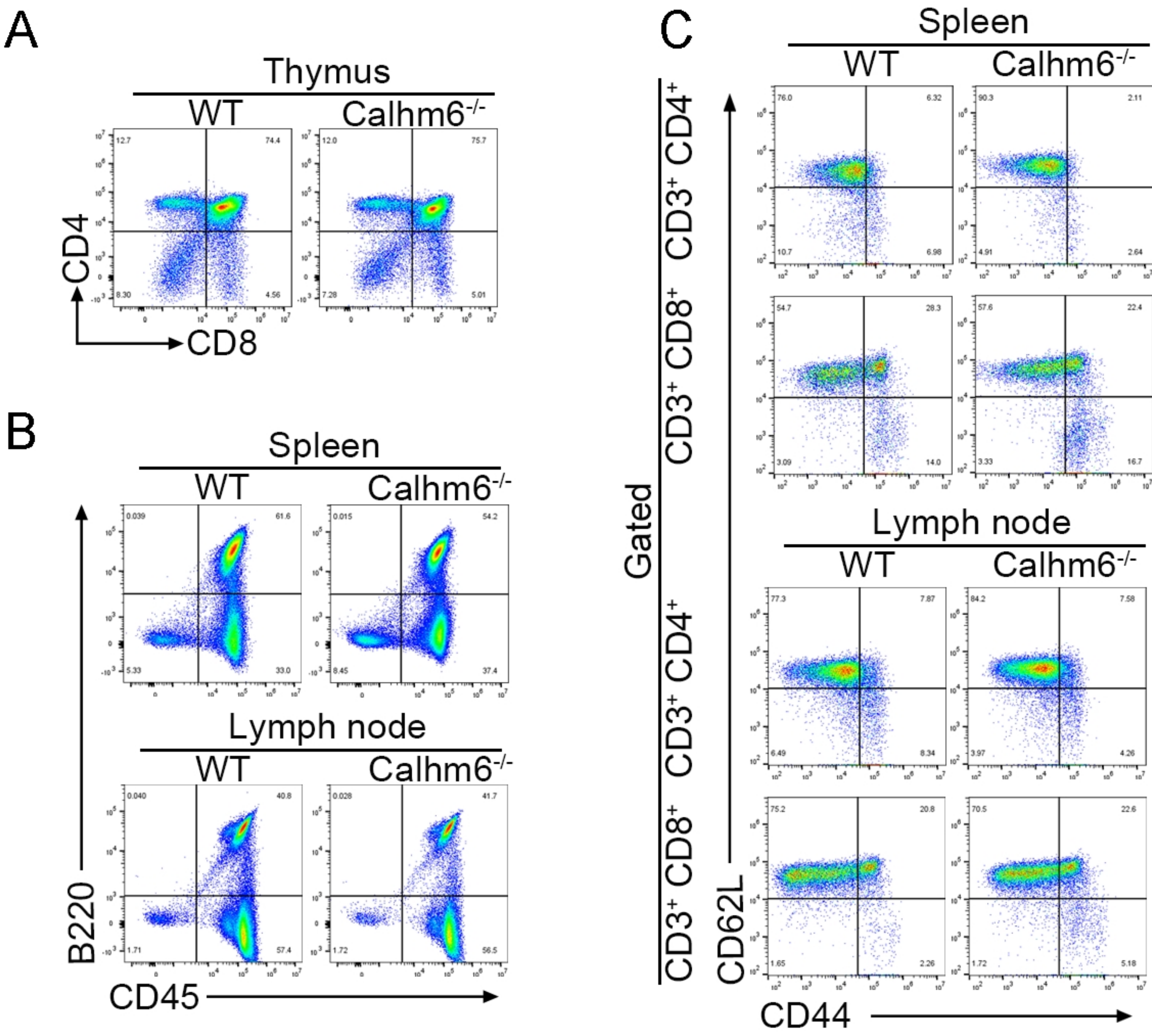

Supplement: Supplementary file 4 — Supporting Information [file ADVS-13-e02395-s009.pdf]

supplementary Figure-4

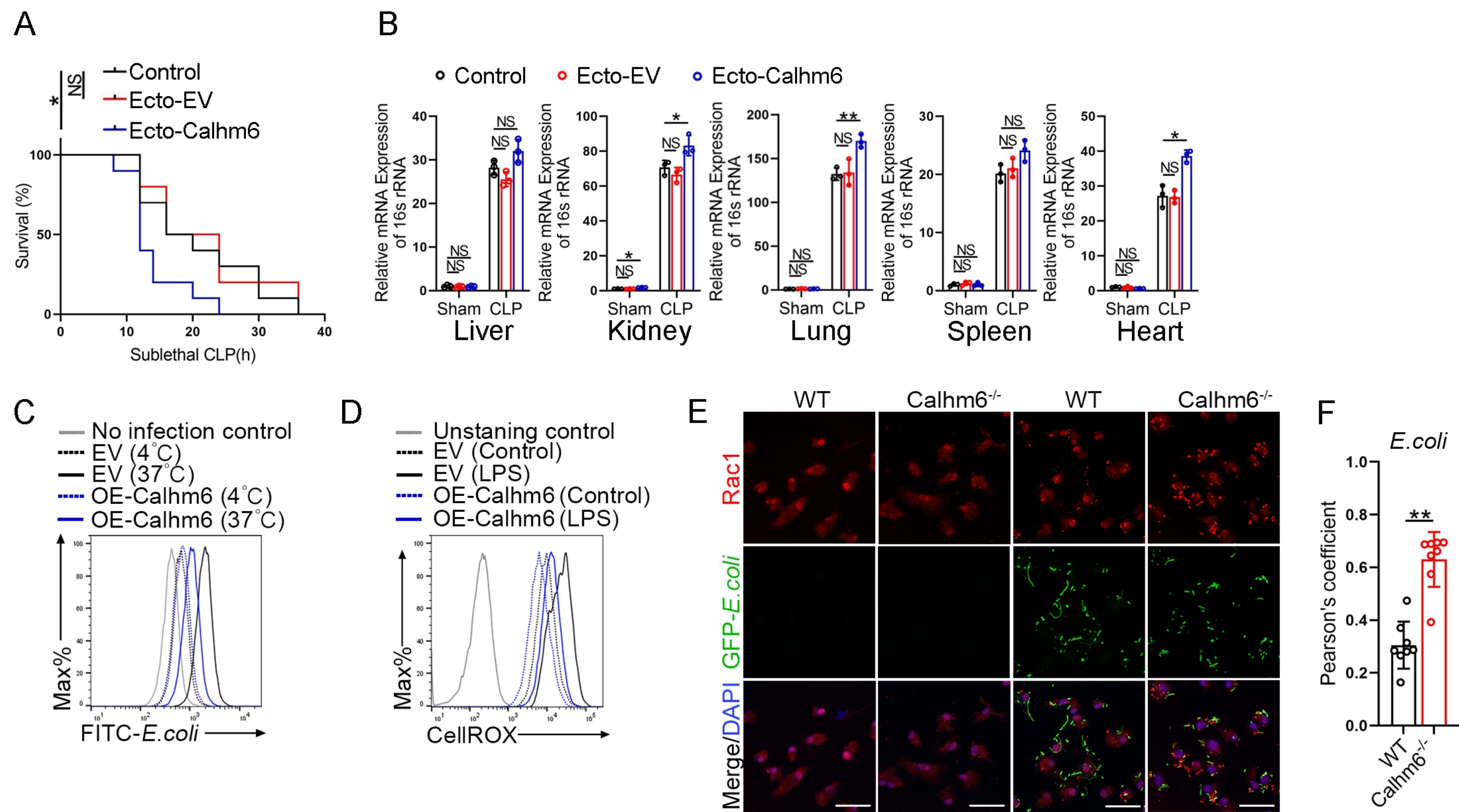

Supplement: Supplementary file 5 — Supporting Information [file ADVS-13-e02395-s011.pdf]

supplementary Figure-5

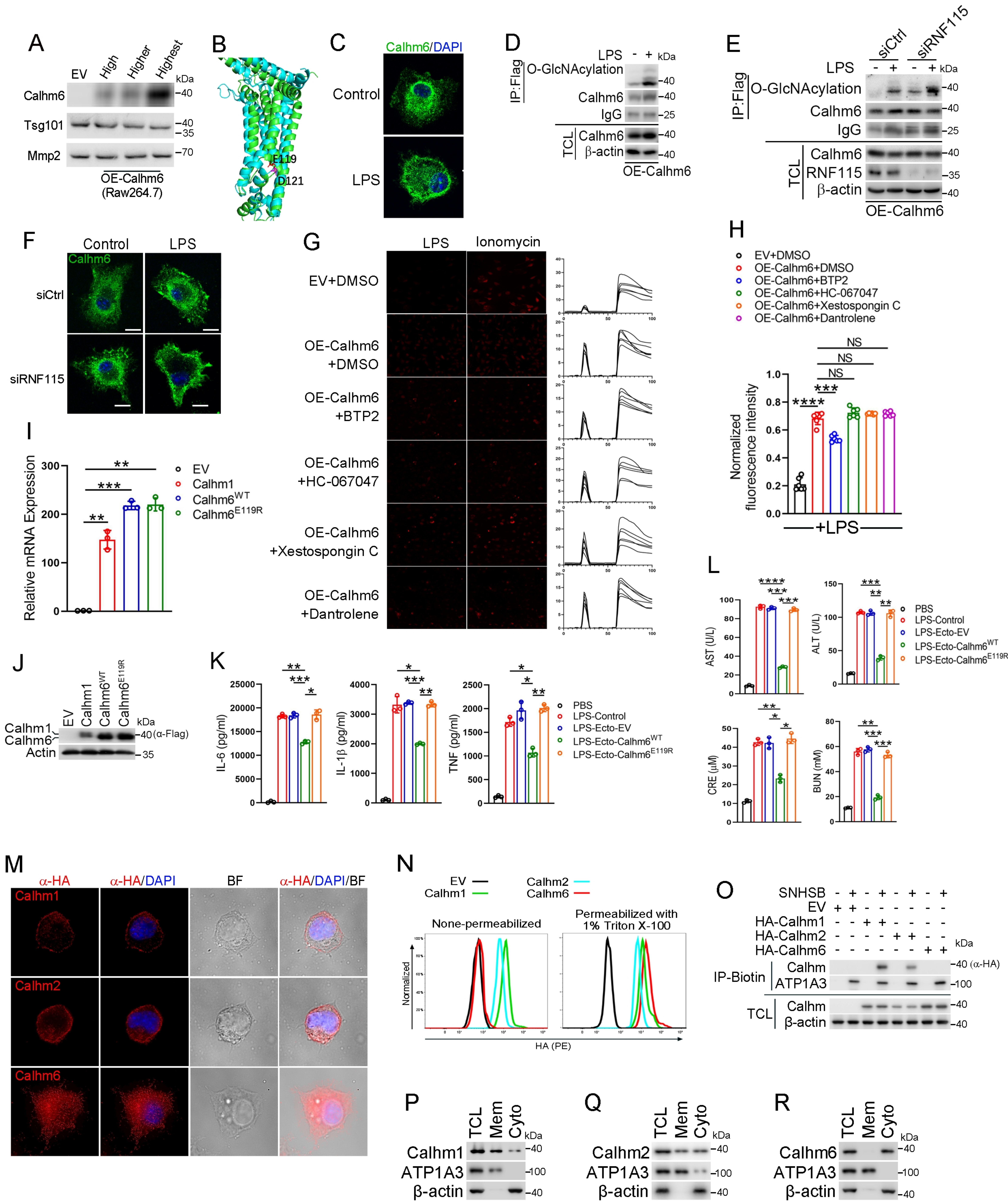

Supplement: Supplementary file 6 — Supporting Information [file ADVS-13-e02395-s002.pdf]

supplementary Figure-6

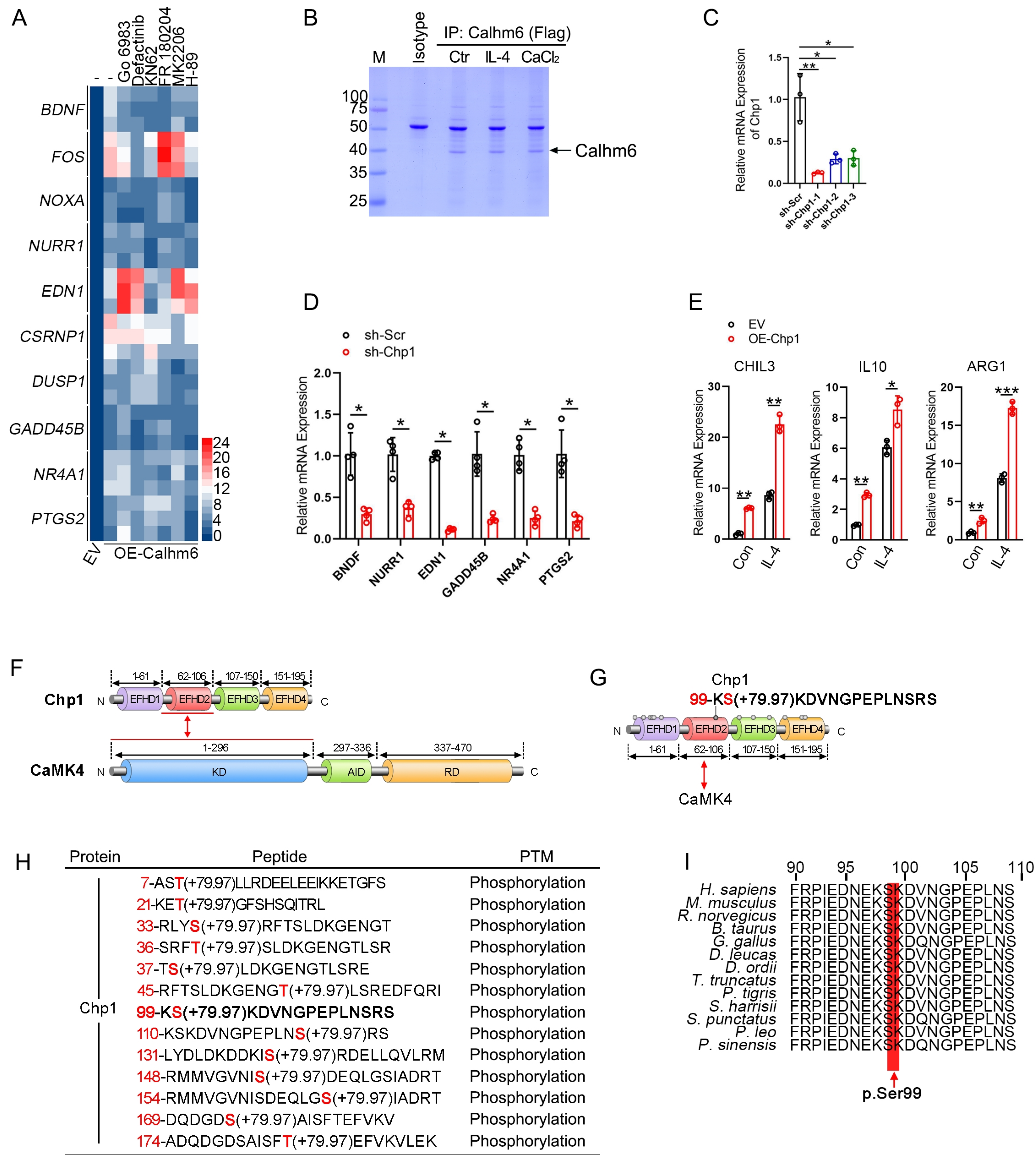

Supplement: Supplementary file 7 — Supporting Information [file ADVS-13-e02395-s005.pdf]

supplementary Figure-7

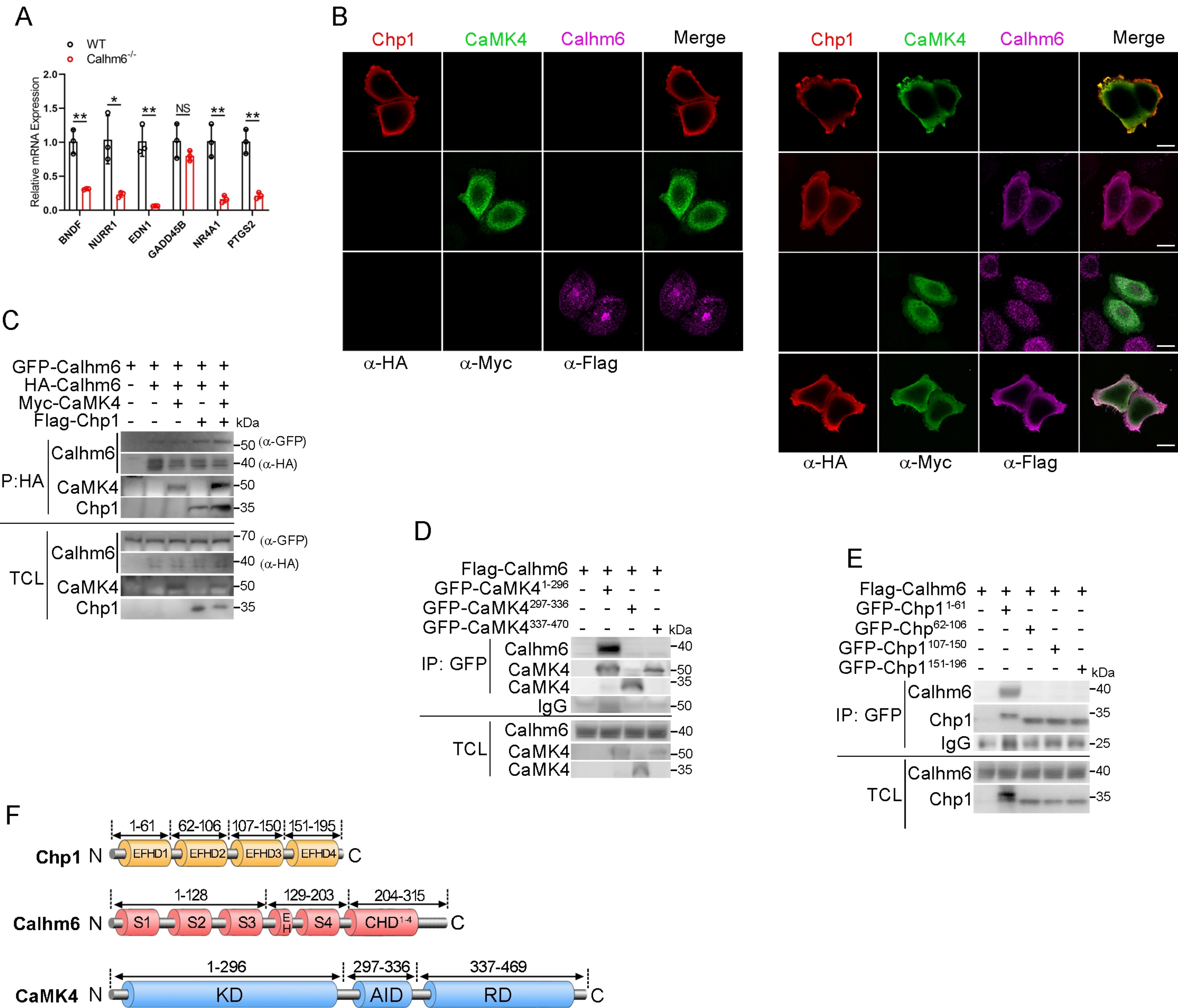

Supplement: Supplementary file 8 — Supporting Information [file ADVS-13-e02395-s007.pdf]

supplementary Figure-8

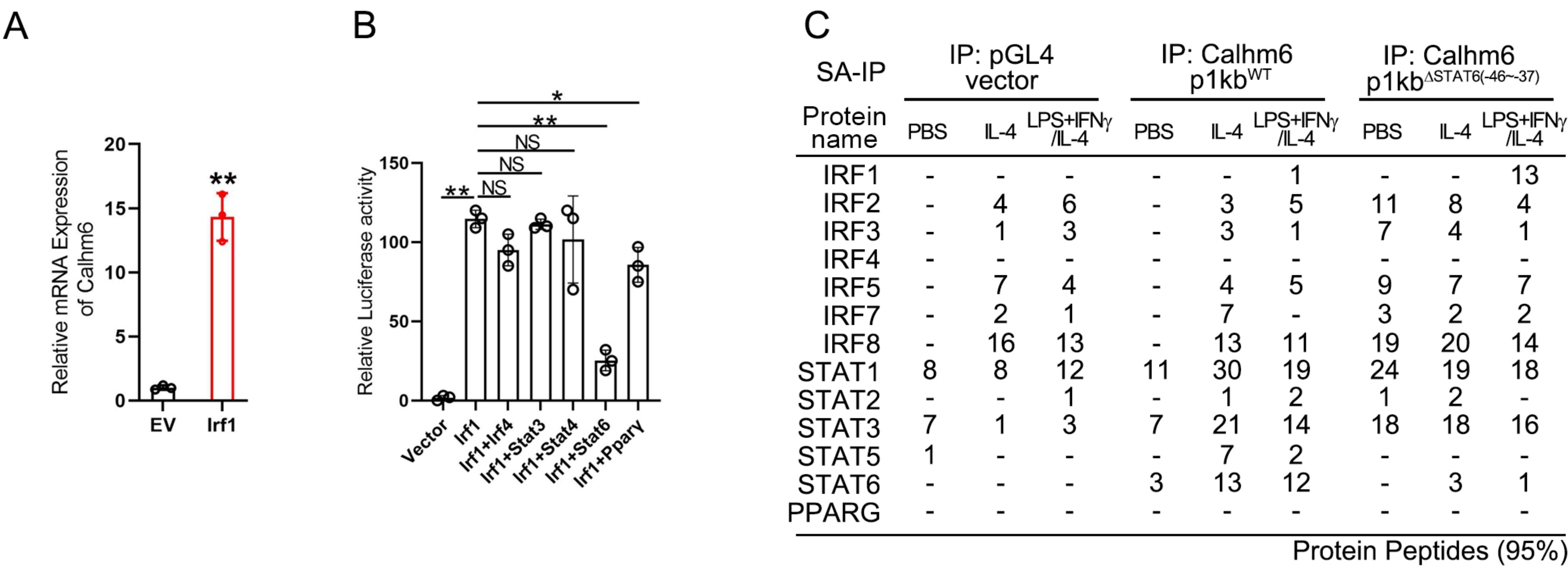

Supplement: Supplementary file 9 — Supporting Information [file ADVS-13-e02395-s006.pdf]

supplementary Figure-9

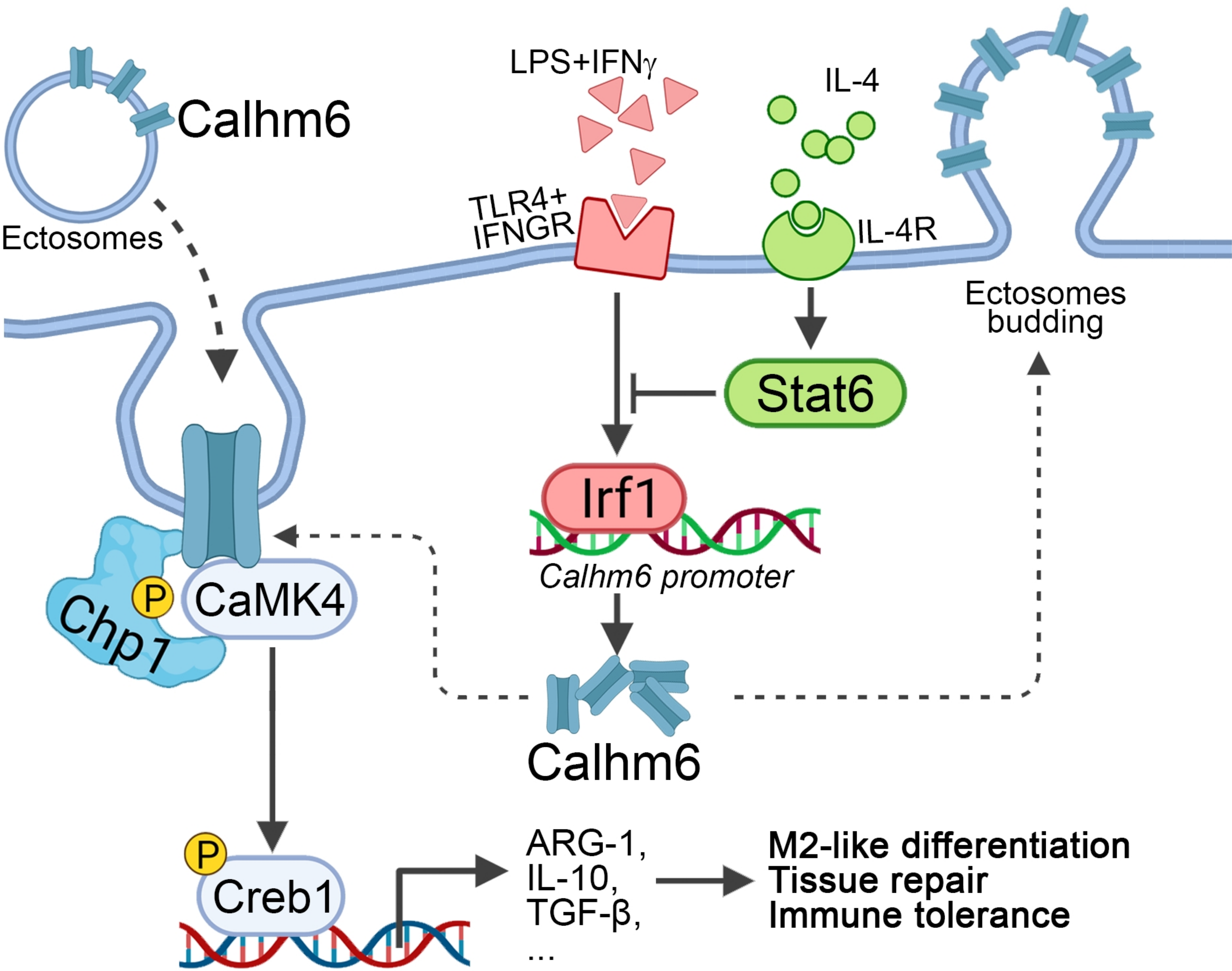

Supplement: Supplementary file 10 — Supporting Information [file ADVS-13-e02395-s008.pdf]

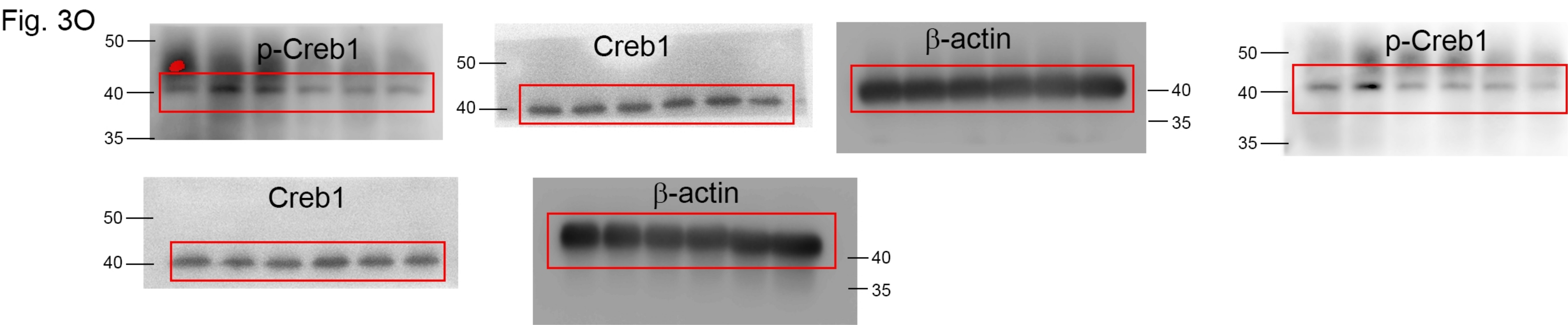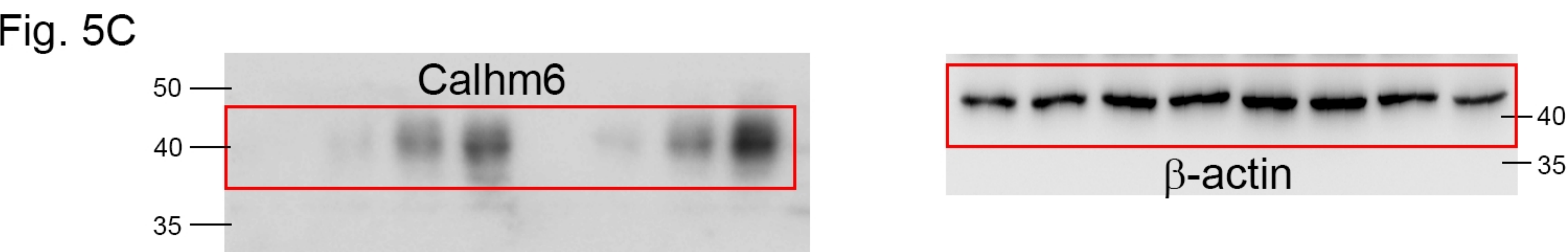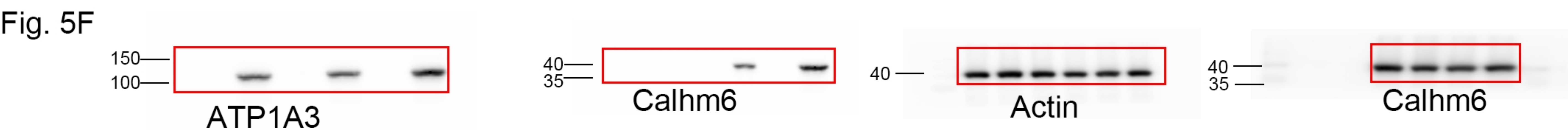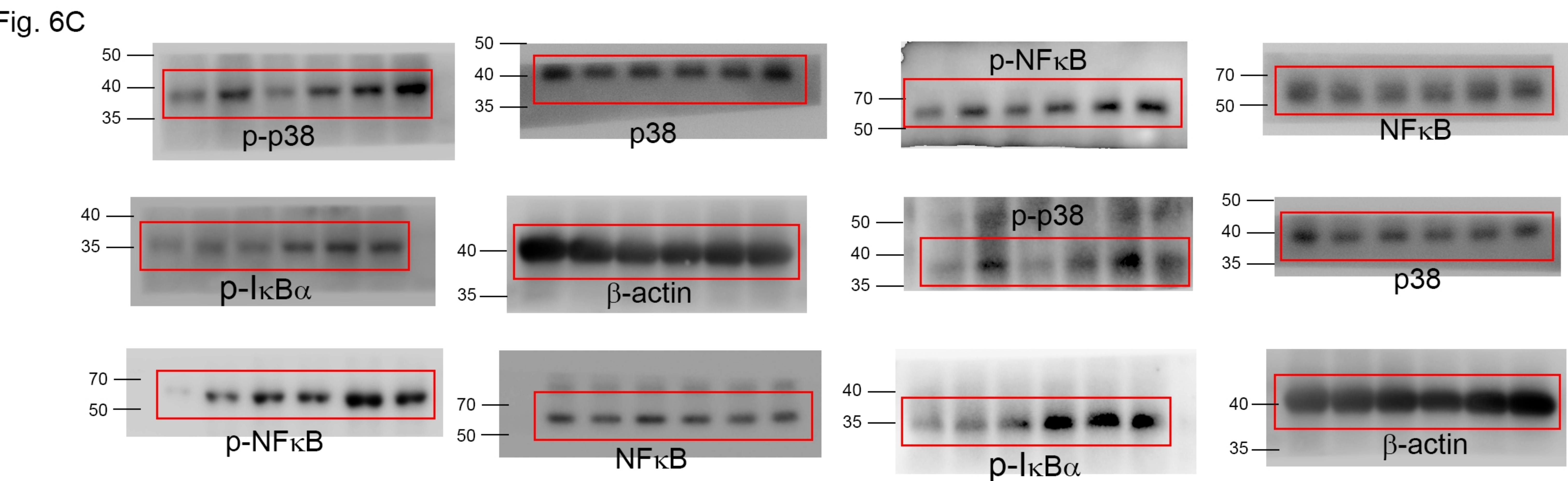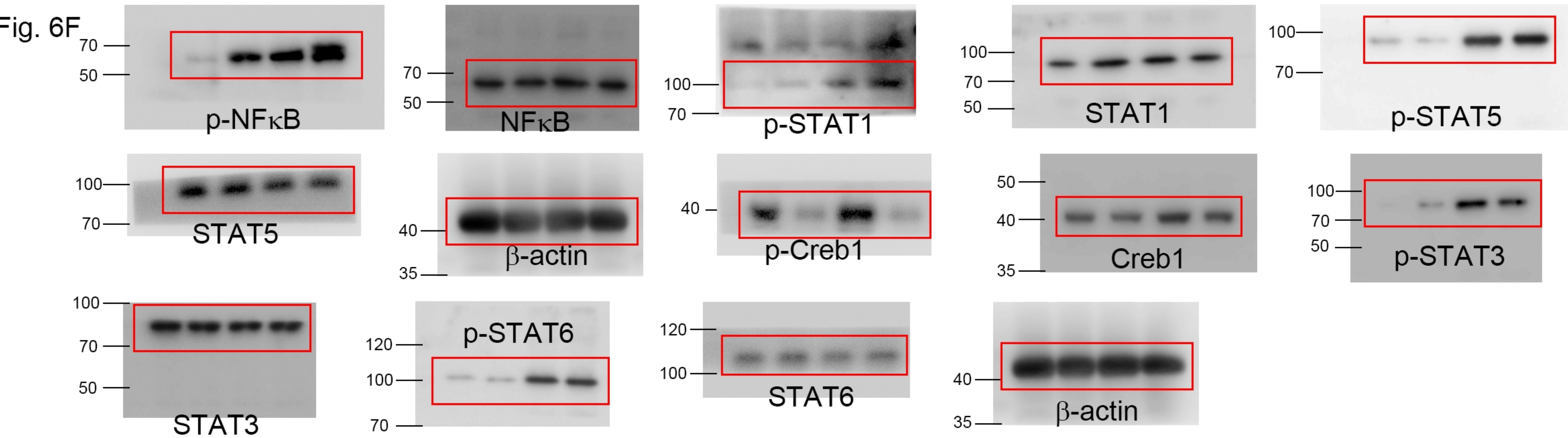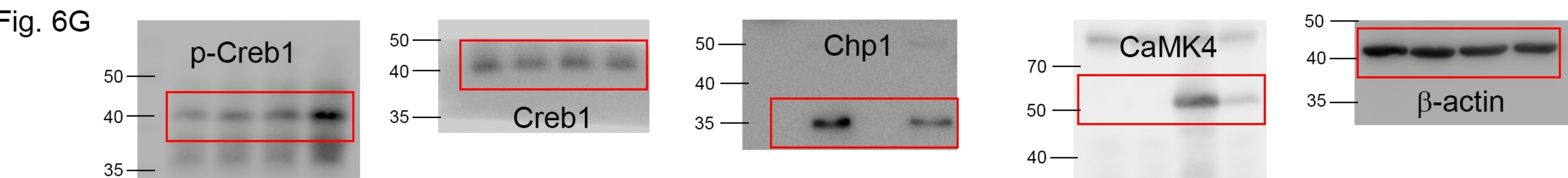

Supplement: Supplementary file 11 — Supporting Information [file ADVS-13-e02395-s004.zip › WB-RawData (2).pdf]

Supplementary Fig. 7C

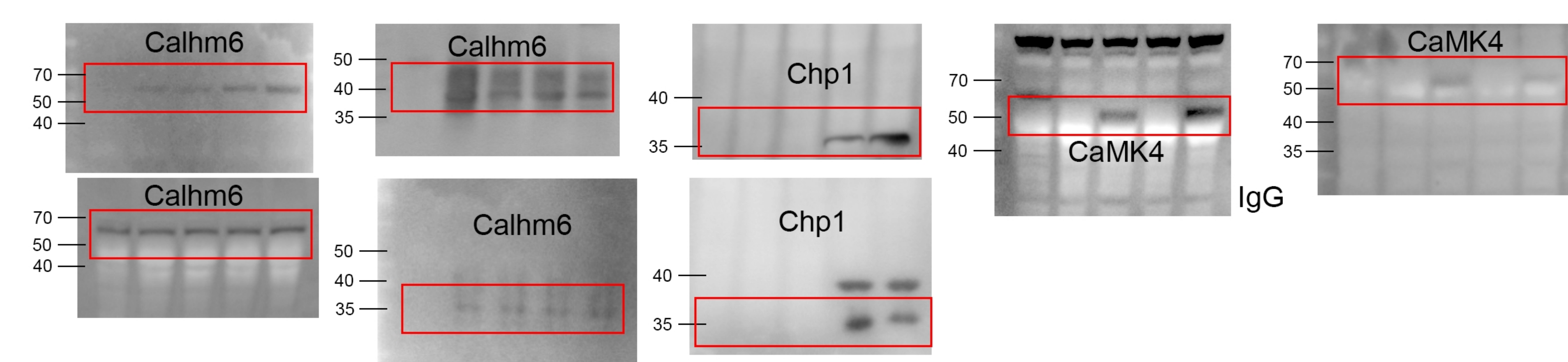

Supplementary Fig. 7D

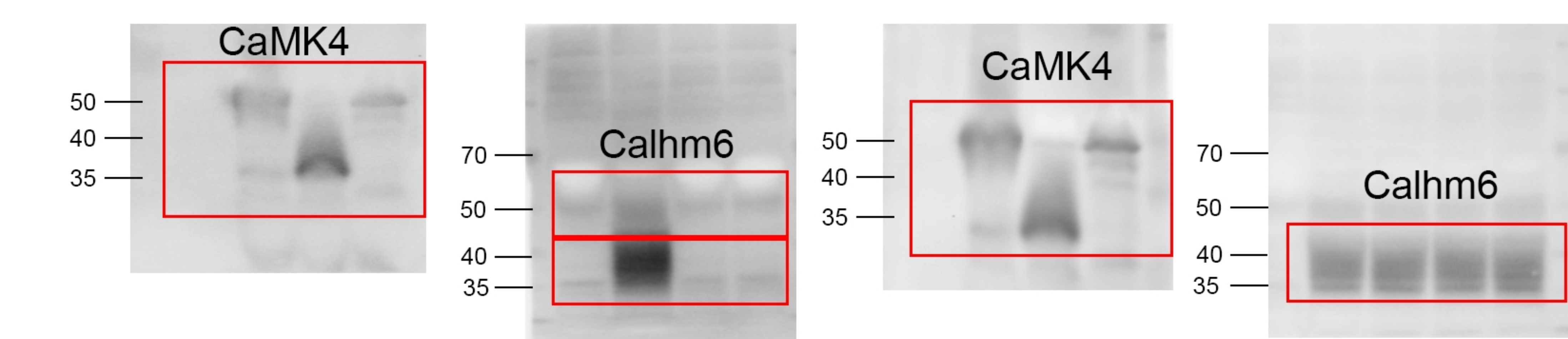

Supplementary Fig. 7E

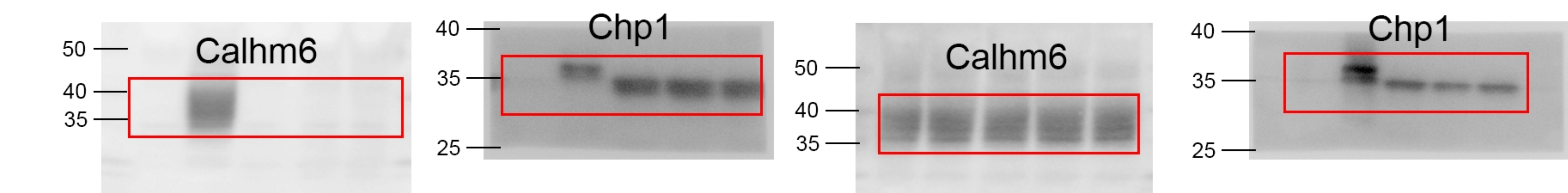

Supplement: Supplementary file 11 — Supporting Information [file ADVS-13-e02395-s004.zip › WB-RawData (7).pdf]

Fig. 7D

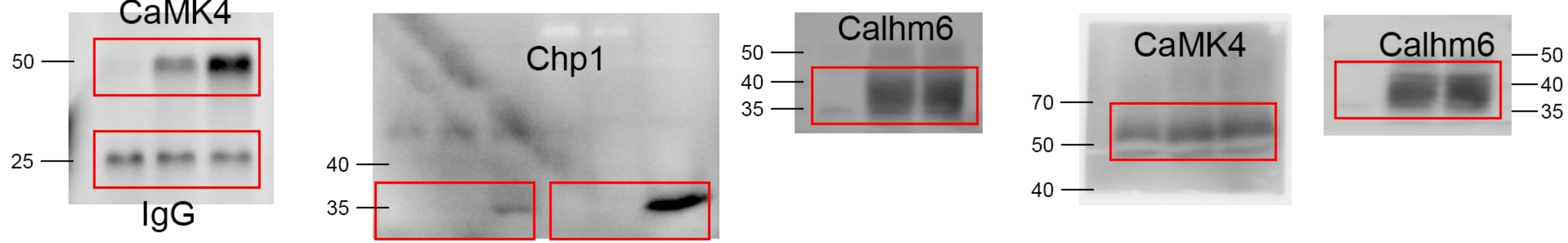

Fig. 7E

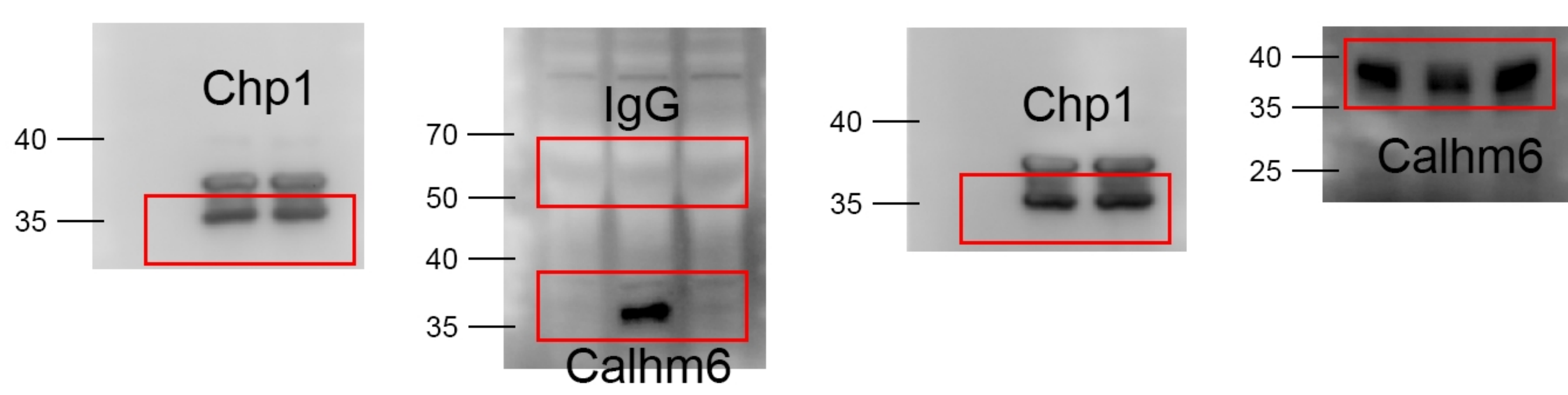

Fig. 7F

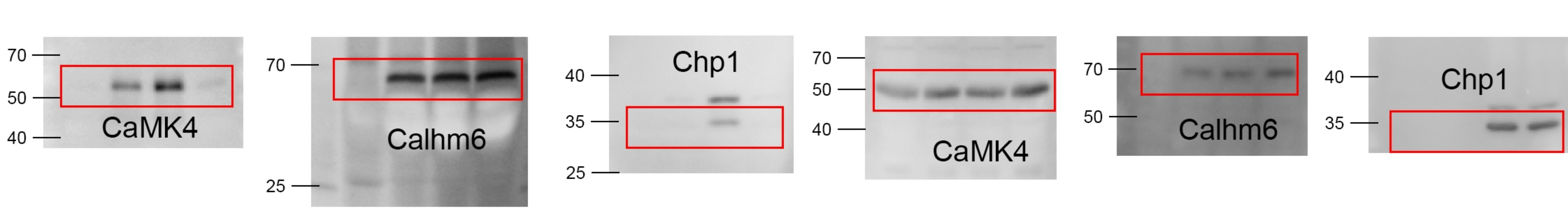

Fig. 7G

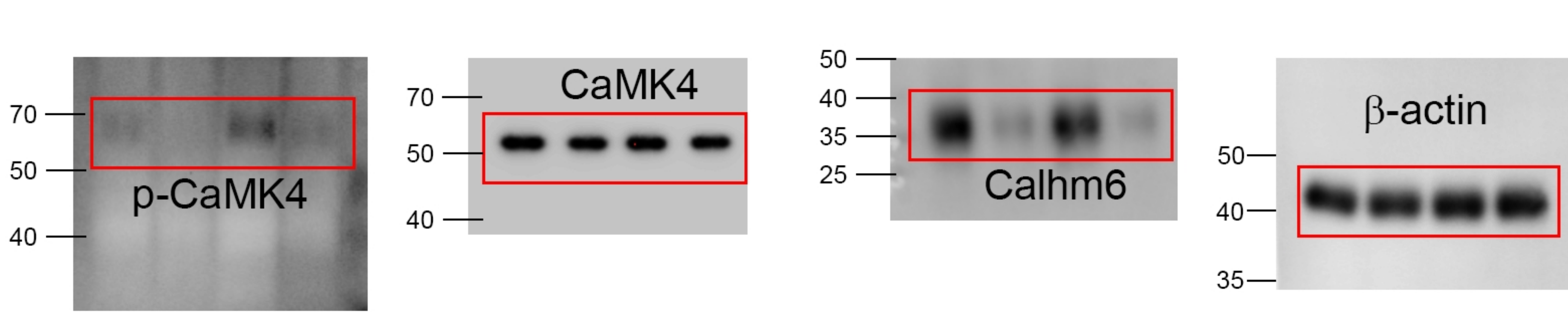

Fig. 7H

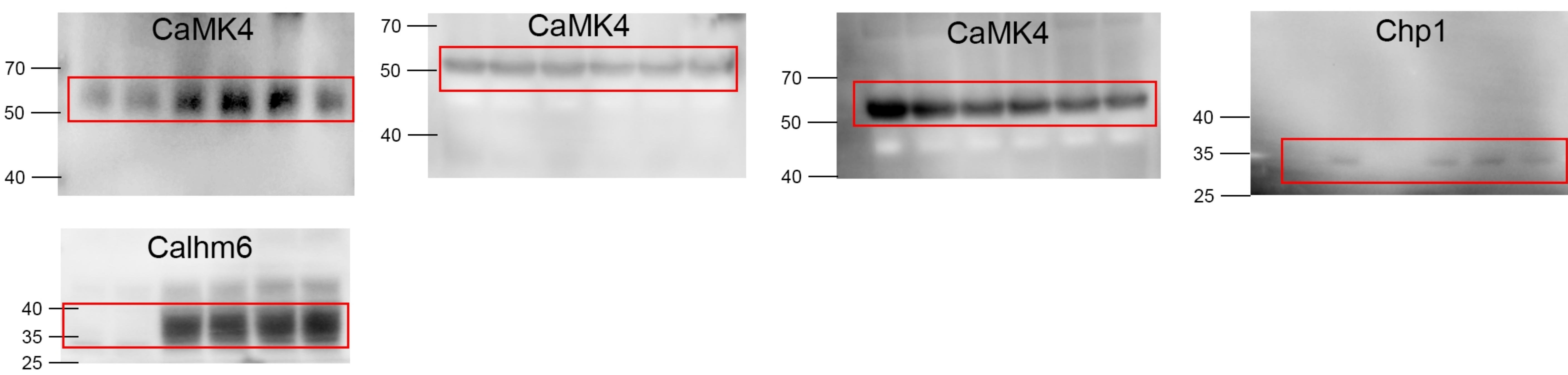

Fig. 7I

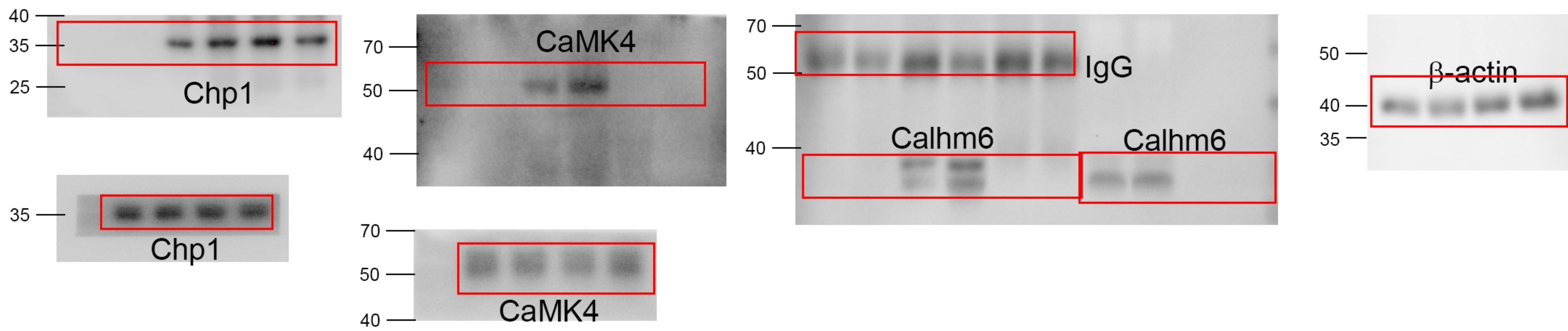

Fig. 7J

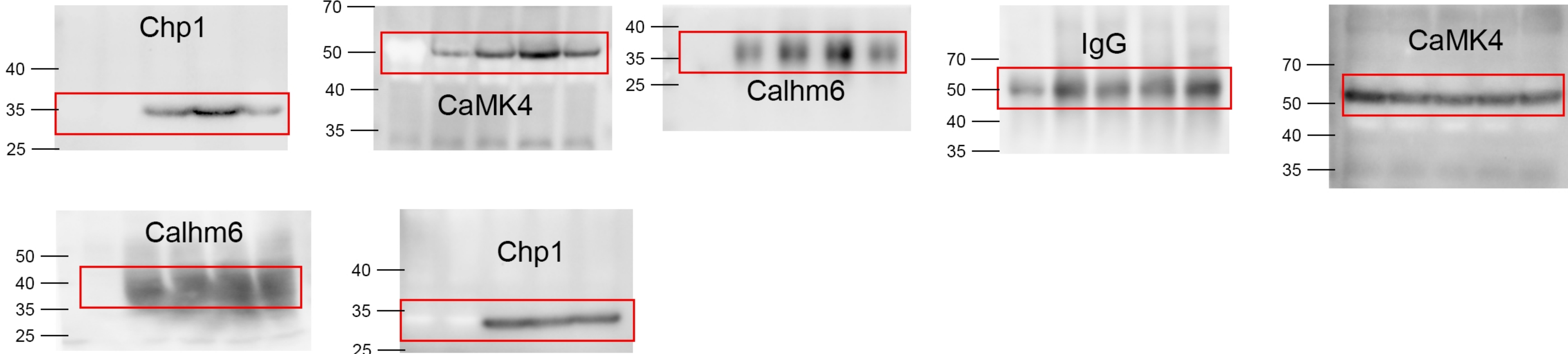

Supplement: Supplementary file 11 — Supporting Information [file ADVS-13-e02395-s004.zip › WB-RawData (4).pdf]

Fig. 7K

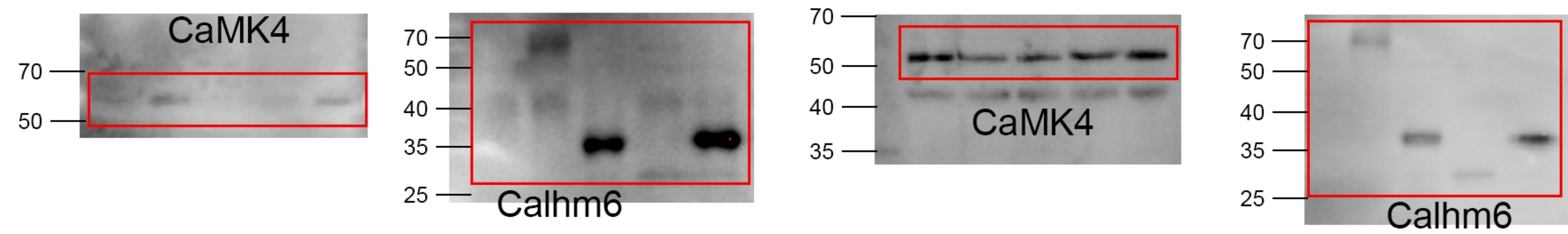

Fig. 7L

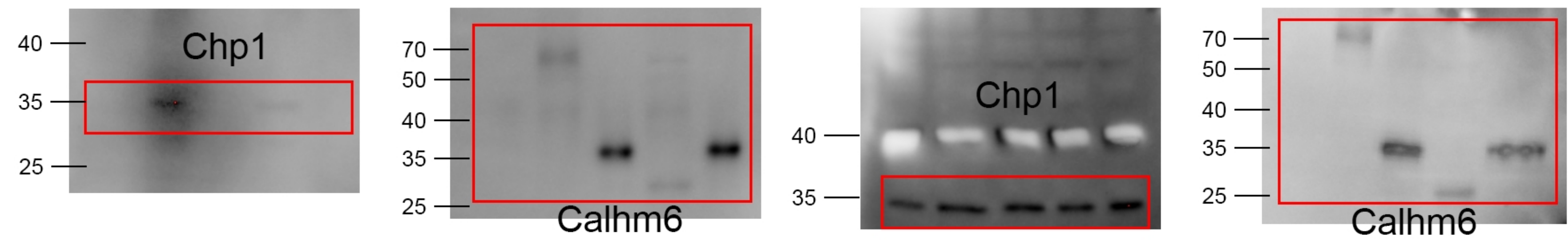

Fig. 8H

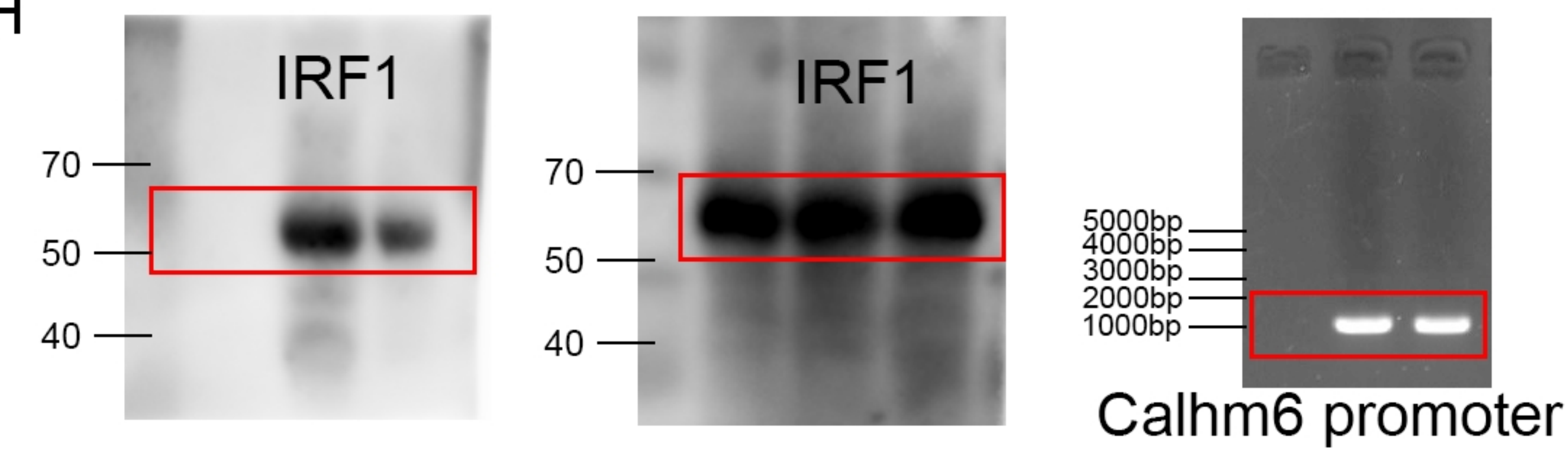

Fig. 8M

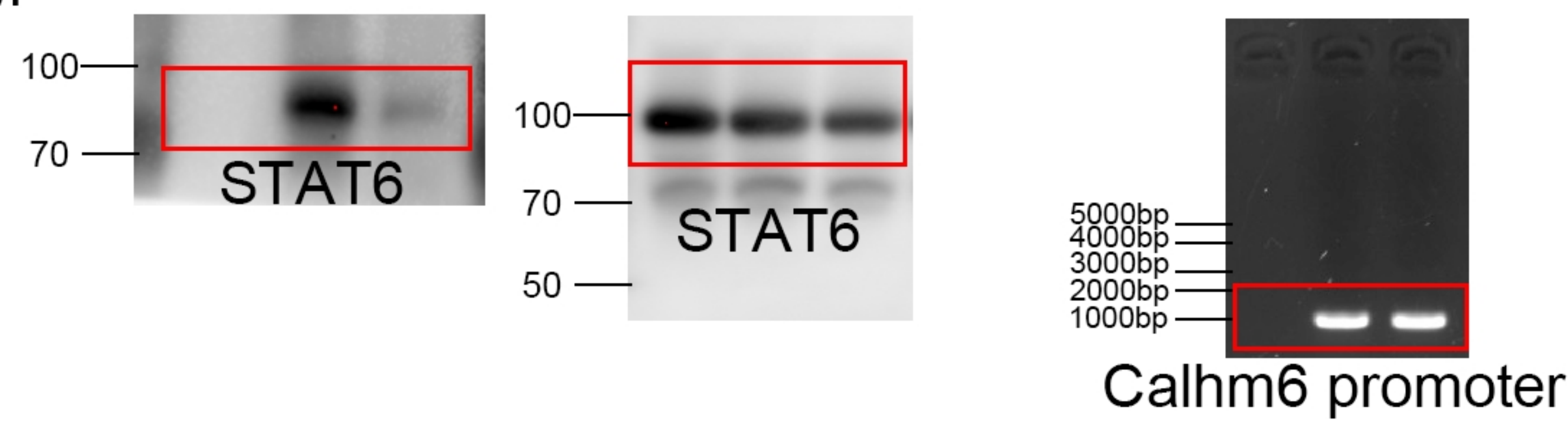

Fig. 8N

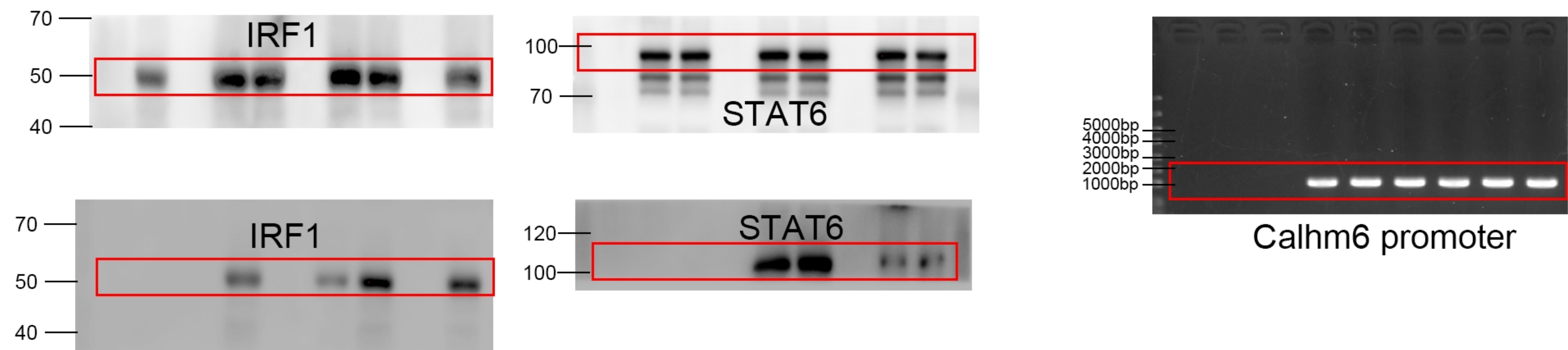

Fig. 8O

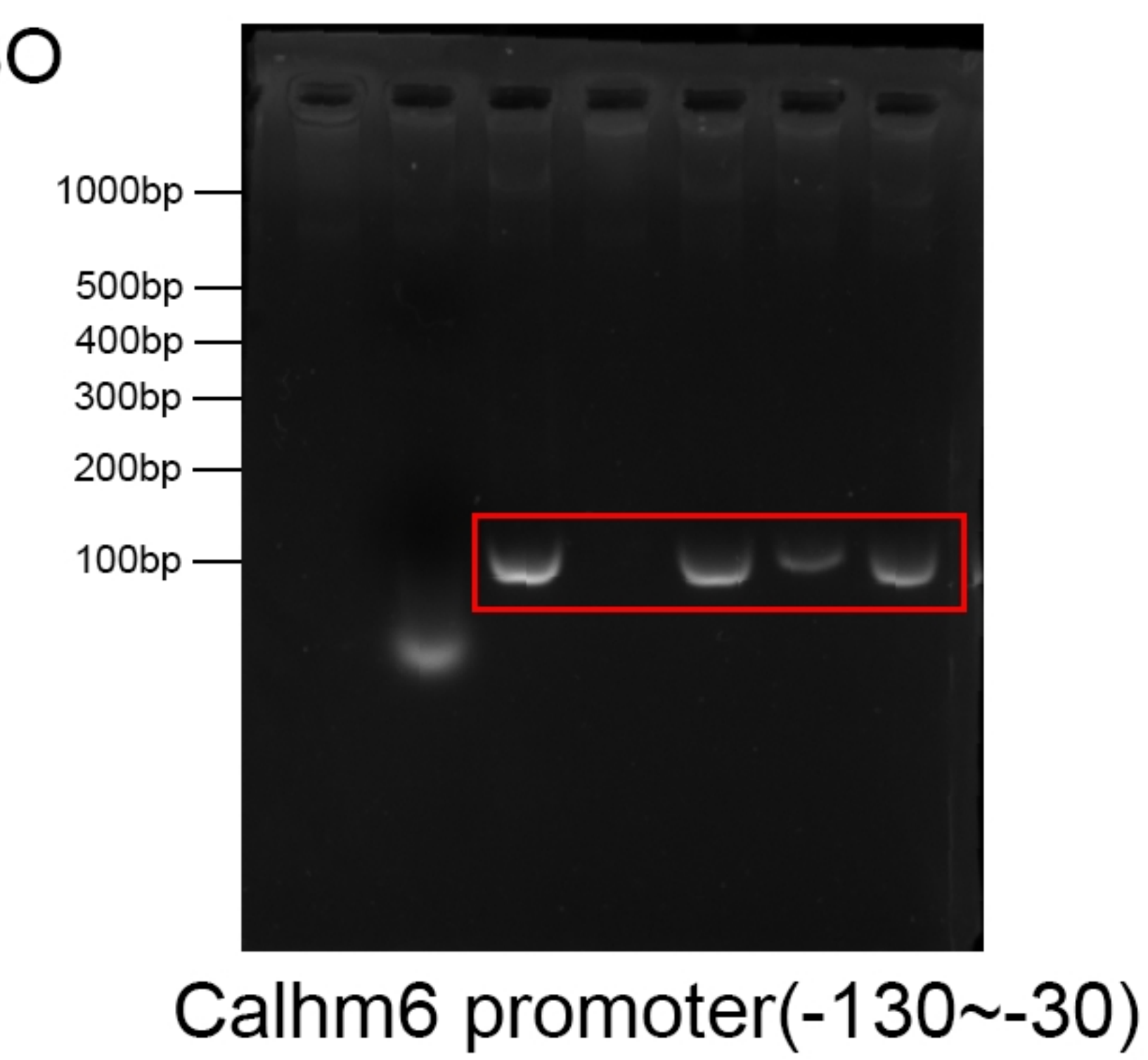

Supplement: Supplementary file 11 — Supporting Information [file ADVS-13-e02395-s004.zip › WB-RawData (5).pdf]

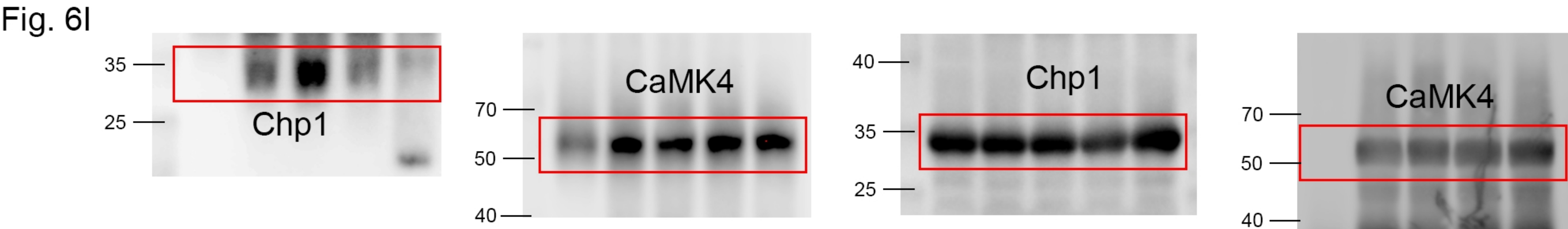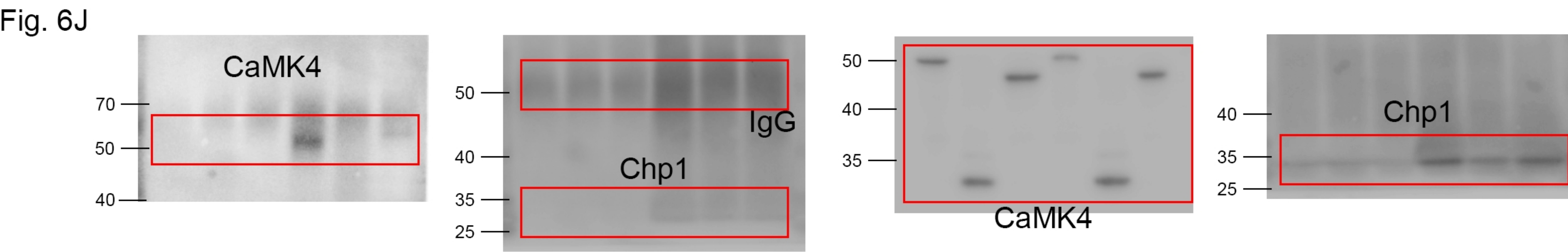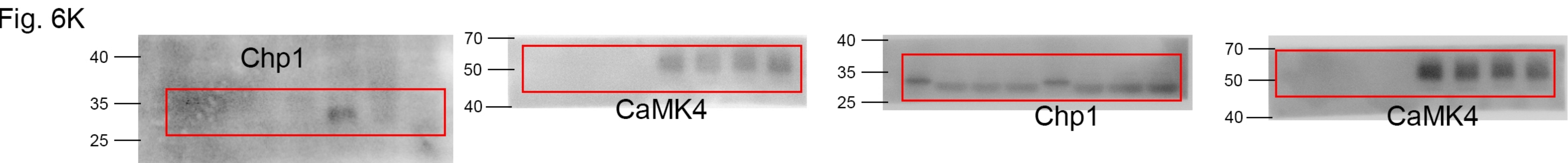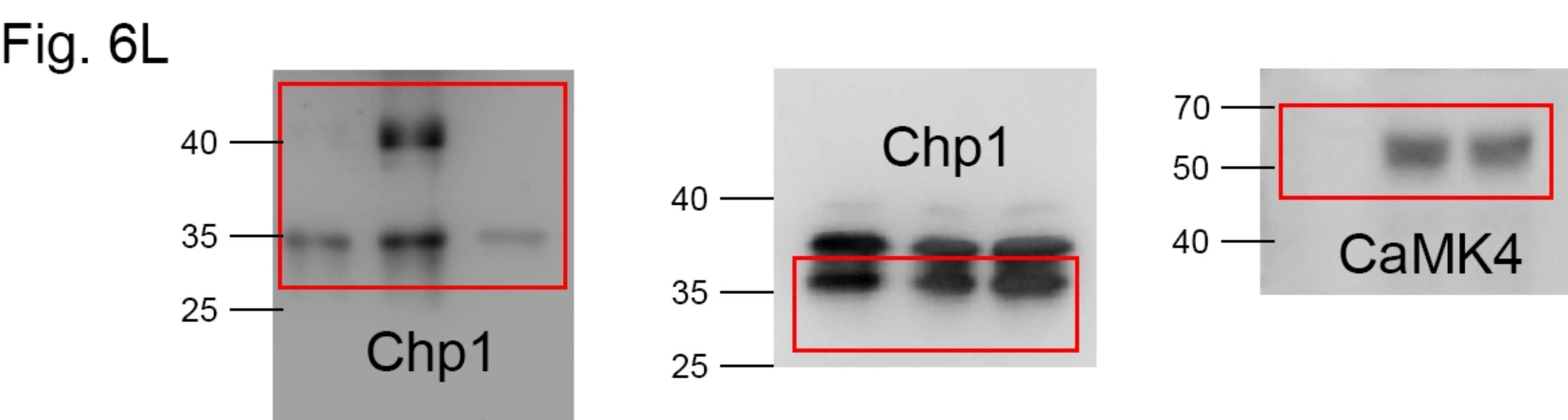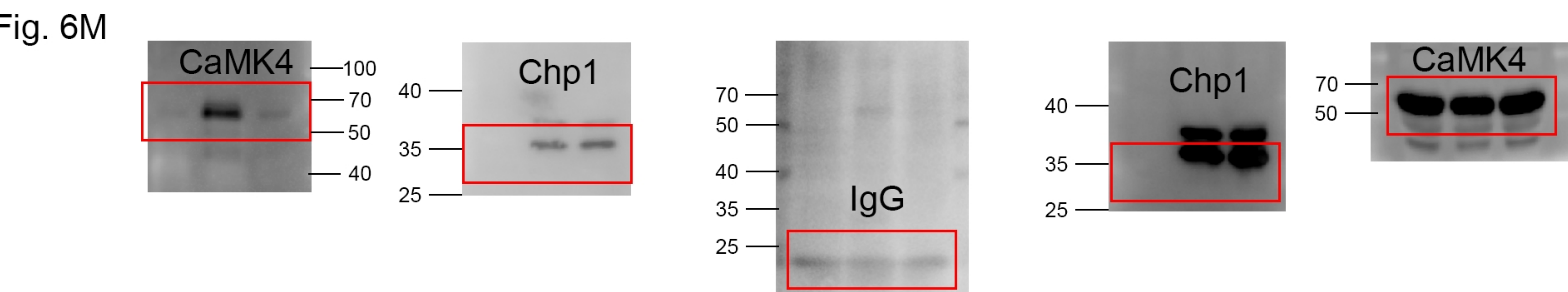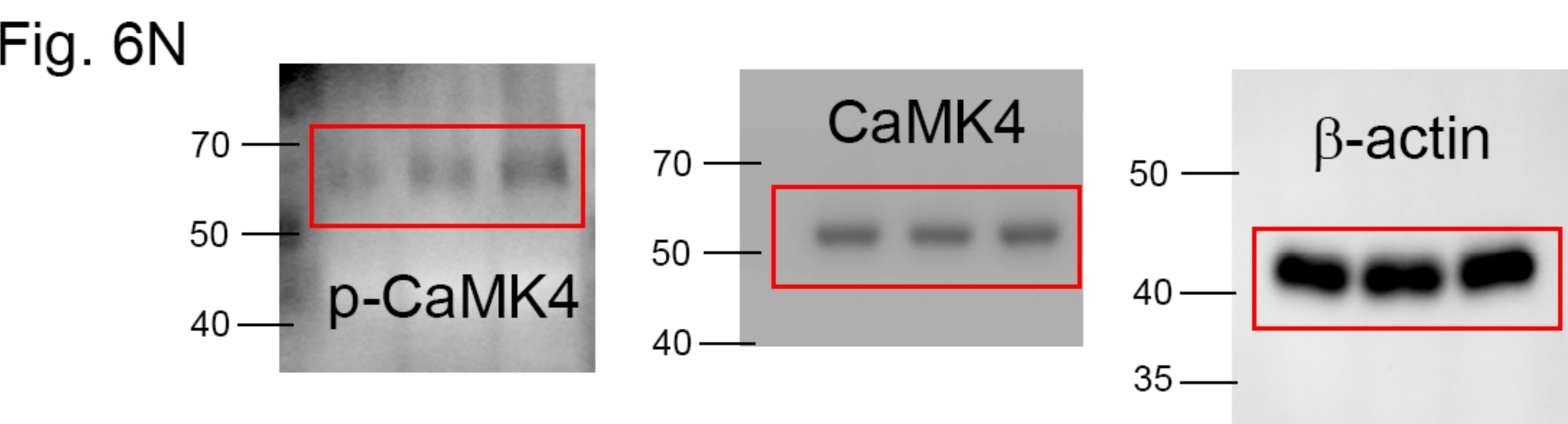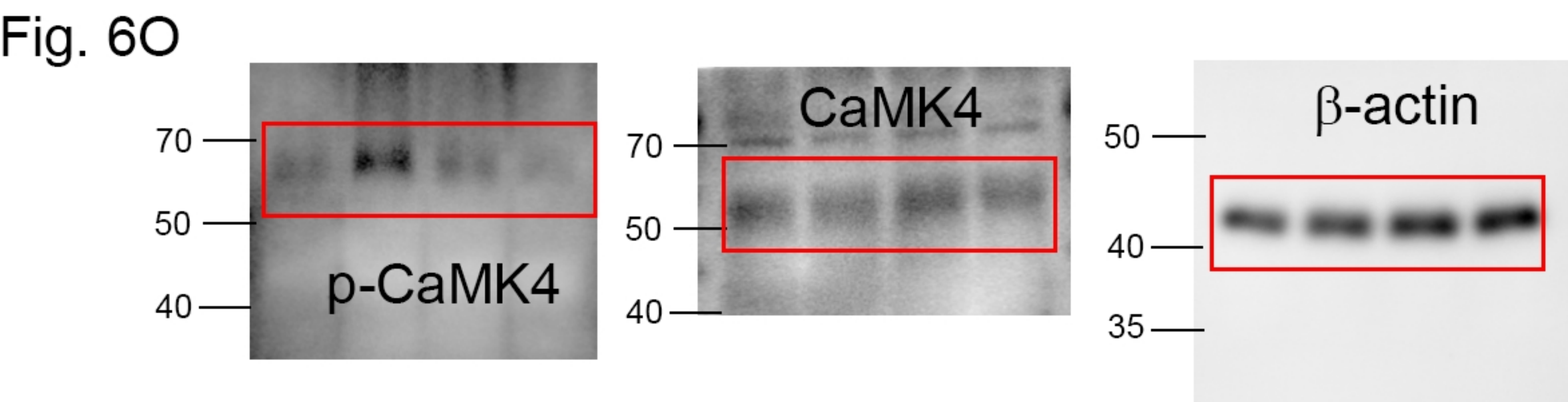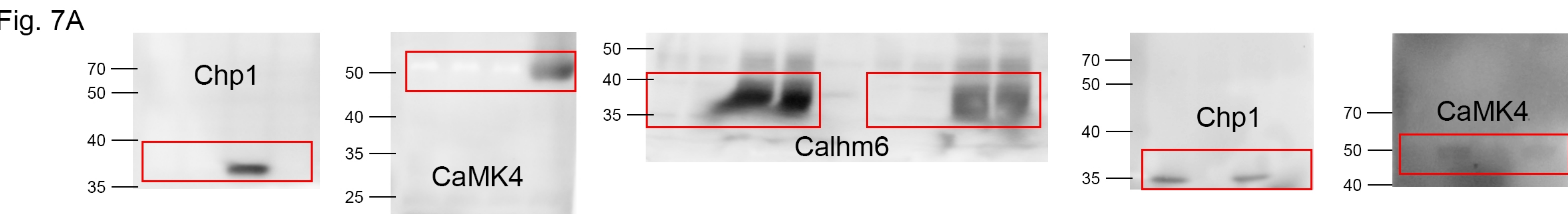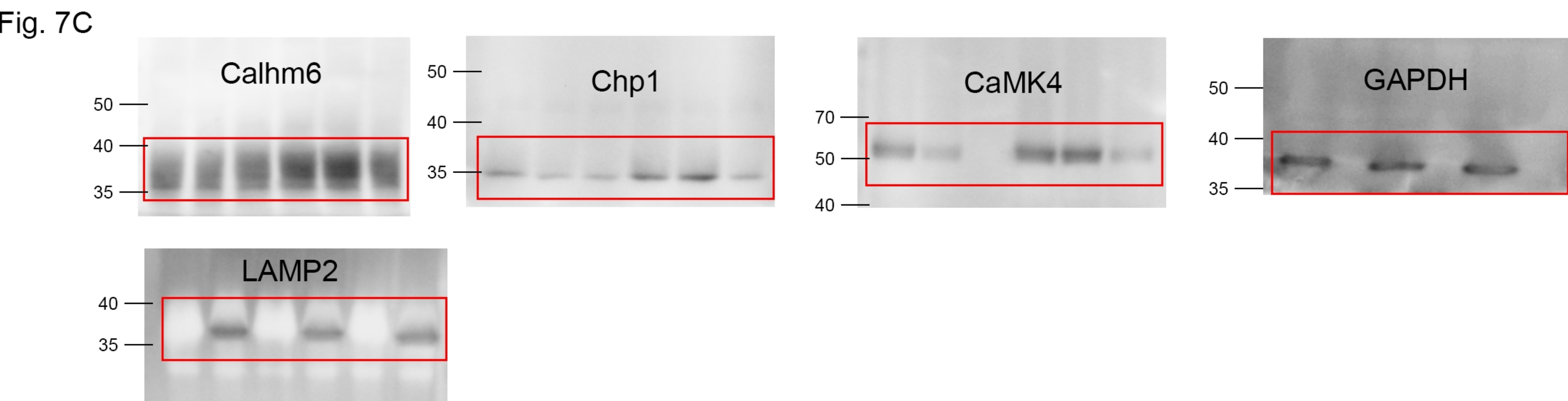

Supplement: Supplementary file 11 — Supporting Information [file ADVS-13-e02395-s004.zip › WB-RawData (3).pdf]

Fig. 1F

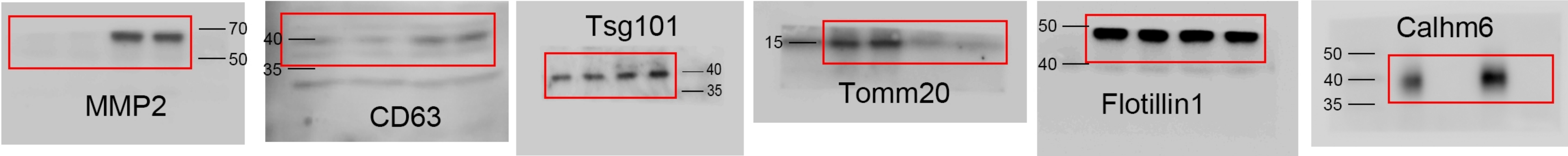

Fig. 1H

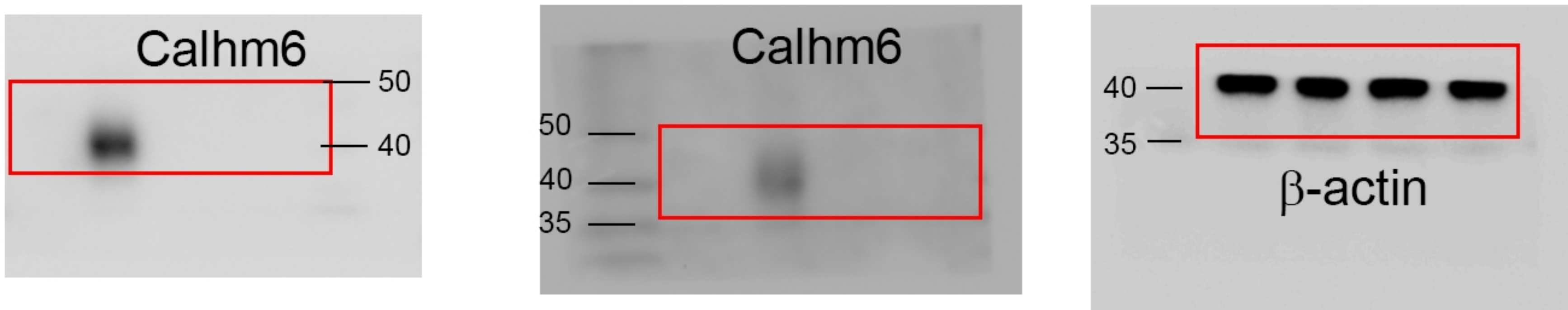

Fig. 2C

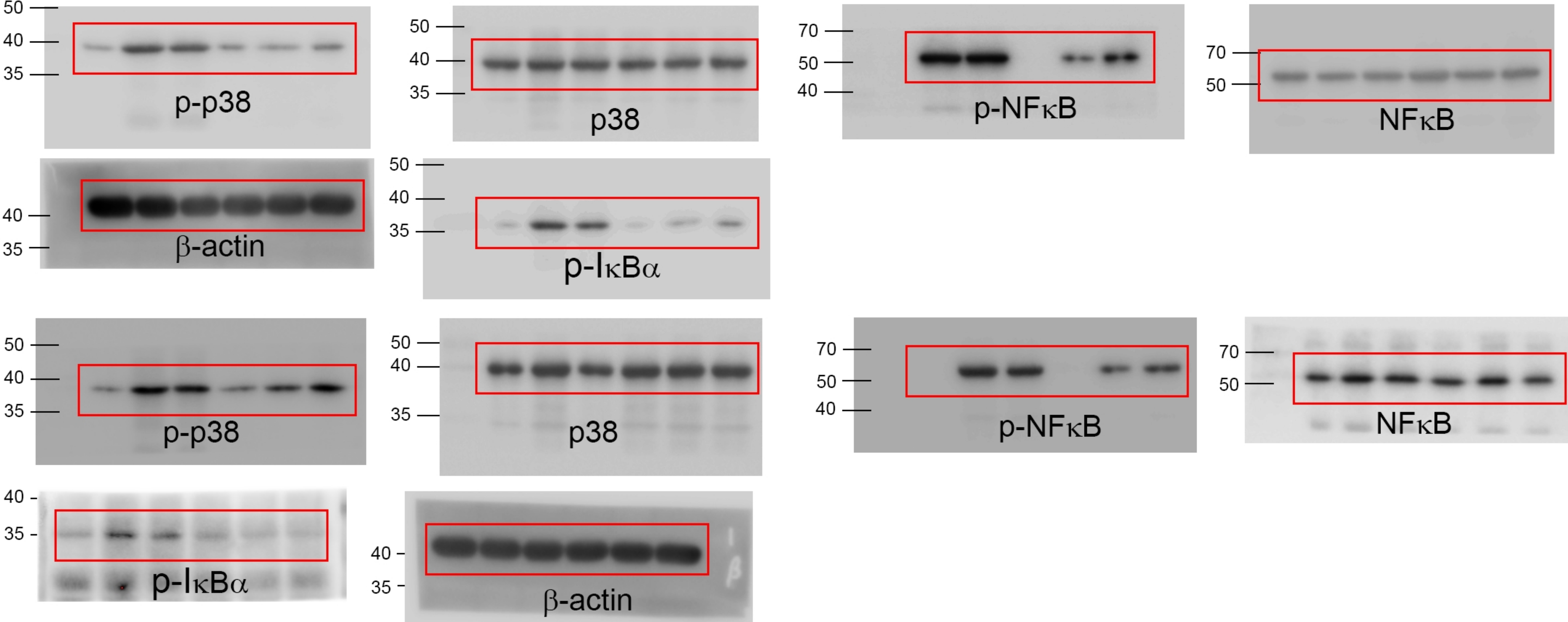

Fig. 2O

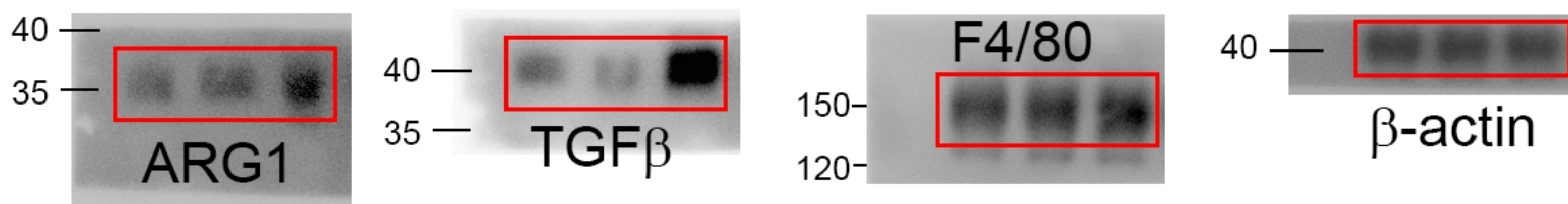

Fig. 3I

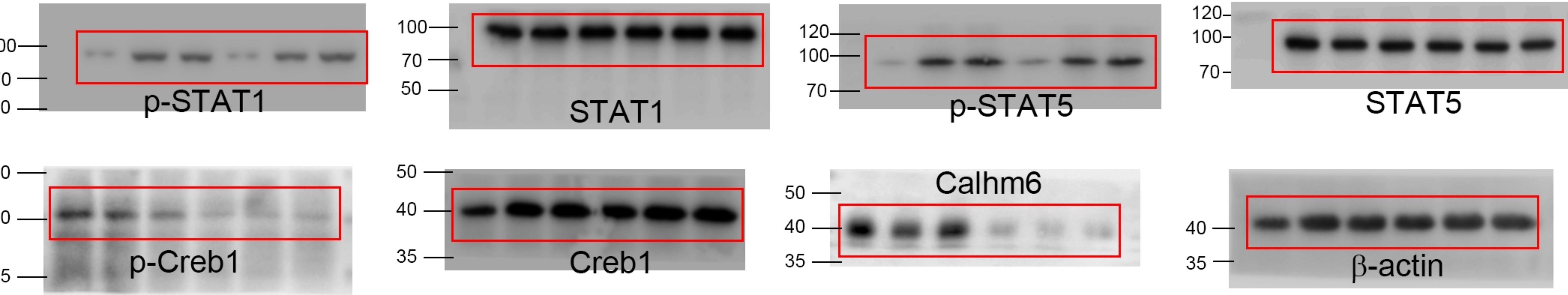

Fig. 3J

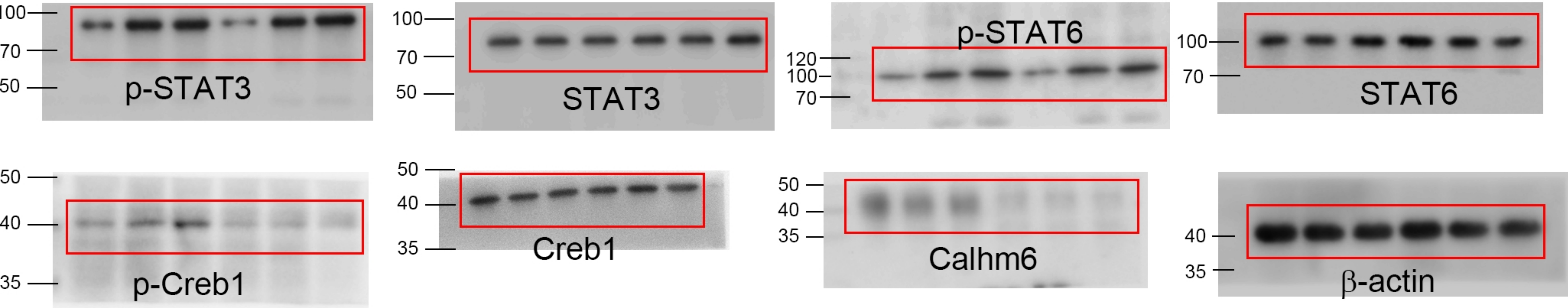

Fig. 3L

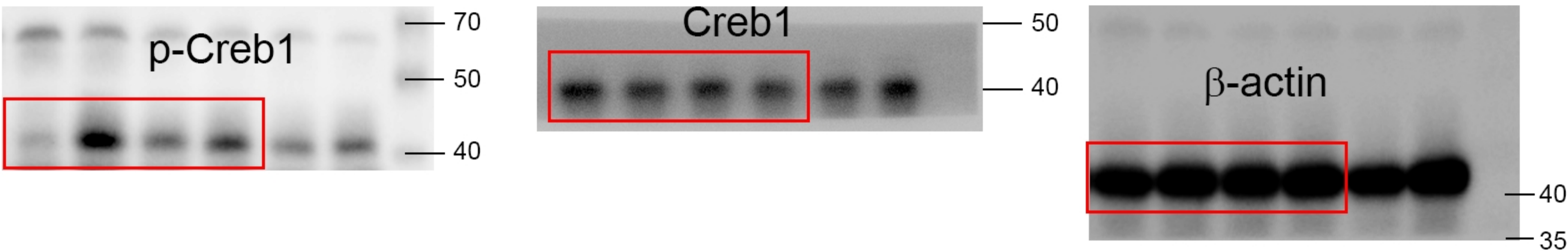

Supplement: Supplementary file 11 — Supporting Information [file ADVS-13-e02395-s004.zip › WB-RawData (1).pdf]
