# Supplementary material for: Calhm6 Governs Macrophage Polarization Through Chp1‐Camk4‐Creb1 Axis and Ectosomal Delivery in Inflammatory Responses
Source: Adv Sci (Weinh). 2025 Sep 26;13(1):e02395. doi: 10.1002/advs.202502395 (PMC12766987; doi:10.1002/advs.202502395)

Supplementary Fig. 1C

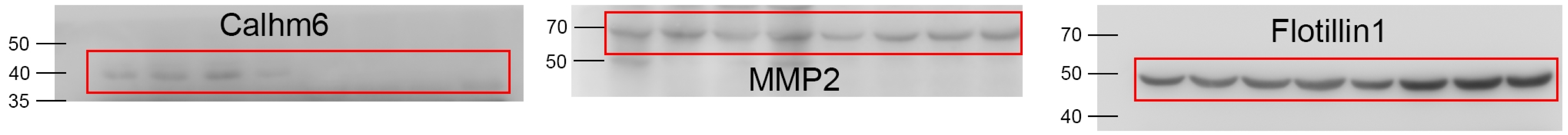

Supplementary Fig. 2A

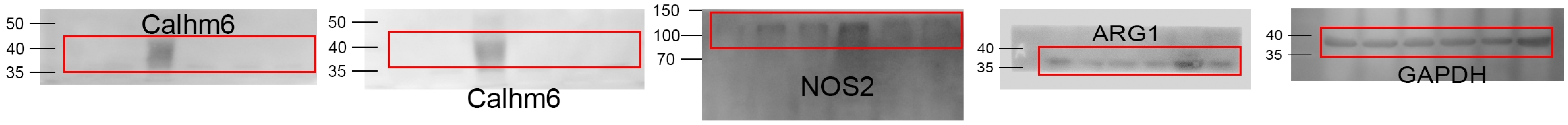

Supplementary Fig. 5A

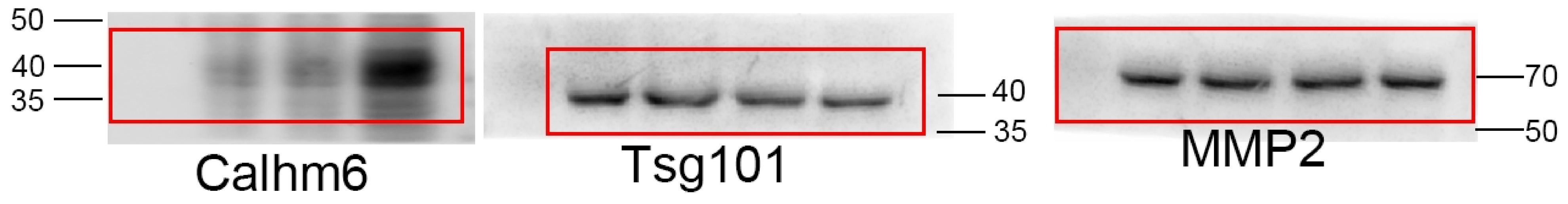

Supplementary Fig. 5D

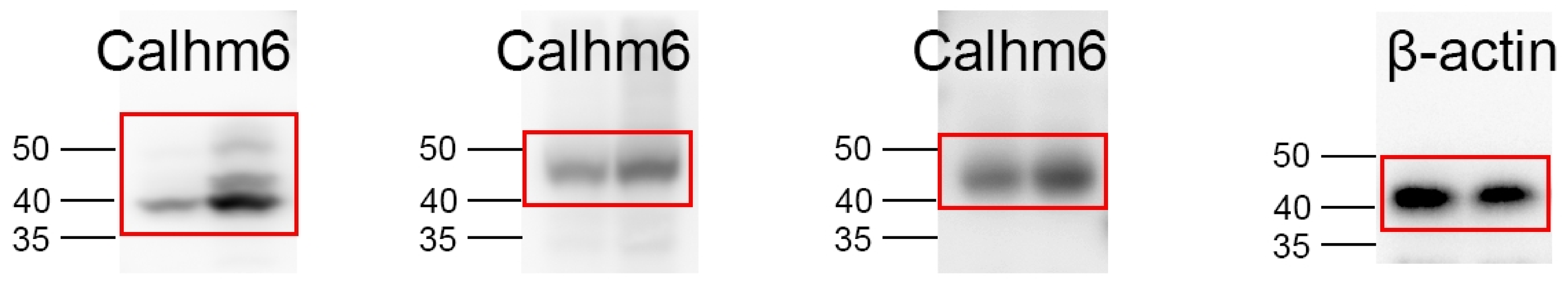

Supplementary Fig. 5E

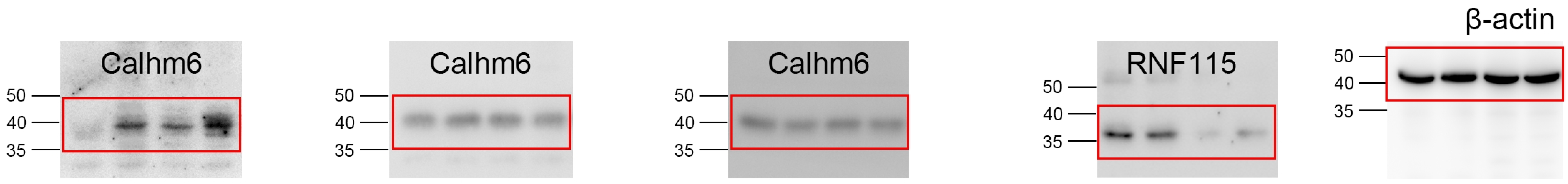

Supplementary Fig. 5J

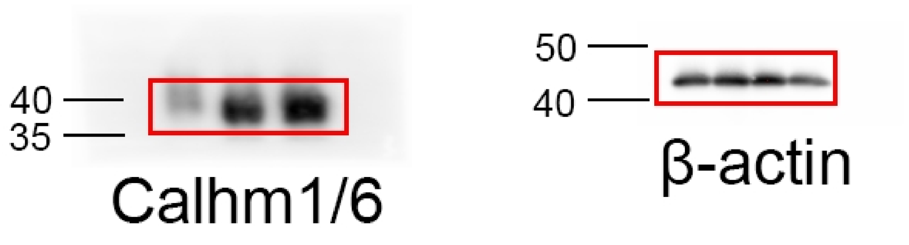

Supplementary Fig. 5O

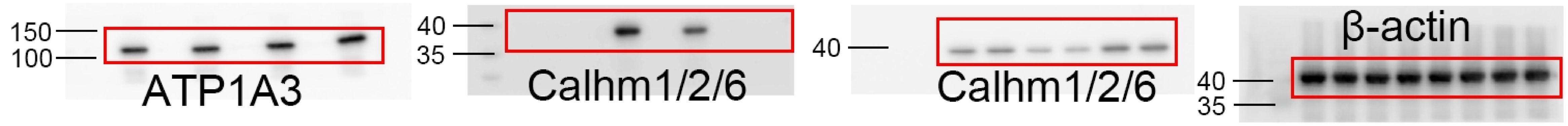

Supplementary Fig. 5P

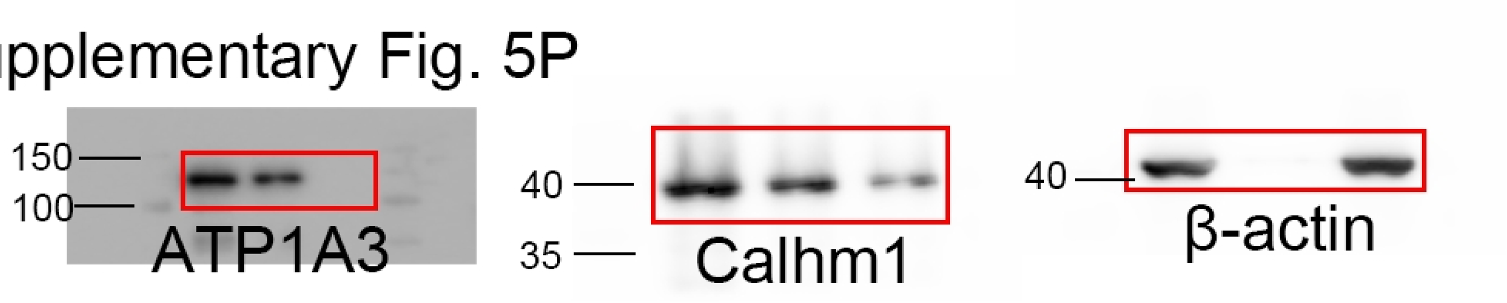

Supplementary Fig. 5Q

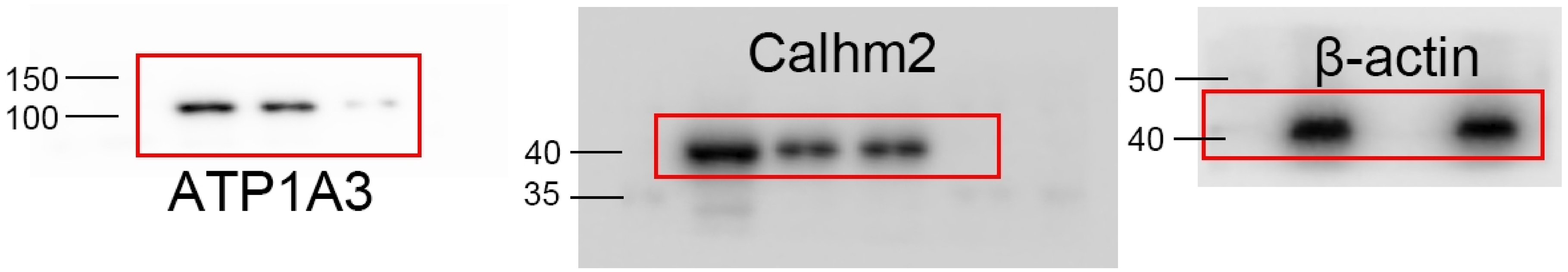

Supplementary Fig. 5R

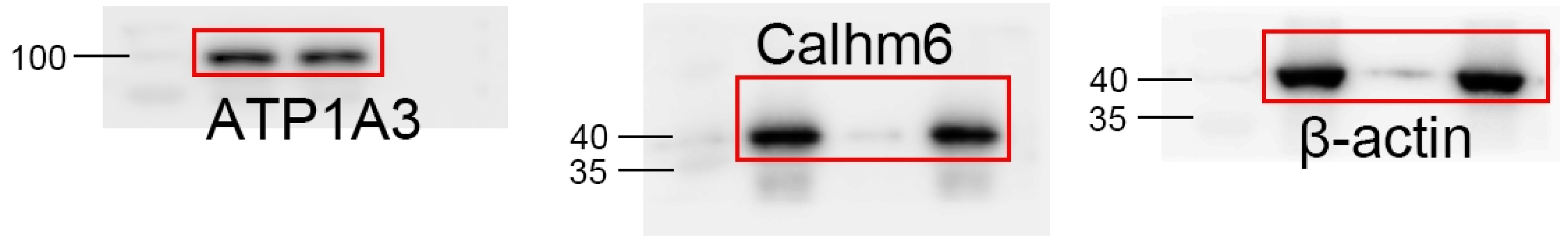

Supplement: Supplementary file 11 — Supporting Information [file ADVS-13-e02395-s004.zip › WB-RawData (6).pdf]
